# Supplementary material for: Sleeping sound with autism spectrum disorder (ASD): study protocol for an efficacy randomised controlled trial of a tailored brief behavioural sleep intervention for ASD
Source: BMJ Open. 2019 Nov 19;9(11):e029767. doi: 10.1136/bmjopen-2019-029767 (PMC6887021; doi:10.1136/bmjopen-2019-029767)
Supplement: Supplementary data [file bmjopen-2019-029767supp001.pdf]

Appendix

Participant Information Sheet and Consent Form – Parent Version

Participant Information Sheet/Consent Form – Parent/Guardian

Interventional Study - *Parent/Guardian consenting on behalf of participant*

**HREC Project Number:**	HREC/16/RCHM/73		
**Research Project Title:**	**Sleeping Sound with Autism Spectrum Disorder (ASD):** Tailoring a behavioural sleep intervention for children with ASD: a randomized controlled trial		
**Principal Researcher:**	Professor Nicole Rinehart, School of Psychology, Deakin University		
**Version Number:**	7	**Version Date:**	17/9/2017

Thank you for taking the time to read this **Parent/Guardian Information Statement and Consent Form**. We would like to invite your child to participate in a research project that is explained below. This document is 5 pages long. Please make sure you have all the pages.

What is an Information Statement?
These pages tell you about the research project. It explains to you clearly and openly all the steps and procedures of the project. The information is to help you decide whether or not you would like your child to take part in the research. Please read this Information Statement carefully.

Before you decide if you want your child to take part or not, you can ask us any questions you have about the project. You may want to talk about the project with your family, friends or health care worker.

Important things you need to know
- It is your choice whether or not your child can take part in the research. You do not have to agree if you do not want to
- If you decide you do not want your child to take part, it will not affect the treatment and care your child gets at The Royal Children's Hospital or with your paediatrician.

If you would like your child to take part in the research project, please sign the consent form at the end of this information statement. By signing the consent form you are telling us that you:

- understand what you have read
- had a chance to ask questions and received satisfactory answers
- consent to your child taking part in the project

We will give you a copy of this information and consent form to keep.

---

1. What is the research project about?

This research project is about sleep in children with an Autism Spectrum Disorder (ASD). Up to 85% of children with ASD have sleep problems, such as difficulty falling asleep, resisting

going to bed and being tired in the morning. We know sleep problems in children with ASD can be related to poorer child and family functioning.

In 2013, we ran a small study that looked at whether a sleep program improved sleep, child and family wellbeing in children with ASD. We found that a 2-3 session sleep program improved these outcomes. This program has already been shown to be helpful for children with ADHD.

This year, we aim to offer this sleep program to a larger group of children with ASD who have sleep problems. To know whether this program helps, we need to compare children who receive the program with children who do not. We hope about 235 children with ASD and their families will take part in this project.

To take part in the study, children need to be aged between 5 and 12 years of age (or 13 years of age and attending primary school), have problems with sleeping and a diagnosis of ASD without an intellectual disability.

2. Who is funding this research project?

This research is fully funded by the National Health and Medical Research Council.

3. Why is my child being asked to take part?

We are asking you and your child to take part because your child has ASD and is aged between 5 and 12 years (or is 13 years and attending primary school).

4. What does my child need to do in this research project?

Start of study
We will telephone you to tell you more about the project and see if your child is eligible. This will involve asking you some questions about your child's sleep, autism symptoms, wellbeing and daily functioning. This call will be conducted in two phone calls, each of which will take between 20 minutes and 40 minutes. We will also ask to contact your child's paediatrician for confirmation of ASD diagnosis. If your child is not eligible for the study, the data you provide will only be reported in summary form (e.g. a total of 7 children were not eligible for the study as they had an intellectual disability).

If your child is eligible to take part, we will ask you to complete a survey about your child's autism and sleep, and about your child and family's wellbeing. This will take about 20 minutes to do and you can complete it online or send it back to us in the reply-paid envelope provided if you are completing it in hard copy. Once we have received your survey, we will randomly place you in one of two groups. This will be done by chance, similar to tossing a coin, so you will have an equal chance of being in either group. We will send you a letter to let you know which group you have been placed in. What you will do next will depend on which group you are allocated to.

- **Usual Care Group**

If you are in the Usual Care Group, we will contact you again in 3, 6- and 12-months' time and ask you to complete surveys about your child's autism and sleep, and child and family wellbeing. Each survey will take about 20 minutes to fill out.

- **Sleep Program Group**

If you are allocated to the Sleep Program Group, we will offer you two 50-minute visits and a follow up phone call with a clinician (psychologist or paediatrician) who has been trained to help you manage your child's sleep problems. At the sleep visits, the clinician will assess your child's sleep and provide you with some sleep strategies. These visits are free. These visits are done privately with the clinician, yourself and your child at Deakin University (Burwood or Geelong), the Royal Children's Hospital/MCRI (Parkville), or your paediatrician's office – there will be no other families at the visit. We will then send you surveys to complete in 3, 6- and 12-months' time. Each survey asks about your child's ASD and sleep, and child and family wellbeing and will take about 20 minutes to fill out.

6 months after start of study – participants in both groups

We will arrange a time to complete a face-to-face learning assessment with your child. It will take about 60 minutes to do. The assessment is non-invasive and most children enjoy doing it. We can visit you at your home to do this assessment or you can visit us at The Royal Children's Hospital in Melbourne, or Deakin University in Burwood or Geelong. We will provide you with a brief report from this assessment if requested.

5. Optional consent

We would like you to consider the following optional parts of this project. You may agree to none, some or all optional parts. However, it is your decision, and if you choose not to agree to any parts, it will not affect your participation in the rest of the study. Please complete the optional consent tick boxes on the consent form to record your decision for each option.

A. **Contacting your child's school teacher:** We would like to ask your child's teacher to fill out a short survey about your child's behaviour to find out if sleep affects your child at school. We will ask them to complete the survey on four occasions. We will contact the kindergarten director or school principal and gain their approval to ask your child's teacher to do this. We will not refer to ASD in the information we send to the teacher or the principal/director. Instead, we will say that this is a project about managing sleep problems in children. The information provided by the teacher in the survey will not be shared with you or your child.

B. **NAPLAN.** We would like your permission to get your child's NAPLAN results. Your child's NAPLAN results will be de-identified and stored in a password-secured database, only accessible by the research team. Please indicate on the consent form if you consent to us getting your child's NAPLAN results.

C. **Assessment records.** We would like to access the results of any assessments related to ASD which your child may have done in the past, including autism assessments, IQ, adaptive functioning and language assessments. We will use this information to help us understand whether the intervention is useful for children with a range of abilities. If you do not have copies of these reports yourself, with your permission we will request them from health professionals or hospitals your child has visited.

D. **Re-contact for future research:** We would like to offer you the chance to hear about new research projects that are related to this project. By ticking this consent you are only consenting to hear about new projects only. You can decide at the time whether or not you want your child to take part in any future project.

E. **Medicare and Pharmaceutical Benefits Scheme (PBS):** We would like to access your child's complete Medicare and PBS data. This will help us to see whether families who get the sleep program access fewer health services and medicines than those who

do not get the program. Medicare collects information on your child's doctors' visits and the associated costs, while the PBS collects information on the prescription medications you have filled at pharmacies for your child. We will ask you to fill out this additional consent form at the time of your child's 6 month follow up, which will allow us to access this data as outlined on the back of the additional consent form we will give you at that time. This consent form will then be sent securely to the Department of Human Services who holds this information confidentially.

6. Can my child withdraw from the project?

If you give your consent and change your mind, your child can withdraw from the project. If you decide to withdraw, please notify the research team. You do not need to tell us the reason why you or your child want to stop being in the project. If your child leaves the project we will use any information already collected unless you tell us not to do so.

7. What are the possible benefits for my child and other people in the future?

If you are in the **Sleep Program Group**, the clinician will provide strategies and information to help you with your child's sleep problem. If your child has other difficulties other than sleep problems (e.g. anxiety, ADHD symptoms), these will not be directly addressed by the Sleeping Sound intervention – the study clinician will encourage you to discuss these problems with your treating paediatrician. We hope that your child will have fewer sleep problems and this may help his/her behaviour as well as your child and family's wellbeing.

If you are in the **Usual Care Group**, you and your child will not receive any direct benefit.

We are doing this project to see if this sleep program works or not. This means that we can't guarantee that your child and family will benefit, even if you are in the **Sleep Program Group.**

Whether you receive the sleep program or not, your participation will help us learn whether the sleep program is helpful for families of children with ASD. If it is helpful, then the project will be the first to provide information which may help future children with sleep problems and ASD.

8. What are the possible risks, side-effects, discomforts and/or inconveniences?

We do not anticipate risks, side-effects or discomforts for you or your child. In our previous *Sleeping Sound with ADHD* project, parents did not report any side-effects or discomforts in their feedback surveys.

9. What will be done to make sure my child's information is confidential?

In this study we will collect and use personal and health information about your child for research purposes. Any information we collect that can identify your child will be treated as confidential. It will be used only in this project, unless otherwise specified. We can disclose the information only with your permission, except as required by law. All information will be stored securely at Deakin University. Information will be kept electronically on a secure Deakin University server, in a password protected database (REDCap) or password protected computer. All hard copy consent forms, surveys and assessments will be stored securely in a locked cabinet at Deakin University.

The following people may access information collected as part of this research project:

- The research team involved with this project
- The Royal Children's Hospital Human Research Ethics Committee

The information will be re-identifiable. This means that we will remove your child's name and give the information a special code number. Only the research team can match your child's name to their code number, if it is necessary to do so.

Information will be kept until the youngest participant turns 25 years old. The research information may be destroyed or kept indefinitely in secure storage after this time. However, Medicare/PBS data will be kept for 15 years only from the date of collection. After this time, the consent paper copies will be shredded in a manner that makes re-identification of the data impossible, and the electronic Medicare and PBS data will be deleted.

In accordance with relevant Australian and/or Victorian privacy and other relevant laws, you have the right to access and correct the information we collect and store about your child. Please contact us if you would like to access this information.

At the end of the study, results may be presented at conferences or published in medical journals. This will be done in such a way that your child cannot be identified.

10. Will we be informed of the results when the research project is finished?

Participants in the Sleep Program group and the Usual Care group will receive a summary of the results at the end of this project, which will say whether the intervention was successful. This summary will give overall results of this project – individual families will not be identified. Participants in the Usual Care group **will not** be offered the Sleeping Sound intervention after completion of the study.

11. Who should I contact for more information?

If you would like more information about the project or if you need to speak to a member of the research team in an emergency please contact:

**Name:**	Susannah Bellows
**Contact telephone:**	9246 8937
**Email:**	sleepingsound@deakin.edu.au

If you have any concerns and/or complaints about the project, the way it is being conducted or your child's rights as a research participant, and would like to speak to someone independent of the project, please contact: Director, Research Ethics & Governance, The Royal Children's Hospital Melbourne on telephone: (03) 9345 5044.

CONSENT FORM

**HREC Project Number:**	HREC/16/RCHM/73
**Research Project Title:**	Sleeping Sound with Autism Spectrum Disorder (ASD):<br>Tailoring a behavioural sleep intervention for children with ASD: a randomized controlled trial
**Version Number:** 7	**Version Date:** 17/09/2017

- I have read, or someone has read to me in a language that I understand, the information statement version listed above and I understand its contents.
- I believe I understand the purpose, extent and possible risks of our involvement in this project.
- I voluntarily consent for me and my child to take part in this research project.
- I have had an opportunity to ask questions and I am satisfied with the answers I have received.
- I understand that this project has been approved by The Royal Children's Hospital Melbourne Human Research Ethics Committee and will be carried out in line with the National Statement on Ethical Conduct in Human Research (2007) – including all updates.
- I understand I will receive a copy of this Information Statement and Consent Form.

OPTIONAL CONSENT

☐ I do	☐ I do not	consent for the researchers to contact my child's school teacher to complete a survey
☐ I do	☐ I do not	consent for VCAA (Victorian Curriculum and Assessment Authority) to release my child's years 3, 5, and 7 NAPLAN results to the Sleeping Sound with ASD Project.
☐ I do	☐ I do not	consent for the researchers to access copies of my child's assessments and medical records related to autism
☐ I do	☐ I do not	consent to be contacted about future research projects that are related to this project.
☐ I do	☐ I do not	consent to the storage and use of my child's information in future ethically-approved research related to this research project.

____
Child's Name

Parent/Guardian Name	Parent/Guardian Signature	Date
Name of Witness to Parent/Guardian's Signature	Witness Signature	Date

Declaration by researcher: I have explained the project to the parent/guardian who has signed above, and believe that they understand the purpose, extent and possible risks of their and their child's involvement in this project.

Research Team Member Name	Research Team Member Signature	Date

Note: All parties signing the Consent Form must date their own signature.
