# Supplementary material for: Sleeping sound with autism spectrum disorder (ASD): study protocol for an efficacy randomised controlled trial of a tailored brief behavioural sleep intervention for ASD
Source: BMJ Open. 2019 Nov 19;9(11):e029767. doi: 10.1136/bmjopen-2019-029767 (PMC6887021; doi:10.1136/bmjopen-2019-029767)
Supplement: Supplementary data [file bmjopen-2019-029767supp002.pdf]

Cluster effect 1 - paediatrician					Cluster effect 2 - individual						
ICC	Mean	SD	CV	DE1 = 1+(n-1)ρ	ICC	Mean	SD	CV	DE2 = 1+(n-1)ρ	z_beta (80%)	z_alpha (.05, two tailed)
0.08	4	3	0.75	1.24	0.2	2	1	0.50	1.20	0.84	1.96

			Sample size (total)				
Attrition	d	var_d	n_total=2*((2*sigma^2 *(z_beta+z_alpha)^2)/(d^2))	DE1 adjusted N	DE2 adjusted N	Attrition adjusted	Per group
0.20	0.50	1	125	156	187	233	117
