# Supplementary material for: Sleeping sound with autism spectrum disorder (ASD): study protocol for an efficacy randomised controlled trial of a tailored brief behavioural sleep intervention for ASD
Source: BMJ Open. 2019 Nov 19;9(11):e029767. doi: 10.1136/bmjopen-2019-029767 (PMC6887021; doi:10.1136/bmjopen-2019-029767)
Supplement: Author's manuscript [file bmjopen-2019-029767.draft_revisions.pdf]

BMJ Open is committed to open peer review. As part of this commitment we make the peer review history of every article we publish publicly available.

When an article is published we post the peer reviewers' comments and the authors' responses online. We also post the versions of the paper that were used during peer review. These are the versions that the peer review comments apply to.

The versions of the paper that follow are the versions that were submitted during the peer review process. They are not the versions of record or the final published versions. They should not be cited or distributed as the published version of this manuscript.

BMJ Open is an open access journal and the full, final, typeset and author-corrected version of record of the manuscript is available on our site with no access controls, subscription charges or pay-per-view fees (http://bmjopen.bmj.com).

If you have any questions on BMJ Open's open peer review process please email info.bmjopen@bmj.com

BMJ Open

Sleeping Sound with Autism Spectrum Disorder (ASD): Study protocol for an efficacy RCT of a tailored brief behavioural sleep intervention for autism spectrum disorder.

Journal:	*BMJ Open*
Manuscript ID	bmjopen-2019-029767
Article Type:	Protocol
Date Submitted by the Author:	11-Feb-2019

Complete List of Authors:	Papadopoulos, Nicole; Deakin University<br>Sciberras, Emma; Deakin University; Murdoch Childrens Research Institute<br>Hiscock, Harriet; Murdoch Childrens Research Institute; The Royal Children’s Hospital<br>Williams, Katrina; Murdoch Childrens Research Institute; University of Melbourne, Department of Paediatrics<br>McGillivray, Jane; Deakin University<br>Mihalopoulos, Cathrine; Deakin University<br>Engel, Lidia; Deakin University<br>Fuller-Tyszkiewicz, Matthew; Deakin University<br>Bellows, Susannah; Deakin University<br>Marks, Deborah; Deakin University; Royal Children's Hospital Melbourne<br>Howlin, Patricia; University of Sydney, Brain and Mind Centre; King`s College London, Institute of Psychiatry, Psychology and Neuroscience<br>Rinehart, Nicole; Deakin University

Keywords:	ASD, Autism Spectrum Disorder, Sleep, Behavioural sleep intervention, RCT

SCHOLARONE™
Manuscripts

Title: Sleeping Sound with Autism Spectrum Disorder (ASD): Study protocol for an efficacy RCT of a tailored brief behavioural sleep intervention for autism spectrum disorder.

Nicole Papadopoulos[1], Emma Sciberras[1,2,3], Harriet Hiscock[2,4], Katrina Williams[2,3,4], Jane McGillivray[1], Cathrine Mihalopoulos[1], Lidia Engel[1], Matthew Fuller-Tyszkiewicz[1], Susannah T Bellows[1], Deborah Marks[1,4], Patricia Howlin[5,6] & Nicole Rinehart[1].

Affiliations:

1. Deakin University, Geelong, Victoria, Australia
2. Murdoch Children's Research Institute, Parkville, Victoria, Australia.
3. Department of Paediatrics, University of Melbourne, Parkville, Victoria, Australia.
4. Royal Children's Hospital, Parkville, Victoria, Australia.
5. Brain and Mind Centre, University of Sydney, Camperdown, New South Wales, Australia.
6. Institute of Psychiatry, Psychology and Neuroscience, King's College London, London, UK.

Corresponding author

Dr Nicole Papadopoulos

Address: Level 4, Building BC. 211 Burwood Highway, Burwood. 3125, VIC.

Telephone: (03) 9244 5295

Email: nicole.papadopoulos@deakin.edu.au

For peer review only

Author's contributions

Nicole Rinehart, Nicole Papadopoulos, Emma Sciberras, Harriet Hiscock, Katrina Williams, Jane McGillivray, Pat Howlin and Cathrine Mihalopoulos conceived the study. Susannah Bellows, Deborah Marks and Lidia Engel contributed to the writing of the methods and intervention for the study. Nicole Papadopoulos and Susannah Bellows drafted the manuscript. Matthew Fuller-Tyszkiewicz contributed to the design and analytic components of the study. All authors contributed, read and approved the final manuscript.

Funding

This work was supported by the Australian National Health and Medical Research Council (NHMRC; project grant number APP1101989). Associate Professor Sciberras is funded by an NHMRC Career Development Fellowship 1110688 (2016-21) and a veski Inspiring Women's Fellowship. Harriet Hiscock is supported by an NHMRC Practitioner fellowship 1136222 (2018-2022). The MCRI is supported by the Victorian Government's Operational Infrastructure Support Program.

Competing interests

Professor Rinehart and Professor McGillivray received funding from the Moose Foundation and the Australian Football League to conduct research in the field of neurodevelopmental disorders and inclusion. Professor Rinehart, Professor McGillivray and Dr Papadopoulos also received funding from the Ferrero Group Australia as part of its Kinder + Sport pillar of Corporate Social Responsibility initiatives to promote active lifestyles among young people. Professor Rinehart has previously received speaker honorarium from Novartis (2002), Pfzier (2006) and Nutricia (2007) and is a Director of the Amaze Board (Autism Victoria). None of the companies or organisational bodies listed above had a role in this research including the

For peer review only

collection, analysis, and interpretation of data; in writing of the manuscript; and /or in the decision to submit the article for publication.

Exclusive license

I, the Submitting Author has the right to grant and does grant on behalf of all authors of the Work (as defined in the below author licence), an exclusive licence and/or a non-exclusive licence for contributions from authors who are: i) UK Crown employees; ii) where BMJ has agreed a CC-BY licence shall apply, and/or iii) in accordance with the terms applicable for US Federal Government officers or employees acting as part of their official duties; on a worldwide, perpetual, irrevocable, royalty-free basis to BMJ Publishing Group Ltd ("BMJ") its licensees and where the relevant Journal is co-owned by BMJ to the co-owners of the Journal, to publish the Work in BMJ Open and any other BMJ products and to exploit all rights, as set out in our licence.

The Submitting Author accepts and understands that any supply made under these terms is made by BMJ to the Submitting Author unless you are acting as an employee on behalf of your employer or a postgraduate student of an affiliated institution which is paying any applicable article publishing charge ("APC") for Open Access articles. Where the Submitting Author wishes to make the Work available on an Open Access basis (and intends to pay the relevant APC), the terms of reuse of such Open Access shall be governed by a Creative Commons licence – details of these licences and which Creative Commons licence will apply to this Work are set out in our licence referred to above.

Word count: 3961/4000

Keywords: Autism Spectrum Disorder; ASD; Sleep; Behavioural Sleep Intervention; RCT

For peer review only

ABSTRACT

Introduction: Sleep problems are a persistent feature of children with Autism Spectrum Disorder (ASD) with up to 86% of children experiencing sleep difficulties. In addition, sleep problems have been found to have a pervasive impact on a child's socio-emotional functioning as well as impacting on parents psychological functioning. The *Sleeping Sound ASD* project aims to evaluate the efficacy of a brief behavioural sleep intervention in reducing ASD children's sleep problems. We are also interested in the interventions impact on child and family functioning. The current study will evaluate this intervention in a fully powered randomised controlled trial (RCT).

Methods & Analysis: The RCT consists of 234 children with a diagnosis of ASD aged between 5-13 years who experience moderate to severe sleep problems. Participants will be recruited from paediatrician clinics in Victoria, Australia and via social media. Participants who register their interest in the study will be screened for eligibility and then asked to complete a baseline survey of children's sleep problems and child and family functioning. Participants will then be randomised to the intervention group or treatment as usual group. The intervention consists of families attending two face to face sessions and a follow up phone call with a trained clinician. During the intervention, families are provided with individually tailored behavioural sleep strategies to help manage their children's sleep problems. The primary outcome is children's sleep problems, as measured by the Children's Sleep Habits Questionnaire. Secondary outcomes include measures of children's social, behavioural, emotional, cognitive and quality of life and parent measures of parenting stress and mental health. The cost-effectiveness of the intervention will also be evaluated.

Ethics and dissemination: The findings from this study will be published in peer reviewed journals and disseminated at leading national and international conferences as well via social media.

For peer review only

Registration Details: ISRCTN14077107 (3/03/2017)

STRENGTH AND LIMITATIONS

- This sleep intervention consists of a brief behavioural intervention thus increasing the chances of the intervention being translated into clinical practice.
- This study is the largest randomised control trial to date on the efficacy of the Sleeping Sound program for children with ASD.
- Lack of objective assessments of sleep and exclusion of children with ASD who also have an intellectual disability are limitations of the study.

For peer review only

INTRODUCTION

Background

Autism Spectrum Disorder (ASD) is a neurodevelopmental disorder characterised by severe deficits in social, communicative and restricted and repetitive behaviours ,[1]. In 2015, it was estimated that ASD affected approximately 0.7% of the Australian population, which equates to approximately 164,000 Australians ,[2]. A diagnosis of ASD is associated with major child, familial and societal costs, with the economic burden in Australia estimated to be between $8.1 and $11.2 billion annually ,[3]. Psychiatric comorbidity is common in individuals with ASD, with approximately 70% meeting the criteria for another mental disorder ,[1], including anxiety (~84%) ,[4,5] and Attention-Deficit/Hyperactivity Disorder (ADHD; ~28 to 70%) ,[6,7]. Sleep problems are also very common in children with ASD, and cause an additional burden on functioning for children and their families. However, large rigorous randomised-controlled trials (RCTs) that report on the efficacy of treating sleep problems in this population are lacking.

Sleep problems in children with ASD - prevalence, aetiology and burden

It is estimated that between 73-86% of children with ASD experience sleep problems ,[8,9], with sleep onset and maintenance issues being particularly common (e.g., long sleep onset latency, waking after sleep onset),[9-12]. Other common sleep problems include resistance going to bed (bedtime resistance), daytime sleepiness and short sleep duration ,[9,12]. Children with ASD will also often experience more than one sleep problem concurrently ,[9]. Furthermore, sleep problems in ASD have been shown to be more persistent compared to typically developing children ,[13]. There are some indications that sleep problems are also more severe among children with ASD who have an intellectual disability, although this is currently unconfirmed ,[11,14-17].

For peer review only - http://bmjopen.bmj.com/site/about/guidelines.xhtml

The aetiology of sleep problems in children with ASD is unclear, although various biopsychosocial factors have been proposed. Biological factors include abnormalities in melatonin secretion ,[18]. Social learning problems may also contribute to risk, given that children with ASD often have difficulty recognizing contextual cues, such as 'sleep cues' that are vital in establishing healthy sleep-wake cycles ,[18]. Co-occurring psychological factors such as anxiety and depression as well as family factors may also influence sleep problems ,[19,20].

Several studies have linked sleep problems with associated psychopathology in ASD. In a large study conducted by Goldman, et al. 2011,[21] (*n* = 1,784), children with ASD who were poor sleepers were found to have more behavioural problems, including problems with attention and social interactions compared to good sleepers (77.7% vs 56.1% and 75.4% vs 53.9% respectively). Another study demonstrated that children with ASD who were poor sleepers experienced significantly more affective problems and reciprocal social interaction problems than children with ASD who were good sleepers ,[22]. Greater overall sleep problems have also been found to be associated with poorer child physical and psychosocial health related quality of life ,[23] and cognitive and motor functioning ,[16] as well as having a pervasive impact on the family, including elevated risk for parental stress and poor parental mental health ,[15,24-26].

Treating sleep problems in children with ASD

Melatonin is commonly prescribed to treat sleep problems in ASD ,[27]. Despite this, behavioural sleep interventions are recommended before considering medication. Gringras, et al. 2012,[28] conducted one of the largest melatonin sleep trials involving 146 children with neurodevelopmental disabilities (including ASD). To be eligible for the study children who

For peer review only

registered were provided with advice in the form of a booklet on previously trialled sleep hygiene and behavioural sleep strategies for a one-month period before being randomised for melatonin treatment. Following the administration of sleep hygiene and behavioural sleep strategies, 35% of the sample no longer met the inclusion criteria for the trial. Although this study did not solely focus on children with ASD, the results indicate the potential of a brief behavioural sleep intervention as a first-line sleep treatment for children with ASD.

Although there is promising evidence to support the use of behavioural sleep interventions for children with ASD, few studies have examined their efficacy. A recent systematic review of behavioural sleep interventions to treat insomnia in children with neurodevelopmental disorders identified 19 ASD studies, however, only three studies employed an RCT design ,[29]. The findings of the three studies were mixed. Adkins, et al. 2012,[30] (*n*=36) found no effect of a standardized information pamphlet on insomnia compared to a control (no intervention) group, suggesting that psychoeducation alone is insufficient to produce behavioural change and that specific/tailored examples may be more useful for parents when addressing child sleep problems.

Johnson, et al. 2013,[31] undertook a pilot RCT (*n*=40) comparing a five-session individualized parent behavioural sleep training program with a control group (parent psychoeducation autism program without a behavioural sleep focus). The behavioural sleep training program involved parents receiving information about how to treat common behavioural sleep problems their child may be experiencing as well as ways of addressing future sleep issues that are typical in ASD. The program comprised a wide range of resources in order to equip families with behavioural strategies to address a range of sleep disturbances often observed in ASD children. Families allocated to the behavioural sleep training program reported significant improvements in sleep problems (effects small to medium) as measured by the composite sleep index of the modified version of the Simonds and Parraga sleep

For peer review only

questionnaire ,[32,33]. Benefits were not identified using more objective measures (e.g. actigraphy). The study also did not report on any improvement in child and parent outcomes, despite parent satisfaction of the manualized program being high.

Malow, et al. 2014,[34] assessed the efficacy of a parent sleep education program on sleep onset delay in children with ASD (*n*=80), conducted in individual versus group (2-4 people) settings. Both delivery methods were effective in reducing sleep onset delay, with large effect. Improvement was also seen in bedtime resistance and sleep duration with medium-to-large effect, and sleep anxiety and night waking with small-to-medium effect. Improvements in child and family functioning were also reported, including increased child quality of life and parenting sense of competence, each with small-to-medium effect. However, the study lacked a treatment as usual control group.

More recently, Hiscock, et al. 2015,[35] has shown that a brief two session parent/child intervention *(Sleeping Sound)* that is tailored to the child and includes behavioural strategies targeted to the child's specific sleep problems was highly efficacious in reducing behavioural sleep problems in children with ADHD when compared to a treatment as usual control group. Based on this, we examined the preliminary efficacy of the *Sleeping Sound* program, in 61 children with comorbid ASD-ADHD ,[36]. In comparison to children with ASD-ADHD receiving treatment as usual (*n*=33), children with ASD-ADHD (*n*=28) who received the *Sleeping Sound* intervention showed large improvements in child sleep problems as measured by parent report of reduced scores on the Children's Sleep Habit Questionnaire ,[CSHQ, 37] at 3-months post-randomization, with medium-to-large effect, and 6-months post-randomization, with medium effect. The intervention group also showed greater improvements in psychosocial functioning at 3- and 6-month follow up, with small-to-medium and medium effect respectively, and were described as showing improved emotional

For peer review only

functioning at 3-months by parent report, with medium-to-large effect and teacher report, with medium effect.

It appears that a tailored behavioural sleep intervention may improve sleep in children with ASD, however, few rigorous RCTs have evaluated the efficacy of such intervention programs. Further, it is unclear whether behavioural sleep interventions for children with ASD leads to improvements in other domains of functioning such as behavioural and emotional problems, social communication, parental stress and parent mental health. Given our pilot findings showing that a tailored brief behavioural sleep intervention for children with ASD-ADHD improves not only sleep but emotional, behavioural and social functioning, we have selected this intervention to test in a fully powered RCT.

Aims and hypotheses

This study aims to examine the efficacy of a brief behavioural sleep intervention *(Sleeping Sound*) in treating sleep problems in primary school children aged 5-13 years with ASD. We aim to determine whether the brief sleep intervention delivered by study clinicians has the following outcomes:

Primary Outcome

1. Reductions in child sleep problem severity including reductions in initiating and maintaining sleep measured using the CSHQ at 3-months post randomisation.

Secondary Outcomes

2. Improvements in child and primary caregiver functioning at 3- and 6-months post randomisation.

3. Presents a cost-effective intervention when compared with Treatment as Usual (TAU) over the period of the study.

For peer review only

We hypothesize that compared with a TAU comparison group, children with ASD who receive the *Sleeping Sound* intervention will show greater improvements at 3-months post randomisation (post intervention; primary outcome time point) and 6-months post randomisation in the following areas:

1. Reductions in overall child sleep problem severity including reductions in total sleep disturbance, and problems initiating and maintaining sleep;
2. a. Improved daytime sleepiness by teacher report, improvements in sleep hygiene and decreases in the prevalence of child sleep problems from moderate/severe to no/mild by parent report;

   b. Decreased emotional and behavioural problems as reported by parents and teachers;

   c. Decreased social-communicative symptoms reported by parents and teachers;

   d. Increased cognitive performance, academic achievement and school attendance;

   e. Increased child quality of life on parent report;

   f. Decreased parent stress and mental health symptoms; and

   g. Increased parent quality of life and work attendance.

We also hypothesize that the behavioural sleep intervention will be a cost-effective treatment when compared with TAU from a societal and health care perspective, by comparing the costs and benefits (expressed in quality-adjusted life years (QALYs)) between the two arms of the trial over the period of the study (further details are provided below).

METHODS AND ANALYSIS

Overall study design

The *Sleeping Sound* ASD study is an RCT where participants are allocated either to the sleep intervention group or a treatment as usual group. The project runs from July 2016 to the

end of 2019 where all stages of the project from recruitment to follow up data collection at 6 months post-randomisation are executed.

Participants

Participants include families of children aged 5 to 12 years or 13 years attending either a kindergarten or school in Victoria at the time of recruitment. All participants have a paediatrician confirmed multidisciplinary diagnosis of ASD and a parent reported moderate to severe sleep problem of at least one of the following sleep problems: chronic insomnia or delayed sleep-wake phase as defined by the International Classification for Sleep Disorders – Third Edition ,[38].

Patient Involvement

The intervention used in this study is based on the ADHD *Sleeping Sound* Trial ,[35] where feedback of the acceptability and feasibility of the study for participants was collected. We also conducted a pilot study ,[36] of the *Sleeping Sound* intervention for children with ADHD-ASD and there were no reported concerns from participants. In this trial we will be collecting feedback data about the program from parents.

Recruitment: stage 1

Recruitment through Victorian paediatric clinics. Victorian paediatricians identify potential participants that meet the study inclusion criteria whom they have seen in the last 12 months and invite them to participate in the study by sending them a study invitation letter. Study advertisements are also displayed in the waiting areas of clinical services, and disseminated widely through research, clinical and community-based networks available to investigators.

For peer review only

Recruitment: stage 2

The research team telephones all primary caregivers who register interest in the study to assess inclusion/exclusion criteria (approximately 25-minute phone call). Eligible families are provided with an information sheet, consent form and baseline survey by email or post. The child's primary caregiver is asked to complete the survey and consent form. Once caregivers provide written consent, they are asked to provide diagnostic and cognitive assessment reports where available. If optional consent is provided to contact the child's school teacher, a link to an online survey is sent to the child's nominated teacher. Families are sent a separate hardcopy consent form for researchers to access the child's Medicare and Pharmaceutical Benefits Scheme (PBS) data for cost-effectiveness analysis. Medicare collects information on doctors' visits and the associated costs (including out-of-pocket costs). PBS collects information on the prescription medications which have been filled at pharmacies.

Please see Table 1 below for study inclusion and exclusion criteria. Participants are required to meet all inclusion criteria. Children currently prescribed melatonin as well as any other medications (SSRIs, etc.) are included in the trial if they continue to meet the eligibility criteria. Information regarding medication is collected as part of the parent survey.

(Insert Table 1 here)

Sample size

In our pilot study ,[36], we found a moderate-to-large effect of the intervention on sleep (effect size: 0.7). However, since effect sizes from small studies may not be replicated in larger studies, we conservatively estimate a moderate intervention effect size of 0.5 (equivalent to a 7% improvement in CSHQ scores in the intervention group relative to TAU).

For peer review only

The sample size calculation was performed for alpha = .05 (two-tailed), power = 80%, ICC = .08 for the within recruitment centre clustering (average cluster size = 4, SD = 3), and ICC = .20 for the within person clustering. Results showed that we will need to analyse results of 188 individuals (94 in each group). Subsequent Monte Carlo simulation showed that a sample of 188 observations would have sufficient precision (<10% bias for model coefficients and <5% bias for standard errors) and power (80%+) to estimate pertinent parameters in a three-level mixed effects linear regression model. Allowing for 20% attrition over time, we will randomize a minimum of 234 children (117 in each group) to ensure sufficient power post intervention (primary outcome timepoint 3-months post randomisation).

Randomisation

Participants are randomised to either the intervention or TAU comparison group by an independent research assistant once consent forms and baseline surveys have been completed. Treatment allocation is determined sequentially, according to a computer-generated block randomisation sequence, with blocks of randomly varying size developed by an independent researcher. Given the relatively fewer diagnoses of ASD among girls ,[39], randomisation is stratified by gender to ensure equal representation of girls, boys and other genders (i.e. transgender, intersex) in both study arms. Siblings were eligible to participate in the study provided they each met all inclusion criteria. Families with siblings enrolled in the study are randomised in sequence based on the return of the primary caregiver baseline surveys, with all subsequent siblings allocated to the same treatment group. All families are sent a letter informing them of their group allocation, and are not able to enrol further siblings once notified of group allocation. Intervention families are then called to book the intervention sessions.

For peer review only

The TAU comparison group will receive standard services and support currently available in the community and complete the same study assessment follow up. If the parent consented to teacher participation, we approach schools for consent to contact the child's teacher, and mail them a baseline survey to complete.

(Insert Figure 1 here)

For peer review only

Intervention group

Delivery of the sleep intervention. The behavioural sleep intervention is delivered by study-employed clinicians (paediatrician or psychologist experienced in working with children with ASD), and consists of two 50-minute face-to-face consultations and one follow up phone call at two-week intervals. The content of the original *Sleeping Sound* ADHD intervention ,[35] has been adapted to children with ASD to include specific visual aids and social stories to assist in the delivery of the sleep intervention for this population ,[40].

Study clinicians are trained by the team who developed the *Sleeping Sound* intervention, use a detailed program manual and meet bimonthly with supervisors to ensure fidelity to the treatment protocol. Sessions are held in rooms at one of our study sites (Deakin University or Royal Children's Hospital), or the treating paediatrician's consulting rooms.

Behavioural sleep interventions are individually tailored for the child and the family. The strategies which suit the family situation and learning style of the child with ASD are given emphasis. Delayed sleep phase and prolonged night time waking are treated with bedtime fading (i.e., child's bedtime is temporarily set later and gradually brought forward, as well as early morning light exposure). Need for parental presence at sleep time is managed for sleep onset association using either a 'checking' or 'camping out' method. Camping out

involves parents gradually withdrawing themselves from the child's bedroom over a period of time. Parent management strategies are used for limit-setting difficulties, and can include use of a 'bedtime pass', whereby the child can only leave the bedroom one time before sleep. Primary insomnia is managed using relaxation training and restricting time in bed (i.e. bedtime fading or getting out of bed and doing a relaxing activity if the child cannot fall asleep). In addition to relaxation training, examples of some strategies used to manage night time anxiety include discussing fears during the day rather than just before bedtime, encouraging the child to stay in bed and rewarding brave behaviour. Visual strategies, such as social scripts and visual timetables are used to reinforce learning.

Session One focuses on an assessment of the child's sleep problems, psychoeducation about normal sleep and sleep cycles, sleep hygiene, and a tailored plan specific to the sleep diagnosis of the child. Families receive written handouts, summarising session content and complete a written management plan with the clinician. A standardised consultation record is kept for all children including the presenting sleep problem(s), possible contributors to the problem (e.g., TV in bedroom), medication use, comorbidities, and bedtime routines. Clinicians record the duration of each consultation, sleep problem diagnoses, family sleep management goals, handouts given to parents, and management options selected.

Session Two is held two weeks later to review the sleep diary, reinforce strategies, trouble-shoot and monitor progress.

Follow up Phone Call conducted two weeks after the second visit to answer any further caregiver questions, reinforce strategies, trouble shoot and monitor progress.

Measures

Study measures are summarised in Table 2 below. Additional information collected at

For peer review only

baseline included family and child demographics such as socio-economic status (SES) and medical data (i.e., medication, diagnosed comorbidities etc.).

We have chosen the CSHQ ,[37] as our primary outcome measure as this is a widely-used and validated measure of behavioural sleep problems in children and was used in our pilot *Sleeping Sound* with ASD study ,[36]. It has been used to examine sleep behaviour in children with ASD and correlates highly with the objective measure of actigraphy ,[41].

Given that it is impossible to maintain participant blinding in a trial of this nature, we have carefully chosen some outcome measures that can be assessed in a blinded fashion. For example, we will include a blinded teacher-reported measure of sleep, behavioural, social and motor functioning. We will not inform teachers of the child's group status and will ask parents not to inform teachers. We will also include blinded objective child measures of executive and school-based functioning.

(Insert Table 2 here)

Data analysis

Primary analysis will be intent-to-treat and will compare outcomes of children in the control and intervention groups at the 3-months post randomisation timepoint. Secondary analyses will be undertaken at 3-months and 6-months post randomisation timepoints. Children in intervention and control groups will be compared on baseline prognostic factors and, if imbalances between the groups are identified, the relevant factor(s) will be included into analyses as a covariate. Differences in outcomes of treatment and intervention groups will be assessed with linear mixed models, with individuals clustered within recruitment centres and assessment times clustered within individuals. If imbalances between the groups are identified, the relevant factor(s) (i.e. child gender, age, socioeconomic status, parent

For peer review only

education, parent mental health and sleep medication use) will be included into analyses as a covariate. Recruitment centre/child's paediatrician and individual will be modelled as random effects and the remaining variables will be handled as fixed effects. To account for missing data, the mixed models will be fitted using conditional maximum likelihood. As this approach makes an untestable assumption that data are missing at random, i.e., missingness cannot be predicted from the variables included in the model, we will perform sensitivity analyses to explore the effect of departure from this assumption.

Economic evaluation

An important secondary aim of the project will be to determine the cost-effectiveness of this intervention compared to TAU. The inclusion of the CHU9D ,[42] will allow calculation of quality-adjusted life years (QALYs) as part of a cost-utility analysis, which will be undertaken along with a cost-consequence analysis, whereby the differences in costs between the intervention group and the TAU group will be compared with the differences for the full suite of study outcomes. This approach has been found to be useful to decision-makers ,[43]. The economic analysis will be conducted from a societal perspective, since interventions targeting children with ASD are likely to have benefits and costs beyond the narrow health sector (e.g., ASD intervention services and productivity impacts for carers). A secondary analysis from a health care perspective will also be undertaken. The evaluation will first measure and value any change to the use of health care resources over the period of the study between the two arms of the trial and then compare any additional costs to the additional outcomes achieved. Costs of the intervention will be measured using the financial records of the study team. Other costs incurred by children and their families will be measured using a resource use questionnaire, which will be adapted for use in this particular context. Components of the resource use questionnaire comprise the child's use of health care

For peer review only

services, including: (i) inpatient services and emergency services, (ii) health professional visits, (iii) medication, and (iv) other resources used. The questionnaire also collects data on out-of-pocket and travel costs, as well as a child's school attendance and carers' productivity. Participants will also be asked for their permission to access both their Medicare Benefits Scheme and PBS data for a time period of 3 months prior to baseline up until the last follow up assessment. These data are not subject to recall bias and provide complete resource use costs (and out of pocket costs) for all Medicare and PBS reimbursed health care services. Non-Medicare and non-PBS funded services (e.g., state-funded ASD services) will be captured in the resource use questionnaire. Standard economic evaluation techniques, including incremental analysis of mean differences and bootstrapping to determine confidence intervals, will be used in the economic evaluation ,[44].

ETHICS AND DISSEMINATION

Sleep problems are a common burden associated with ASD. The proposed RCT of the *Sleeping Sound* intervention in ASD children with sleep problems will result in a significant advance in research on the treatment of sleep problems. The study is well-justified with reference to the demonstrated success of this tailored sleep intervention in ADHD ,[35], our strong pilot data ,[36], and the potential advantages of the intervention in alleviating social-, emotional and academic problems connected to the sleep disorder in the children themselves and their parents. If the intervention is found to be effective in this sample of children who are of borderline/average intellectual ability we will subsequently be able to explore whether the intervention is successful for children with ASD who also have intellectual disability. Treatment recommendations for treating sleep problems in children with ASD have been released ,[45]. The Sleep Committee of the Autism Treatment Network ,[46] recommend the use of behavioural intervention as first line therapy. The Autism UK NICE clinical guidelines

For peer review only

(#170) for sleep management recommend development of individualised behavioural intervention which includes specialist monitoring, and use of a sleep plan as a central concept ,[47] on sleep management. To date, the treatment of sleep disturbance in children with ASD is based on expert opinion and consensus practise, informed by limited evidence. Results from this study will inform evidence based clinical practice and guide appropriate selection and tailoring of behavioural strategies to address specific sleep problems. We aim to publish our findings in peer-reviewed journals and present our research at international conferences and community organisations. The study has been granted ethics approval by the Royal Children's Hospital (#36154), Deakin University (#2017-130), the Victorian Department of Education and Early Childhood Development (#2016_003134) and the Catholic Education Office (Melbourne #0501) Human Research Ethics Committees.

For peer review only

Table 1

Study Inclusion and Exclusion Criteria.

Inclusion Criteria	
	Multidisciplinary diagnosis of DSM-IV ,[48] Autistic Disorder, Asperger's Disorder or DSM-5 ,[1] ASD, as indicated by paediatrician or a confirmed diagnosis as cited in a clinical report.
	Aged 5-12 years, or 13 years and attending a Victorian kindergarten or school at the time of recruitment.
	Child meets clinical ASD symptomology based on a clinical cut off score of ≥ 11 on the Social Communication Questionnaire Lifetime form (40 item questionnaire measuring ASD symptoms) ,[49].
	Child's sleep problem(s) are moderate or severe by primary caregiver report and have been a problem for them over the past 4 weeks ,[50].
	Meet diagnostic criteria for at least one of the following sleep problems as defined by the International Classification of Sleep disorders diagnostic criteria ,[38] chronic insomnia (including problems initiating and maintaining sleep, sleep-onset association, limit-setting, and anxiety-related insomnia), or delayed sleep-wake phase.
Exclusion Criteria	
	Diagnosed with an intellectual disability by parent report.
	Have any comorbid medical (e.g. nocturnal seizures, blindness) conditions that disturb regular sleep patterns, or genetic (e.g., Fragile X disorder) conditions associated with intellectual impairment based on parent report.
	Have suspected obstructive sleep apnoea (OSA). OSA is assessed using 3 OSA items from the CSHQ ,[37]. This scale can help identify children who may suffer from OSA. If a parent endorses items indicative of OSA, the child is contacted by the study paediatrician for further assessment. If OSA is suspected, the child is excluded from the research study and referred to appropriate clinical services. After approximately six months the family is followed up to see whether they are eligible to participate in the study (i.e. if OSA symptoms have resolved and they have ongoing behaviour sleep problems).
	Parents with insufficient English language proficiency to provide informed consent, complete study materials and/or participate in the intervention treatment program.

For peer review only

Table 2
Summary of measures included in the study

Construct	Measure	Source	B	3	6
**Child outcomes**					
Sleep	Primary caregiver report of child sleep problem i.e. none, mild, moderate or severe ,[50].	Parent	●	●	●
Sleep	Children's Sleep Habits Questionnaire (CSHQ). 33-item validated measure of sleep that can distinguish clinical from community samples ,[37].	Parent	●	●	●
Sleep	Teacher Daytime Sleepiness Questionnaire. 10 item validated scale of daytime sleepiness at school ,[51].	Teacher	●	●	●
Sleep	Sleep Hygiene scale. 7-item study developed measure adapted from the bedroom routines scale.	Parent	●	●	●
Social-communicative functioning	Social Communication Questionnaire. Current form. 40-item measure of ASD symptoms in past 3 months ,[49].	Parent	●	●	●
Emotional and behavioural disturbance	Developmental Behavioural Checklist (DBC). 96-item measure of emotional and behavioural disturbance in children ,[52].	Parent	●		●
Behavioural and social functioning	Strengths and Difficulties Questionnaire. 25 items assessing the following subscales: hyperactivity/inattention, conduct problems, emotional symptoms, peer relationship problems, and prosocial behaviour ,[53].	Parent & Teacher	●	●	●
Quality of life	Child Health Utility 9D (CHU9D). 9 item proxy measure of child quality of life ,[42].	Parent	●	●	●
School attendance	School attendance over the preceding 3 months.	Parent	●	●	●
Academic functioning	Wide Range Achievement Test 4 (WRAT). Spelling and math computation subtests to assess academic functioning ,[54].	Child			●
Academic functioning	Permission to access NAPLAN results (Years 3, 5, 7). NAPLAN is a national assessment of literacy and numeracy Australian children complete in Grades 3, 5, 7 and 9.	Linkage	●	●	●
Cognitive functioning	NIH Toolbox – Cognitive Domain to assess cognitive functioning ,[55] .	Child			●
**Parent outcomes**					
Mental health	Kessler 10 (K10). A 10-item validated measure of adult psychological distress ,[56].	Parent	●	●	●
Parenting stress	Parenting Stress Index 4SF (PSI-4SF). 36 item measure of parenting stress ,[57].	Parent	●	●	●
Quality of life	AQoL4D. 12 item measure of parent quality of life ,[58].	Parent	●	●	●
**Economic outcomes**					
Resource use	Family reported service use over preceding 3 months	Parent	●	●	●
Health service utilisation	Permission to access both Medicare and Pharmaceutical Benefits Scheme (PBS) data for a time period of 3 months prior to baseline up until the last follow-up assessment.	Linkage	●	●	●

● measure utilised at indicated timepoint.
B, baseline; 3, 3 months; 6, 6 months

For peer review only

References

1. American Psychiatric Association. Diagnostic and Statistical Manual of Mental Disorders. 5th ed. Arlington, VA: American Psychiatric Publishing 2013.

2. Australian Institute of Health and Welfare. Autism in Australia 2017 [Available from: https://www.aihw.gov.au/reports/disability/autism-in-australia/contents/autism accessed 22nd August 2018.

3. Synergies Economic Consulting. Economic costs of autism spectrum disorder in Australia: updated study, 2011.

4. Muris P, Steerneman P, Merckelbach H, et al. Comorbid anxiety symptoms in children with pervasive developmental disorders. *J Anxiety Disord* 1998;12(4):387-93. doi: 10.1016/S0887-6185(98)00022-X

5. White SW, Oswald D, Ollendick T, et al. Anxiety in children and adolescents with autism spectrum disorders. *Clin Psychol Rev* 2009;29(3):216-29. doi: 10.1016/j.cpr.2009.01.003

6. Gadow KD, Devincent CJ, Pomeroy J, et al. Comparison of DSM-IV symptoms in elementary school-age children with PDD versus clinic and community samples. *Autism* 2005;9(4):392-415. doi: 10.1177/1362361305056079

7. Simonoff E, Pickles A, Charman T, et al. Psychiatric disorders in children with autism spectrum disorders: prevalence, comorbidity, and associated factors in a population-derived sample. *Journal of the American Academy of Child & Adolescent Psychiatry* 2008;47(8):921-29. doi: 10.1097/CHI.0b013e318179964f

8. Polimeni MA, Richdale AL, Francis AJP. A survey of sleep problems in autism, asperger's disorder and typically developing children. *Journal of Intellectual Disability Research* 2005;49(4):260-68. doi: 10.1111/j.1365-2788.2005.00642.x

For peer review only

9. Liu X, Hubbard JA, Fabes RA, et al. Sleep disturbances and correlates of children with autism spectrum disorders. *Child Psychiatry and Human Development* 2006;37:179-91. doi: 10.1007/s10578-006-0028-3

10. Richdale AL, Prior MR. The sleep-wake rhythm in children with autism. *European Journal of Adolescent Psychiatry* 1995;4:175-86. doi: 10.1007/BF01980456

11. Krakowiak P, Goodlin-Jones B, Hertz-Picciotto I, et al. Sleep problems in children with autism spectrum disorders, developmental delays, and typical development: a population-based study. *J Sleep Res* 2008;17(2):197-206. doi: 10.1111/j.1365-2869.2008.00650.x

12. Cohen S, Conduit R, Lockley SW, et al. The relationship between sleep and behavior in autism spectrum disorder (ASD): a review. *J Neurodev Disord* 2014;6(44):1-10. doi: 10.1186/1866-1955-6-44

13. Sivertsen B, Posserud M-B, Gillberg C, et al. Sleep problems in children with autism spectrum problems: a longitudinal population-based study. *Autism* 2012;16(2):139-50. doi: 10.1177/1362361311404255

14. Mayes SD, Calhoun SL. Variables related to sleep problems in children with autism. *Research in Autism Spectrum Disorders* 2009;3(4):931-41. doi: 10.1016/j.rasd.2009.04.002

15. Doo S, Wing YK. Sleep problems of children with pervasive developmental disorders: correlation with parental stress. *Developmental Medicine and Child Neurology* 2006;48(8):650-55. doi: 10.1017/S001216220600137X

16. Taylor MA, Schreck KA, Mulick JA. Sleep disruption as a correlate to cognitive and adaptive behavior problems in autism spectrum disorders. *Res Dev Disabil* 2012;33(5):1408-17. doi: 10.1016/j.ridd.2012.03.013

For peer review only

17. Williams PG, Sears LL, Allard A. Sleep problems in children with autism. *J Sleep Res* 2004;13:265-68. doi: 10.1111/j.1365-2869.2004.00405.x

18. Richdale AL. Sleep disorders in autism and Asperger's syndrome. In: Stores G, Wiggs L, eds. Sleep Disturbances in Disorders of Development: Its Significance and Management. London: MacKeith Press 2001:181–91.

19. Richdale AL, Schreck KA. Sleep problems in autism spectrum disorders: prevalence, nature, & possible biopsychosocial aetiologies. *Sleep Med Rev* 2009;13(6):403-11. doi: 10.1016/j.smrv.2009.02.003

20. Leyfer OT, Folstein SE, Bacalman S, et al. Comorbid psychiatric disorders in children with autism: interview development and rates of disorders. *Journal of Autism and Developmental Disorders* 2006;36(7):849-61. doi: 10.1007/s10803-006-0123-0

21. Goldman SE, McGrew S, Johnson KP, et al. Sleep is associated with problem behaviors in children and adolescents with autism spectrum disorders. *Research in Autism Spectrum Disorders* 2011;5(3):1223-29. doi: 10.1016/j.rasd.2011.01.010

22. Malow BA, Marzec MS, McGrew SG, et al. Characterizing sleep in children with autism spectrum disorders: a multidimensional approach. *SLEEP* 2006;29(12):1563-71. doi: 10.1093/sleep/29.12.1563

23. Delahaye J, Kovacs E, Sikora D, et al. The relationship between health-related quality of life and sleep problems in children with autism spectrum disorders. *Research in Autism Spectrum Disorders* 2014;8(3):292-303. doi: 10.1016/j.rasd.2013.12.015

24. Hodge D, Hoffman CD, Sweeney DP, et al. Relationship between children's sleep and mental health in mothers of children with and without autism. *Journal of Autism and Developmental Disorders* 2013;43(4):956-63. doi: 10.1007/s10803-012-1639-0

For peer review only

25. Hoffman CD, Sweeney DP, Lopez-Wagner MC, et al. Children with autism: sleep problems and mothers' stress. *Focus on Autism and Other Developmental Disabilities* 2008;23(3):155-65. doi: 10.1177/1088357608316271

26. Johnson CR, Smith T, DeMand A, et al. Exploring sleep quality of young children with autism spectrum disorder and disruptive behaviors. *Sleep Med* 2018;44:61-66. doi: 10.1016/j.sleep.2018.01.008

27. Phillips L, Appleton RE. Systematic review of melatonin treatment in children with neurodevelopmental disabilities and sleep impairment. *Developmental Medicine and Child Neurology* 2004;46(11):771-75. doi: 10.1017/S001216220400132X

28. Gringras P, Gamble C, Jones AP, et al. Melatonin for sleep problems in children with neurodevelopmental disorders: randomised double masked placebo controlled trial. *BMJ* 2012;345:e6664. doi: 10.1136/bmj.e6664

29. Rigney G, Ali NS, Corkum P, et al. A systematic review to explore the feasibility of a behavioural sleep intervention for insomnia in children with neurodevelopmental disorders: a transdiagnostic approach. *Sleep Med Rev* 2018;41:244-54. doi: 10.1016/j.smrv.2018.03.008

30. Adkins KW, Molloy C, Weiss SK, et al. Effects of a standardized pamphlet on insomnia in children with autism spectrum disorders. *Pediatrics* 2012;130(Supplement 2):S139-S44. doi: 10.1542/peds.2012-0900K

31. Johnson CR, Turner KS, Foldes E, et al. Behavioral parent training to address sleep disturbances in young children with autism spectrum disorder: a pilot trial. *Sleep Med* 2013;14(10):995-1004. doi: 10.1016/j.sleep.2013.05.013

32. Wiggs L, Stores G. Behavioural treatment for sleep problems in children with severe learning disabilities and challenging daytime behaviour: effect on sleep patterns of

For peer review only

mother and child. *J Sleep Res* 1998;7(2):119-26. doi: 10.1046/j.1365-2869.1998.00107.x

33. Simonds JF, Parraga H. Prevalence of sleep disorders and sleep behaviors in children and adolescents. *Journal of the American Academy of Child & Adolescent Psychiatry* 1982;21(4):383-88. doi: 10.1016/S0002-7138(09)60942-0

34. Malow BA, Adkins KW, Reynolds A, et al. Parent-based sleep education for children with autism spectrum disorders. *Journal of Autism and Developmental Disorders* 2014;44(1):216-28. doi: 10.1007/s10803-013-1866-z

35. Hiscock H, Sciberras E, Mensah F, et al. Impact of a behavioral sleep intervention on ADHD symptoms, child sleep and parent health: a randomized controlled trial. *The BMJ* 2015;350:1-14. doi: 10.1136/bmj.h68

36. Papadopoulos N, Sciberras E, Hiscock H, et al. The efficacy of a brief behavioural sleep intervention in school-aged children with ADHD and comorbid autism spectrum disorder. *J Atten Disord* 2015:1-10. doi: 10.1177/1087054714568565

37. Owens JA, Spirito A, McGuinn M. The Children's Sleep Habits Questionnaire (CSHQ): psychometric properties of a survey instrument for school-aged children. *SLEEP* 2000;23(8):1043-51. doi: 10.1093/sleep/23.8.1d

38. American Academy of Sleep Medicine. International Classification of Sleep Disorders (ISCD-3). 3rd ed. Darian, IL: American Academy of Sleep Medicine 2014.

39. Kim YS, Leventhal BL, Koh Y-J, et al. Prevalence of autism spectrum disorders in a total population sample. *Am J Psychiatry* 2011;168(9):904-12. doi: 10.1176/appi.ajp.2011.10101532

40. Sivaratnam CSea. Brief report: assessment of the social-emotional profile in children with autism spectrum disorders using a novel comic strip task. *Journal of Autism and Developmental Disorders* 2012;42(11):2505-12. doi: 10.1007/s10803-012-1498-8

For peer review only

41. Souders MC, Mason TBA, Valladares O, et al. Sleep behaviors and sleep quality in children with autism spectrum disorders. *SLEEP* 2009;32(12):1566. doi: 10.1093/sleep/32.12.1566

42. Stevens K. Valuation of the Child Health Utility 9D Index. *PharmacoEconomics* 2012;30(8):729-47. doi: 10.2165/11599120-000000000-00000

43. Coast J. Is economic evaluation in touch with society's health values? *BMJ* 2004;329(7476):1233. doi: 10.1136/bmj.329.7476.1233

44. Glick HA, Doshi JA, Sonnad SS. Economic Evaluation in Clinical Trials. Oxford, UK: Oxford University Press 2007.

45. Abel E, Kim SY, Kellerman AM, et al. Recommendations for identifying sleep problems and treatment resources for children with autism spectrum disorder. *Behavioral Analysis Practice* 2017;10:261-69. doi: 10.1007/s40617-016-0158-4

46. Malow BA, Byars K, Johnson K, et al. A practice pathway for the identification, evaluation, and management of insomnia in children and adolescents with autism spectrum disorders. *Pediatrics* 2012;130(Supplement 2):S106-S24. doi: 10.1542/peds.2012-0900I

47. National Institute for Health and Care Excellence. Autism: the management and support of children and young people on the autism spectrum (Clinical guideline 170) 2013 [Available from: http://guidance.nice.org.uk/CG170.

48. American Psychiatric Association. Diagnostic and Statistical Manual of Mental Disorders. 4th ed. Washington, DC: Author 2000.

49. Rutter M, Bailey A, Lord C. The Social Communication Questionnaire. Los Angeles: Western Psychological Services 2003.

For peer review only

50. Hiscock H, Canterford L, Ukoumunne OC, et al. Adverse associations of sleep problems in Australian preschoolers: national population study. *Pediatrics* 2007;119(1):86-93. doi: 10.1542/peds.2006-1757

51. Owens JA, Spirito A, McGuinn M, et al. Sleep habits and sleep disturbance in elementary school-aged children. *Journal of Developmental and Behavioral Pediatrics* 2000;21(1):27-36. doi: 10.1097/00004703-200002000-00005

52. Einfield SL, Tonge BJ. Manual for the Developmental Behaviour Checklist: Primary Carer Version (DBC-P) and Teacher (DBC-T) Version. Melbourne, Australia: Monash University Centre for Developmental Psychiatry and Psychology 2002.

53. Goodman R. Psychometric properties of the strengths and difficulties questionnaire. *Journal of the American Academy of Child & Adolescent Psychiatry* 2001;40:1337-45. doi: 10.1097/00004583-200111000-00015

54. Wilkinson GS, Robertson GJ. WRAT 4: Wide Range Achievement Test; Professional Manual: Psychological Assessment Resources, Incorporated 2006.

55. Gershon RC, Cella D, Fox NA, et al. Assessment of neurological and behavioural function: the NIH Toolbox. *Lancet Neurol* 2010;9(2):138-39. doi: 10.1016/S1474-4422(09)70335-7

56. Kessler RC, Barker PR, Colpe LJ, et al. Screening for serious mental illness in the general population. *Archives of General Psychiatry* 2003;60(2):184-9. doi: 10.1001/archpsyc.60.2.184

57. Abidin RR. Parenting Stress Index. 4 ed. Lutx, FL: PAR 2012.

58. Richardson J, Hawthorne G. The Australian Quality of Life (AQoL) instrument: psychometric properties of the descriptive system and initial validation. *Australian Studies of Health Service Administration* 1998;85:315-42.

For peer review only

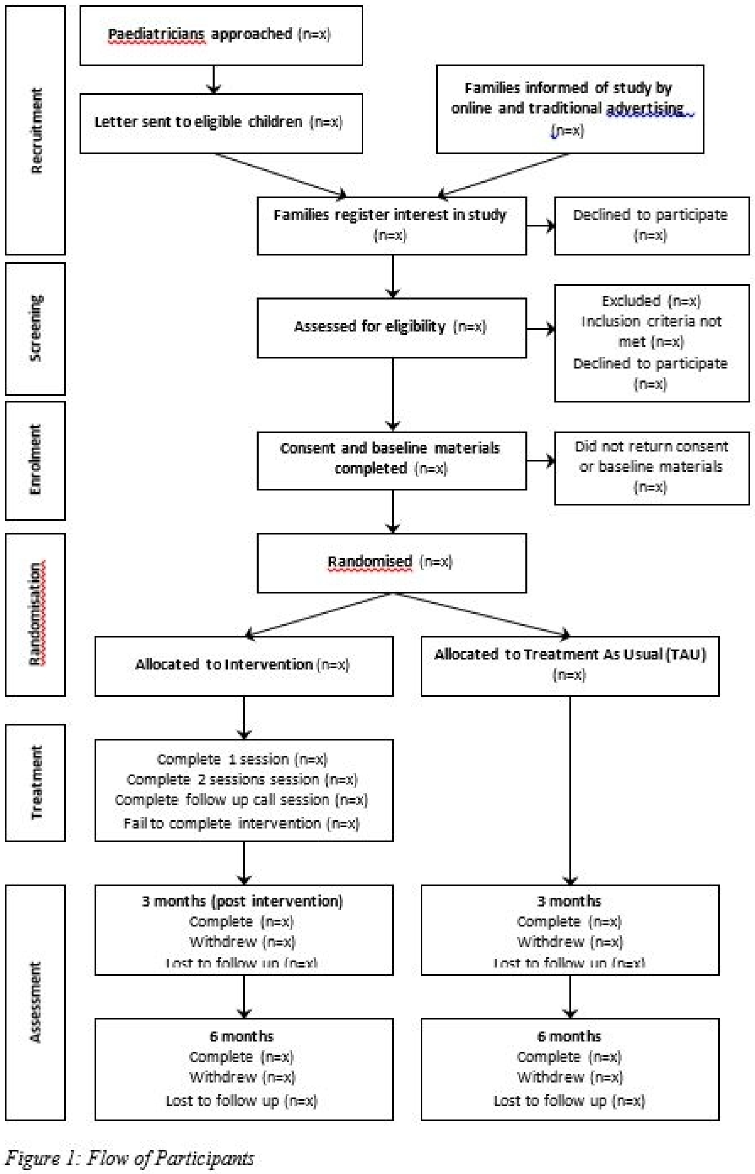

Figure 1:Flow of Participants

120x187mm (96 x 96 DPI)

BMJ Open

Sleeping Sound with Autism Spectrum Disorder (ASD): Study protocol for an efficacy randomized control trial of a tailored brief behavioural sleep intervention for autism spectrum disorder.

Journal:	*BMJ Open*
Manuscript ID	bmjopen-2019-029767.R1
Article Type:	Protocol
Date Submitted by the Author:	26-May-2019
Complete List of Authors:	Papadopoulos, Nicole; Deakin University<br>Sciberras, Emma; Deakin University; Murdoch Childrens Research Institute<br>Hiscock, Harriet; Murdoch Childrens Research Institute; The Royal Children's Hospital<br>Williams, Katrina; Murdoch Childrens Research Institute; University of Melbourne, Department of Paediatrics<br>McGillivray, Jane; Deakin University<br>Mihalopoulos, Cathrine; Deakin University<br>Engel, Lidia; Deakin University<br>Fuller-Tyszkiewicz, Matthew; Deakin University<br>Bellows, Susannah; Deakin University<br>Marks, Deborah; Deakin University; Royal Children's Hospital Melbourne<br>Howlin, Patricia; University of Sydney, Brain and Mind Centre; King`s College London, Institute of Psychiatry, Psychology and Neuroscience<br>Rinehart, Nicole; Deakin University
**Primary Subject Heading**:	Evidence based practice
Secondary Subject Heading:	Mental health
Keywords:	ASD, Autism Spectrum Disorder, Sleep, Behavioural sleep intervention, RCT

SCHOLARONE™
Manuscripts

Title: Sleeping Sound with Autism Spectrum Disorder (ASD): Study protocol for an efficacy randomized control trial of a tailored brief behavioural sleep intervention for autism spectrum disorder.

Nicole Papadopoulos[1], Emma Sciberras[1,2,3], Harriet Hiscock[2,4], Katrina Williams[2,3,4], Jane McGillivray[1], Cathrine Mihalopoulos[1], Lidia Engel[1], Matthew Fuller-Tyszkiewicz[1], Susannah T Bellows[1], Deborah Marks[1,4], Patricia Howlin[5,6] & Nicole Rinehart[1].

Affiliations:

1. Deakin University, Geelong, Victoria, Australia
2. Murdoch Children's Research Institute, Parkville, Victoria, Australia.
3. Department of Paediatrics, University of Melbourne, Parkville, Victoria, Australia.
4. Royal Children's Hospital, Parkville, Victoria, Australia.
5. Brain and Mind Centre, University of Sydney, Camperdown, New South Wales, Australia.
6. Institute of Psychiatry, Psychology and Neuroscience, King's College London, London, UK.

Corresponding author

Dr Nicole Papadopoulos

Address: Level 4, Building BC. 211 Burwood Highway, Burwood. 3125, VIC.

Telephone: (03) 9244 5295

Email: nicole.papadopoulos@deakin.edu.au

Word count: 5,201

Keywords: Autism Spectrum Disorder; ASD; Sleep; Behavioural Sleep Intervention; RCT

For peer review only

ABSTRACT

Introduction: Sleep problems are a characteristic feature of children with Autism Spectrum Disorder (ASD) with 40 to 80% of children experiencing sleep difficulties. In addition, sleep problems have been found to have a pervasive impact on a child's socio-emotional functioning as well as on parents' psychological functioning. The *Sleeping Sound ASD* project aims to evaluate the efficacy of a brief behavioural sleep intervention in reducing ASD children's sleep problems. We are also interested in the intervention's impact on child and family functioning. The current study will evaluate this intervention in a fully powered randomized controlled trial (RCT).

Methods & analysis: The RCT aims to recruit 234 children with a diagnosis of ASD, aged between 5-13 years, who experience moderate to severe sleep problems. Participants are recruited from paediatrician clinics in Victoria, Australia and via social media. Participants who register their interest in the study are screened for eligibility via phone and then asked to complete a baseline survey assessing children's sleep problems and child and family functioning online. Participants are then randomized to the intervention group or treatment as usual comparator group. The intervention consists of families attending two face to face sessions and a follow up phone call with a trained clinician. Given the nature of the intervention, participants are not blinded to the intervention. However, assessments of cognitive ability will be conducted by blind assessors; teacher reports of sleep, behavioural, social and motor skills measures will also be blind to treatment. During the intervention, families are provided with individually tailored behavioural sleep strategies to help manage their children's sleep problems. The primary outcome is children's sleep problems, as measured by the Children's Sleep Habits Questionnaire. Secondary outcomes include measures of children's social, behavioural, emotional, cognitive and quality of life and parent

For peer review only

measures of parenting stress and mental health. The cost-effectiveness of the intervention is also evaluated.

Ethics and dissemination: The findings from this study will be published in peer-reviewed journals and disseminated at leading national and international conferences as well via social media.

Registration details: ISRCTN registry - ISRCTN14077107 (3/03/2017)

Protocol version: Protocol amendment #9 (16/05/2019)

STRENGTHS AND LIMITATIONS

- This sleep intervention consists of a brief behavioural intervention thus increasing the chances of the intervention being translated into clinical practice.
- This study is the largest randomized control trial to date on the efficacy of the Sleeping Sound program for children with ASD.
- Limitations of the study include: lack of an objective sleep measure, inability to control families' use of additional sleep treatments post-trial enrolment and lack of gold standard ASD diagnostic tools (e.g. ADOS-2, ADI-R) to confirm ASD diagnosis.

For peer review only

Autism spectrum disorder

Autism spectrum disorder (ASD) is a neurodevelopmental disorder characterised by severe deficits in social, communication skills and restricted and repetitive patterns of behaviour.[1] In 2015, it was estimated that ASD affected approximately 0.7% of the Australian population, which equates to approximately 164,000 Australians.[2] A diagnosis of ASD is associated with major child, familial and societal costs, with the economic burden in Australia estimated to be between $8.1 and $11.2 billion annually.[3] Psychiatric comorbidity is common in individuals with ASD, with approximately 70% meeting the criteria for another mental disorder,[1] including anxiety (~84%),[4, 5] and attention-deficit/hyperactivity disorder (ADHD; ~28 to 70%).[6, 7] Sleep problems are also very common in children with ASD and cause an additional burden for children and their families. However, large rigorous randomized controlled trials (RCTs) that report on the efficacy of treating sleep problems in this population are lacking.[8]

Sleep problems in children with ASD - prevalence, aetiology and burden

It is estimated 40-80% of children with ASD experience sleep problems,[9] with sleep onset delay and night waking being particularly common.[9-12] Other common sleep problems include bedtime resistance, daytime sleepiness and short sleep duration.[9, 12] Children with ASD will also often experience more than one sleep problem concurrently.[12] Furthermore, sleep problems in children with ASD have been shown to be more persistent over time compared to sleep problems in typically developing children.[13] .

The aetiology of sleep problems in children with ASD is unclear, although various biopsychosocial factors have been proposed. Biological factors include abnormalities in melatonin secretion.[14] Social learning problems may also contribute given children with ASD often have difficulty recognizing contextual cues, such as 'sleep cues' that are vital in

For peer review only

establishing healthy sleep-wake cycles.[14] Co-occurring psychological factors such as anxiety and depression, as well as family factors, may also influence sleep problems.[15, 16]

Several studies have linked sleep problems with behavioural difficulties and psychopathology in ASD. In a large study conducted by Goldman et al.[17] (*n* = 1,784), children with ASD who were poor sleepers were found to have more behavioural problems, including problems with attention and social interactions compared to good sleepers (77.7% vs 56.1% and 75.4% vs 53.9% respectively). Sleep problems in children with ASD have also been associated with thinking difficulties;[18] increased communication and social deficits and higher rates of stereotypic behaviours;[19, 20] and with anxiety and mood disturbances.[21] In addition, overall severity of sleep problems has been found to be associated with poorer child physical and psychosocial health related quality of life,[22] and cognitive and motor functioning.[23]

Sleep problems have also been identified as having a pervasive impact on the family, including an elevated risk for parenting stress. For example, several studies have identified moderate to small associations between parenting stress and sleep problems in children with ASD.[24-28] Two studies have linked sleep problems in children with ASD with poor parent mental health, with one study finding a moderate association between child sleep problems and maternal mental health,[27] and another reporting a small-to-medium association between child sleep problems and parent depression.[29]

Treating sleep problems in children with ASD

Both behavioural and pharmacological interventions are commonly prescribed to treat sleep problems in ASD, however, we have much to learn about the efficacy of these treatment approaches.[8] For some sleep disorders, behavioural sleep interventions are recommended as the first line of treatment. Gringras et al.[30] conducted one of the largest melatonin sleep trials involving 146 children with neurodevelopmental disabilities (including

For peer review only

ASD). To be eligible for that study children who registered were provided with advice in the form of a booklet on previously trialled good sleep habits and behavioural sleep strategies for a one-month period before being randomized for melatonin treatment. Following the administration of good sleep habits and behavioural sleep strategies, 35% of the sample no longer met inclusion criteria for the trial. Although this study did not solely focus on children with ASD, the results indicate the potential of a brief behavioural sleep intervention for children with ASD.

Although there is promising evidence to support the use of behavioural sleep interventions for children with ASD, few studies have examined their efficacy.[8, 31] A recent systematic review of behavioural sleep interventions to treat insomnia in children with neurodevelopmental disorders identified 19 ASD studies, however, only three studies employed an RCT design.[32] The findings of the three studies were mixed. Adkins et al.[33] (*n* = 36) found no effect of a standardized information pamphlet on insomnia compared to a control (no intervention) group, suggesting that psychoeducation alone is insufficient to produce behavioural change and that specific/tailored examples may be more useful for parents when addressing child sleep problems.

Johnson et al.[34] undertook a pilot RCT (*n* = 40) comparing a five-session individualized parent behavioural sleep training program with a control group (parent psychoeducation autism program without a behavioural sleep focus). The behavioural sleep training program involved parents receiving information about how to treat behavioural sleep problems often observed in children with ASD, as well as ways of addressing future sleep issues that are typical in ASD. Families allocated to the behavioural sleep training program reported significant improvements in sleep problems (effects small to medium) as measured by the composite sleep index of the modified version of the Simonds and Parraga sleep questionnaire.[35, 36] However, no benefits were identified using more objective measures

For peer review only

(e.g. actigraphy) and the authors do not report on any improvement in child and parent outcomes, despite parent satisfaction of the manualized program being high.

Malow et al.[37] assessed the efficacy of a parent sleep education program on sleep onset delay in children with ASD (*n* = 80), conducted in individual versus group (2-4 people) settings. Both delivery methods were effective in reducing sleep onset delay, with large effect. Improvement was also seen in bedtime resistance and sleep duration with medium-to-large effect, and sleep anxiety and night waking with small-to-medium effect. Improvements in child and family functioning were also reported, including increased child quality of life and parental sense of competence, each with small-to-medium effect. However, the study lacked a treatment as usual (TAU) control group.

More recently, Hiscock et al.[38] showed that a brief two session parent/child intervention *(Sleeping Sound)* that is tailored to the child and includes behavioural strategies targeted at the child's specific sleep problems was highly efficacious in reducing behavioural sleep problems in children with ADHD when compared to a TAU control group. Based on this, we examined the preliminary efficacy of the *Sleeping Sound* program, in 61 children with comorbid ASD-ADHD.[39] In comparison to children with ASD-ADHD receiving TAU (*n* = 33), children with ASD-ADHD (*n* = 28) who received the *Sleeping Sound* intervention showed large improvements in child sleep problems as measured by parent report of reduced total scores on the Children's Sleep Habit Questionnaire (CSHQ),[40] at 3-months post-randomization, with medium-to-large effect, and 6-months post-randomization, with medium effect. The intervention group also showed greater improvements in psychosocial functioning at 3- and 6-month follow up, with small-to-medium and medium effect respectively, and were described as showing improved emotional functioning at 3-months by parent-report, with medium-to-large effect and teacher-report, with medium effect.

For peer review only

It appears that a tailored behavioural sleep intervention may improve sleep in children with ASD, however, few rigorous RCTs have been conducted.[8, 31] Further, it is unclear whether behavioural sleep interventions for children with ASD lead to improvements in other domains of functioning such as behavioural and emotional problems, social communication, parenting stress and parent mental health. Given that our pilot findings demonstrated that a tailored brief behavioural sleep intervention for children with ASD-ADHD can improve not only sleep but also emotional, behavioural and social functioning, we have selected this intervention to test in a fully powered RCT.

Aims and hypotheses

This study aims to examine the efficacy of a brief behavioural sleep intervention (*Sleeping Sound*) in treating sleep problems in primary school children aged 5-13 years with ASD compared to a TAU group. We have chosen a TAU control group for this trial to allow us to investigate the efficacy of the Sleeping Sound intervention for children with autism who may already be receiving community or clinical interventions.

We aim to assess whether the brief sleep intervention delivered by study clinicians is related to the following outcomes:

Primary Outcome

1. Reductions in overall child sleep problem severity and reductions in total sleep disturbances, problems initiating sleep and problems maintaining sleep at 3-months post randomization.

Secondary Outcomes

2. Improvements in child and primary parent functioning at 3- and 6- months post randomization.

We also aim to assess whether the brief sleep intervention is cost-effective when compared with TAU.

For peer review only

We hypothesize that compared with a TAU comparison group, children with ASD who receive the *Sleeping Sound* intervention will show greater improvements at 3-months post randomization (post intervention; primary outcome time point) and 6-months post randomization in the following areas:

1. Reduction in overall child sleep problem severity
2. a. Decreased daytime sleepiness by teacher report and improvements in sleep hygiene and sleep problem severity by parent report;

   b. Decreased emotional and behavioural problems reported by parents and teachers;

   c. Decreased social-communicative symptoms reported by parents and teachers;

   d. Increased cognitive performance, academic achievement and school attendance;

   e. Increased child quality of life reported by parents;

   f. Decreased parent stress and mental health symptoms; and

   g. Increased parent quality of life and work attendance.

We also hypothesize that the behavioural sleep intervention will be a cost-effective treatment when compared with TAU from a societal and healthcare perspective. Thus, we will compare the costs and benefits (expressed in quality-adjusted life years (QALYs)) between the two arms of the trial over the period of the study (further details are provided below).

METHODS AND ANALYSIS

Overall study design

The *Sleeping Sound* ASD study is an RCT where participants are allocated either to the sleep intervention group or a TAU group. The project runs from July 2016 to the end of 2019 where all stages of the project from recruitment to follow up data collection at 6-months post-randomization are executed. Participant recruitment concluded in March 2019, the collection

For peer review only

of 3- and 6- month data is ongoing. This study protocol conforms with the SPIRIT reporting requirements,[41] and a SPIRIT timeline has been provided (Figure 1).

(Insert Figure 1)

Participants

Participants include Victorian families of children with ASD aged 5 to 13 years and attending primary school at the time of recruitment. All children have a parent-reported moderate to severe sleep problem of chronic insomnia and/or delayed sleep-wake phase as defined by the International Classification for Sleep Disorders – Third Edition.[42] The intervention is designed to be feasibly delivered within the context of the Australian healthcare system, therefore, eligibility for an ASD diagnosis is based on the nationally agreed DSM-IV (Autistic Disorder, Asperger's Disorder),[43] or DSM-5 (ASD) assessment criteria.[1] All children have a diagnosis of ASD which has been confirmed by consensus of diagnosis by a multidisciplinary team specialised in ASD assessment (psychologist, paediatrician/psychiatrist, speech pathology and/or occupational therapist). Written evidence of a conclusive multidisciplinary diagnosis of ASD is sighted, or confirmed by the treating paediatrician for all children enrolled in the study. Children also met clinical ASD symptomology based on a clinical cut off score of ≥ 11 on the Social Communication Questionnaire Lifetime form.[44]

Patient involvement

The intervention used in this study is based on the ADHD *Sleeping Sound* trial during which participant feedback on the acceptability and feasibility of the study was collected of which there were no reported concerns from participants.[38] In this trial we will collecting feedback data about the program from parents.

For peer review only

Recruitment: stage 1

Recruitment through Victorian paediatric clinics. Victorian paediatricians identify potential participants meeting inclusion criteria whom they have seen in the last 12 months and send a letter inviting them to participate in the study. Study advertisements are also displayed in the waiting areas of clinical services, and disseminated widely through research, clinical and community-based networks available to investigators.

Recruitment: stage 2

The research team telephone all primary parents who register interest in the study to assess inclusion/exclusion criteria (approximately 25-minute phone call). Eligible families are provided with an information sheet, consent form (see Appendix) and baseline survey by email or post. The child's primary parent is asked to complete the survey and consent form. Online surveys are completed via a secure research database REDCap.[45] Once parents provide written consent, they are asked to provide diagnostic and cognitive assessment reports where available. If optional consent is provided to contact the child's school teacher, a link to an online survey is sent to the child's nominated teacher. Families are sent a separate hardcopy consent form for researchers to access the child's Medicare Benefits Scheme (MBS) and Pharmaceutical Benefits Scheme (PBS) data for cost-effectiveness analysis. Medicare collects information on the child's doctors' and allied health professionals' visits and the associated costs (including out-of-pocket costs). PBS collects information on prescription medications which have been recorded at pharmacies.

Participants are required to meet all inclusion criteria (see Table 1). Children currently prescribed melatonin as well as any other medications (e.g. SSRIs, etc.) are included in the trial if they continue to meet eligibility criteria. Information regarding medication is collected as part of the parent survey at time of enrolment and at 3- and 6- months follow up. We have

For peer review only

included a table documenting important changes to our inclusion/exclusion criteria after trial commencement. (See Table 2).

(Insert Table 1 and Table 2 here)

Randomization

The flow of participants from recruitment to follow-up assessments is displayed in Figure 2. Participants are randomized to either the intervention or TAU groups by an independent research assistant once consent forms and baseline surveys have been completed. Treatment allocation is determined sequentially, according to a computer-generated block randomization sequence with 1:1 ratio between groups, with blocks of randomly varying size (4, 6 and 8) developed by an independent statistician who is not involved in the project. The independent statistician will then upload this randomization list into REDCap,[45] a secure research database, to ensure allocation concealment. Given the relatively fewer diagnoses of ASD among girls,[46] randomization is stratified by gender to ensure equal representation of girls, boys and other genders (i.e. transgender, intersex) in both study arms. Siblings are eligible to participate in the study provided they each meet all inclusion criteria. Families with siblings enrolled in the study are randomized in sequence based on the return of the primary parent baseline surveys, with all subsequent siblings allocated to the same treatment group. All families are sent a letter informing them of their group allocation and are not able to enrol further siblings once notified of group allocation. A research assistant then telephones intervention families to book the intervention sessions.

The TAU group will receive standard services and support currently available in the community and complete the same study assessment follow up. If the parent consented to teacher participation, researchers approach schools for consent to contact the child's teacher and then mail the teacher a baseline survey to complete.

(Insert Figure 2 here)

For peer review only

Intervention group

Delivery of the sleep intervention. The behavioural sleep intervention is delivered by study-employed clinicians (paediatrician or psychologist experienced in working with children with ASD). Clinicians were trained over two x 3-hour interactive sessions (by HH and ES), have a detailed program manual and meet fortnightly to ensure fidelity to the program. The intervention consists of two 50-minute face-to-face consultations and one follow up phone call at two-week intervals. The content of the original *Sleeping Sound* ADHD intervention,[38] has been adapted to children with ASD to include specific visual aids and social stories to assist in the delivery of the sleep intervention for this population.[47] Intervention sessions are held in rooms at one of our study sites (Deakin University or The Royal Children's Hospital), or the treating paediatrician's consulting rooms, based on family preference.

The behavioural sleep interventions are individually tailored for the child and the family. The strategies that suit the family situation and learning style of the child with ASD are given emphasis. Delayed sleep phase, early morning waking and prolonged night waking (e.g. waking overnight where the child is unable to fall back to sleep for an hour or more) are treated with bedtime fading (i.e., the child's bedtime is temporarily set later and gradually brought forward), as well as early morning light exposure. Need for parental presence at sleep time is managed for sleep onset association using a 'camping out' method. Camping out involves parents gradually withdrawing themselves from the child's bedroom over a period of time. Parent management strategies are used for bedtime resistance and can include use of a 'bedtime pass', whereby the child can only leave the bedroom once before sleep, and may be coupled with a reward system. Primary insomnia (insomnia with no other established cause) is managed using relaxation training and restricting time in bed (i.e. bedtime fading or getting out of bed and doing a relaxing activity if the child cannot fall asleep). In addition to

For peer review only

relaxation training, examples of some strategies used to manage anxiety-related insomnia include discussing fears during the day rather than just before bedtime, encouraging the child to stay in bed and rewarding brave behaviour. Visual strategies, such as social scripts and visual timetables are used to reinforce learning.

Session One focuses on an assessment of the child's sleep problems, psychoeducation about normal sleep and sleep cycles, good sleep habits, and a tailored sleep management plan specific to the sleep diagnosis of the child. As the Sleeping Sound intervention is individually tailored to children and families, participants are encouraged to provide input in relation to goal setting and the interventions they would like to try to assist in managing the sleep problems. Families receive written handouts, summarising session content and complete a written management plan with the clinician. A standardised consultation record is kept for all children including the presenting sleep problem(s), possible contributors to the problem (e.g., TV in bedroom), medication use, comorbidities and bedtime routines. Clinicians record the duration of each consultation, sleep problem diagnoses, family sleep management goals, handouts given to parents, and management options selected. Sleep problem diagnoses made may include one or more difficulties getting to sleep (sleep anxiety, bedtime resistance, sleep association onset disorder, delayed sleep phase or insomnia), and/or night waking problems (prolonged night waking or early morning waking). Parasomnias (nightmares, night terrors, sleep walking) are recorded and psycho-education and general management advice about parasomnias is provided although these are not a focus of behavioural intervention.

Session Two is held two weeks later to review the sleep management plan (which includes a sleep diary), reinforce strategies, trouble-shoot and monitor progress.

A *follow up phone call* is conducted two weeks after the second visit to answer any further parent questions, reinforce strategies, trouble shoot and monitor progress.

For peer review only

Participants are able to withdraw from the intervention at any time. Participants are also asked for feedback of the intervention at all intervention sessions (including follow-up) and this is documented by clinicians in order to manage any unintended adverse effects of the trial.

Measures

Study measures are summarised in Table 3 below. Additional information collected at baseline includes family and child demographics such as socio-economic status (SES) and medical data (i.e., diagnosed comorbidities etc.). Medication information is captured at baseline and 3- and 6- months follow-up. Based on prior work with children with ADHD and ASD we do not anticipate any risks, side-effects or discomfort to participants in completing our measures. In the event that a parent becomes distressed when completing questionnaires related to stress or mental health we have included information to link participants to a psychologist on our research team as well as directing parents to contact their General Practitioner.

We have chosen the CSHQ as our primary outcome measure as this is a widely used and validated measure of behavioural sleep problems in children,[40] and was used in our pilot *Sleeping Sound* with ASD study.[39] It has been used to examine sleep behaviour in children with ASD and correlates highly with the objective measure of actigraphy.[48] It contains 33 items and includes eight subscales: Bedtime Resistance (e.g. *Child falls asleep in own bed*), Sleep Onset Delay (e.g. *Child falls asleep in 20 minutes after going to bed*), Sleep Duration (e.g. *Child sleeps the right amount*), Sleep Anxiety (e.g. *Child needs parent in the room to fall asleep*), Night Waking (e.g. *Child moves to someone else's bed during the night*), Parasomnias (e.g. *Child talks during sleep*), Sleep Disordered Breathing (e.g. *Child snores loudly*), and Daytime Sleepiness (e.g. *Child has difficulty getting out of bed in the morning*). A 3-point rating scale from 1 = *rarely* to *3* = *usually* is applied to each item, indicating the

For peer review only

sleep problem frequency during the past week. Some items are reverse coded and higher scores are indicative of more sleep problems. The measure has good internal consistency (α = .78) and each subscale also has adequate internal consistency (α = .93 - .56).[40]

Knowledge of participant group status will be limited to the project coordinator and study clinicians. If a family spontaneously reveals their intervention status to another team member this is noted on the participant file. Given that it is impossible to maintain participant blinding in a trial of this nature, we have carefully chosen some outcome measures that can be assessed in a blinded fashion. For example, we will include a blinded teacher-reported measure of sleep, behavioural, social and motor functioning. We will not inform teachers of the child's group status and will ask parents not to inform teachers. We will also include blinded objective child measures of executive and school-based functioning.

(Insert Table 3 here)

Sample size

In our pilot study,[39] we found a moderate-to-large effect of the intervention on sleep for total CSHQ score (standardized mean difference = 0.7). However, since effect sizes from small studies may not be replicated in larger studies, we conservatively estimate a moderate intervention effect size of 0.5 (equivalent to a 7% improvement in CSHQ scores in the intervention group relative to TAU). G*Power was used to calculate the sample size needed to detect a standardized mean difference of .5.[49] The impact of attrition and clustering (by individual and paediatrician) were taken into account by anticipating 20% drop off by follow-up and calculating design effects and subsequent required sample size, respectively. The sample size calculation was performed for alpha = .05 (two-tailed), power = 80%, intra-class correlation (ICC) = .08 for the within child's paediatrician clustering (average cluster size = 4, SD = 3), and ICC = .20 for the within person clustering. Results showed that we will need to analyse results of 188 individuals (94 in each group). Subsequent

For peer review only

Monte Carlo simulation showed that a sample of 188 observations would have sufficient precision (<10% bias for model coefficients and <5% bias for standard errors) and power (80%+) to estimate pertinent parameters in a three-level mixed effects linear regression model.[50] Allowing for 20% attrition over time, we will randomize a minimum of 234 children (117 in each group) to ensure sufficient power post intervention (primary outcome timepoint 3-months post randomization).

Strategies have been put in place to encourage participant retention and the completion of the follow-ups. These include a 4-step reminder process for completing follow up assessments and a study newsletter to promote family engagement.

Data analysis

Primary analysis will be intention-to-treat (within participant data included as per initial treatment group allocation, regardless of actual intervention received) and will compare outcomes of children in the TAU and intervention groups at the 3- and 6-month post randomization time points. Analysis of the primary outcome (i.e. child sleep problems) and all secondary outcomes (child – teacher reported daytime sleepiness, sleep hygiene, emotional and behavioural problems, social-communicative symptoms, cognitive performance, academic achievement, school attendance and quality of life; parent – parenting stress, mental health, quality of life and work attendance) will be carried out using linear mixed effects regression with results presented as mean differences (and 95% CIs). For each outcome, a single mixed effects model will be fitted incorporating baseline, 3- and 6-month data using random effects to allow for the repeated measures within an individual, and clustering of individuals within the child's paediatrician. Differences in the outcomes of intervention and TAU groups will be assessed with linear mixed models, with individuals clustered within the child's paediatrician and assessment times clustered within individuals. We will run an unadjusted model and compare this to an adjusted model. The adjusted model

For peer review only

will include the following as covariates: child gender, age, ASD symptom severity (measured using the Social Communications Questionnaire – current form,[44] medication use (obtained from the Resource Used Questionnaire) and socioeconomic status (measured according to the families residential postal code and using the index of relative socioeconomic disadvantage.[38, 51] The child's paediatrician and individual participant will be modelled as random effects and the remaining variables will be handled as fixed effects. To account for missing data, the mixed models will be fitted using conditional maximum likelihood. As this approach makes an untestable assumption that data are missing at random, i.e., missingness cannot be predicted from the variables included in the model, we will perform sensitivity analyses in the form of pattern mixture models to explore the effect of departure from this assumption. A per-protocol analysis will also be undertaken as a supplementary analysis.

The number of siblings in the trial is expected to be low based on our prior work. Even if the proposed mixed effects model were able to run with family as a clustering factor despite low number of sibling pairs, there is still some concern about obtained parameter estimates.[52] As such, alternative modelling approaches which can handle sparse data (such as generalized estimating equations) will be used as a sensitivity analysis to evaluate impact of clustering due to presence of siblings on results obtained from our model without this correction.[52]

Economic evaluation

An important secondary aim of the project will be to determine the cost-effectiveness of this intervention compared to TAU. The inclusion of the CHU9D at baseline, 3-month and 6-month follow-up will allow calculation of quality-adjusted life years (QALYs) as part of a cost-utility analysis, applying the area-under-the-curve approach.[53] A supplementary cost-effectiveness analysis will also be undertaken using the total CSHQ score as an outcome measure. The economic analysis will be conducted from a societal perspective, since

For peer review only

interventions targeting children with ASD are likely to have benefits and costs beyond the narrow health sector (e.g., ASD intervention services and productivity impacts for parents). A secondary analysis from a healthcare perspective will also be undertaken. The evaluation will first measure and value the use of healthcare resources over the period of the study between the two arms of the trial and then compare any additional costs to the additional outcomes achieved. Costs of the intervention will be measured using the financial records of the study team. Other costs incurred by children and their families will be measured using a resource use questionnaire. Components of the resource use questionnaire comprise the child's use of healthcare services, including: (i) inpatient services and emergency services, (ii) health professional visits, (iii) medication, and (iv) other resources used. The questionnaire also collects data on out-of-pocket and travel costs, as well as a child's school attendance and parent's productivity. Participants will also be asked for their permission to access their MBS and PBS data for a time period of 3 months prior to baseline up until the last follow up assessment. These data are not subject to recall bias and provide complete resource use costs (and out of pocket costs) for all Medicare and PBS reimbursed healthcare services. Non-Medicare and non-PBS funded services (e.g., state-funded ASD services) will be captured in the resource use questionnaire. Generalized linear models will be used to assess mean difference in costs and outcomes between the two arms, adjusted for any differences between groups at baseline. An incremental cost-effectiveness ratio (ICER) and an incremental cost-utility ratio (ICUR) will be calculated as the difference in average cost between the groups, divided by the difference in average CSHQ and QALYs respectively. Non-parametric bootstrapping will be undertaken from the observed cost/QALY pairs (1,000 simulated replications) to determine CIs and presented in a cost-effectiveness plane along with a cost-effectiveness acceptability curve. Sensitivity analyses will be carried out to explore the robustness of the results. While MBS and PBS data will be used for the base case

For peer review only

analysis, a sensitivity analysis will be performed using self-report data derived from the resource use questionnaire for those health services captured by MBS and PBS.

ETHICS AND DISSEMINATION

Behavioural sleep problems are a common burden associated with ASD. The proposed RCT of the *Sleeping Sound* intervention in ASD children with sleep problems will result in a significant advance in research on the treatment of sleep problems. The study is well-justified with reference to the demonstrated success of this tailored sleep intervention in ADHD,[38] our strong pilot data,[39] and the potential advantages of the intervention in alleviating social, emotional and academic problems connected to the sleep disorder in the children themselves and their parents. If the intervention is found to be effective in this sample of children who are of borderline/average intellectual ability we will subsequently be able to explore whether the intervention is successful for children with ASD who also have an intellectual disability. Treatment recommendations for treating sleep problems in children with ASD have been released.[54] The Sleep Committee of the Autism Treatment Network recommend the use of behavioural intervention as a first line therapy.[55] The Autism UK NICE clinical guidelines (#170) for sleep management recommend development of individualised behavioural intervention which includes specialist monitoring, and use of a sleep plan as a central concept of sleep management.[56] To date, the treatment of sleep disturbance in children with ASD is based on expert opinion and consensus practise, informed by limited evidence. Results from this study will inform evidence based clinical practice and guide appropriate selection and tailoring of behavioural strategies to address specific sleep problems. We aim to publish our findings in peer-reviewed journals and present our research at international conferences and community organisations. The study has been granted ethical approval by the Royal Children's Hospital (#36154), Deakin University (#2017-130), the Victorian Department of Education and Early Childhood Development (#2016_003134) and the Catholic Education

For peer review only

Office (Melbourne #0501) Human Research Ethics Committees. Important protocol changes are reported to each ethics committee via ethics amendments.

DATA MANAGEMENT

All participant data will be confidential and hardcopy data will be securely stored in locked offices for which only research team members have access to the keys. All online participant data are collected and stored in REDCap,[45] which is a secure database stored on the Deakin University network, accessible only to the research team via individual usernames and passwords. All chief investigators will have access to the final dataset. Information will be kept for seven years after the youngest participant's 18th birthday or 15 years after the completion of the study, whichever date is later. After this time the data will be destroyed.

Data from all hardcopy surveys will be entered into REDCap by a member of the research team.[45] To ensure quality data a process of data checking and cleaning will be undertaken by the research team. We will keep a register of data issues to ensure problems with data are addressed in a timely manner and conduct regular audits of data management processes.

For peer review only

Table 1
Study Inclusion and Exclusion Criteria

Inclusion criteria	
	Clinically confirmed diagnosis of DSM-IV Autistic Disorder,[43] Asperger's Disorder or DSM-5 ASD.[1] Diagnosis has been confirmed by consensus of a multidisciplinary team, as indicated by paediatrician or a confirmed multidisciplinary diagnosis as cited in a clinical report.
	Aged 5-12 years, or 13 years and attending primary school at the time of recruitment.
	Child meets clinical ASD symptomology based on a clinical cut off score of ≥ 11 on the Social Communication Questionnaire Lifetime form (40 item questionnaire measuring ASD symptoms).[44]
	Child's sleep problem(s) are moderate or severe by primary parent report and have been a problem for them over the past 4 weeks.[57]
	Meet diagnostic criteria for at least one of the following sleep problems as defined by the International Classification of Sleep disorders diagnostic criteria:[42] chronic insomnia (including problems initiating and maintaining sleep, early morning waking, sleep-onset association, bed-time resistance, and anxiety-related insomnia), or delayed sleep-wake phase.
Exclusion criteria	
	Diagnosed with an intellectual disability by parent report.
	Have any comorbid medical (e.g. nocturnal seizures, blindness) conditions that disturb regular sleep patterns, or genetic (e.g., Fragile X disorder) conditions associated with intellectual impairment based on parent report.
	Have suspected obstructive sleep apnoea (OSA). OSA is assessed using 3 OSA items from the CSHQ.[40] This scale can help identify children who may suffer from OSA. If a parent endorses items indicative of OSA, the child is contacted by the study paediatrician for further assessment. If OSA is suspected, the child is excluded from the research study and referred to appropriate clinical services. After approximately six months the family is followed up to see whether they are eligible to participate in the study (i.e. if OSA symptoms have resolved and they have ongoing behaviour sleep problems).
	Parents with insufficient English language proficiency to provide informed consent, complete study materials and/or participate in the intervention treatment program.

For peer review only

Table 2
Amendments to study protocol

'Opt out' study invitation amended to 'opt in' invitation	An 'opt out' approach was initially used to recruit through paediatricians. Parents were sent a letter from the paediatrician advising them that the research team would call them to explain the study, confirm eligibility, and invite them into the study. Parents were asked to contact the study team or their paediatrician if they *did not* wish to learn more about the study or be contacted by the research team. The paediatrician then provided the research team with the contact details of families if they did not opt out within the two-week period specified in the letter. This recruitment method was amended to an 'opt in' study invitation letter from paediatricians.
Age range amended	Age range extended to include 13-year olds who attend primary school at the time of recruitment
Social Communication Questionnaire Lifetime form cut off adjusted	Clinical cut off score lowered from ≥15 to ≥11. This measure is used to assess ASD symptomology. There is evidence in the literature to support a lower cut off score in order to capture children with higher-functioning ASD and Asperger's Disorder who may be missed by the use of a more stringent cut off score.[58]
Vineland inclusion criteria removed	The Vineland Adaptive Behaviour Scales 3rd Edition (Domain Interview Survey) was originally conducted with families over the phone as a screening tool for intellectual impairment. This was proving to be too cumbersome for families and a barrier to enrolment. It was therefore removed as a requirement for eligibility.
Comorbid medical conditions exclusion criteria amended	Exclusion criteria for comorbid conditions refined to medical conditions affecting sleep or associated with intellectual disability. Original criteria comorbid medical (e.g. epilepsy), neuropsychiatric (e.g. Tourette's) or syndromic genetic (e.g. Fragile X) conditions

For peer review only

Table 3

Summary of measures included in the study

Outcomes	Measure	Source	B	3	6
**Child outcomes**					
**Sleep problems**	**Children's Sleep Habits Questionnaire (CSHQ).** 33-item validated measure of sleep that can distinguish clinical from community samples.[40] Provides a measure of total sleep problems and eight subscale scores reflecting major behavioural sleep disorders (bedtime resistance, sleep onset delay, sleep duration, sleep anxiety, night waking, parasomnias, sleep disordered breathing, daytime sleepiness).	Parent	●	●	●
**Sleep hygiene**	**Sleep Hygiene Scale.** 7-item study developed measure adapted from the Bedroom Routines Scale.	Parent	●	●	●
**Daytime Sleepiness**	**Teacher Daytime Sleepiness Questionnaire.** 10 item validated scale of daytime sleepiness at school.[59]	Teacher	●	●	●
**Social-Communication functioning**	**Social Communication Questionnaire (SCQ) Current** form. 40-item measure of ASD symptoms in past 3 months to measure change in ASD social-communication symptoms over time.[44]	Parent	●	●	●
**Emotional and behavioural disturbance**	**Developmental Behavioural Checklist (DBC).** 96-item measure of emotional and behavioural disturbance in children.[60] Provides a rating of overall behavioural disturbance and five subscales: disruptive/antisocial behaviour, self-absorbed, communication disturbance, anxiety and ASD social relating.	Parent	●		●
**Behavioural and social functioning**	**Strengths and Difficulties Questionnaire (SDQ).** 25 items assessing the following subscales: hyperactivity/inattention, conduct problems, emotional symptoms, peer relationship problems, and prosocial behaviour.[61]	Parent & Teacher	●	●	●
**Quality of life**	**Child Health Utility 9D (CHU9D).** 9 item measure of child quality of life.[53]	Parent	●	●	●
**School attendance**	**School attendance** over the preceding 3 months.	Parent	●	●	●
**Academic functioning**	**Wide Range Achievement Test 4 (WRAT).** Spelling and math computation subtests to assess academic functioning.[62]	Child			●
**Academic functioning**	Permission to access **NAPLAN** results (Years 3, 5, 7). NAPLAN is a national assessment of literacy and numeracy Australian children complete in Grades 3, 5, 7 and 9.	Linkage			
**Cognitive functioning**	**NIH Toolbox** – Cognitive Domain Tasks to assess cognitive functioning.[63] iPad administered assessment of executive function, attention, episodic/working memory, processing speed, language abilities, new learning and reading.	Child			●
**Parent outcomes**					
**Mental health**	**Kessler 10 (K10).** A 10-item validated measure of adult psychological distress.[64]	Parent	●	●	●
**Parenting stress**	**Parenting Stress Index 4SF (PSI-4SF).** 36 item measure of parenting stress.[65] Provides a measure of total parenting stress and three subscales reflecting the major sources of parenting stress (parental distress, difficult child, parent-child dysfunctional interaction).	Parent	●	●	●
**Quality of life**	**Assessment of Quality of Life (AQoL4D).** 12 item measure of parent quality of life.[66]	Parent	●	●	●

**Economic outcomes**					
**Resource use**	Family reported **service use** over preceding 3 months	Parent	●	●	●
**Health service utilisation**	Permission to access both **Medicare and Pharmaceutical Benefits Scheme (PBS)** data for 3 months prior to baseline up until the last follow-up assessment.	Linkage	●	●	●

● measure utilised at indicated timepoint.
B, baseline; 3, 3 months; 6, 6 months

For peer review only

Author's contributions

Nicole Rinehart, Nicole Papadopoulos, Emma Sciberras, Harriet Hiscock, Katrina Williams, Jane McGillivray, Pat Howlin and Cathrine Mihalopoulos conceived the study. Susannah Bellows, Deborah Marks and Lidia Engel contributed to the writing of the methods and intervention for the study. Nicole Papadopoulos and Susannah Bellows drafted the manuscript. Matthew Fuller-Tyszkiewicz contributed to the design and analytic components of the study. All authors contributed, read and approved the final manuscript.

Funding

This work was supported by the Australian National Health and Medical Research Council (NHMRC; project grant number APP1101989). Associate Professor Sciberras is funded by an NHMRC Career Development Fellowship 1110688 (2016-21) and a veski Inspiring Women's Fellowship. Harriet Hiscock is supported by an NHMRC Practitioner fellowship 1136222 (2018-2022). The MCRI is supported by the Victorian Government's Operational Infrastructure Support Program.

Competing interests

Professor Rinehart and Professor McGillivray receive funding from the Moose Foundation and the Australian Football League to conduct research in the field of neurodevelopmental disorders and inclusion. Professor Rinehart, Professor McGillivray and Dr Papadopoulos also received funding from the Ferrero Group Australia as part of its Kinder + Sport pillar of Corporate Social Responsibility initiatives to promote active lifestyles among young people. Professor Rinehart has previously received speaker honorarium from Novartis (2002), Pfzier (2006) and Nutricia (2007) and is a Director of the Amaze Board (Autism Victoria). None of the companies or organisational bodies listed above had a role in this research including the

For peer review only

collection, analysis, and interpretation of data; in writing of the manuscript; and /or in the decision to submit the article for publication.

Exclusive license

I, the Submitting Author have the right to grant and does grant on behalf of all authors of the Work (as defined in the below author license), an exclusive license and/or a non-exclusive license for contributions from authors who are: i) UK Crown employees; ii) where BMJ has agreed a CC-BY license shall apply, and/or iii) in accordance with the terms applicable for US Federal Government officers or employees acting as part of their official duties; on a worldwide, perpetual, irrevocable, royalty-free basis to BMJ Publishing Group Ltd ("BMJ") its licensees and where the relevant Journal is co-owned by BMJ to the co-owners of the Journal, to publish the Work in BMJ Open and any other BMJ products and to exploit all rights, as set out in our license.

The Submitting Author accepts and understands that any supply made under these terms is made by BMJ to the Submitting Author unless you are acting as an employee on behalf of your employer or a postgraduate student of an affiliated institution which is paying any applicable article publishing charge ("APC") for Open Access articles. Where the Submitting Author wishes to make the Work available on an Open Access basis (and intends to pay the relevant APC), the terms of reuse of such Open Access shall be governed by a Creative Commons license – details of these licenses and which Creative Commons license will apply to this Work are set out in our license referred to above.

Figure legend

Figure 1. Study timeline, figure format obtained from the SPIRIT 2013 statement.

Figure 2. Flow of participants, figure format obtained from the CONSORT 2010 statement.

For peer review only

References

1. American Psychiatric Association. Diagnostic and Statistical Manual of Mental Disorders. 5th ed. Arlington, VA: American Psychiatric Publishing 2013.

2. Australian Institute of Health and Welfare. Autism in Australia 2017 [cited 2018 August]. Available from: https://www.aihw.gov.au/reports/disability/autism-in-australia/contents/autism.

3. Synergies Economic Consulting. Economic costs of autism spectrum disorder in Australia: updated study. 2011.

4. Muris P, Steerneman P, Merckelbach H, Holdrinet I, Meesters C. Comorbid anxiety symptoms in children with pervasive developmental disorders. *J Anxiety Disord* 1998;12(4):387-93.

5. White SW, Oswald D, Ollendick T, Scahill L. Anxiety in children and adolescents with autism spectrum disorders. *Clin Psychol Rev* 2009;29(3):216-29.

6. Gadow KD, Devincent CJ, Pomeroy J, Azizian A. Comparison of DSM-IV symptoms in elementary school-age children with PDD versus clinic and community samples. *Autism* 2005;9(4):392-415.

7. Simonoff E, Pickles A, Charman T, Chandler S, Loucas T, Baird G. Psychiatric disorders in children with autism spectrum disorders: prevalence, comorbidity, and associated factors in a population-derived sample. *Journal of the American Academy of Child & Adolescent Psychiatry* 2008;47(8):921-29.

8. Beresford B, McDaid C, Parker A, et al. Pharmacological and non-pharmacological interventions for non-respiratory sleep disturbance in children with neurodisabilities: a systematic review. *Health technology assessment* 2018;22(60):1-295.

For peer review only

9. Cohen S, Conduit R, Lockley SW, Rajaratnam SMW, Cornish KM. The relationship between sleep and behavior in autism spectrum disorder (ASD): a review. *J Neurodev Disord* 2014;6(44):1-10.

10. Richdale AL, Prior MR. The sleep-wake rhythm in children with autism. *European Journal of Adolescent Psychiatry* 1995;4:175-86.

11. Krakowiak P, Goodlin-Jones B, Hertz-Picciotto I, Croen LA, Hansen RL. Sleep problems in children with autism spectrum disorders, developmental delays, and typical development: a population-based study. *J Sleep Res* 2008;17(2):197-206.

12. Liu X, Hubbard JA, Fabes RA, Adam JB. Sleep disturbances and correlates of children with autism spectrum disorders. *Child Psychiatry and Human Development* 2006;37:179-91.

13. Sivertsen B, Posserud M-B, Gillberg C, Lundervold AJ, Hysing M. Sleep problems in children with autism spectrum problems: a longitudinal population-based study. *Autism* 2012;16(2):139-50.

14. Richdale AL. Sleep disorders in autism and Asperger's syndrome. In: Stores G, Wiggs L, editors. Sleep Disturbances in Disorders of Development: Its Significance and Management. London: MacKeith Press 2001. p. 181–91.

15. Richdale AL, Schreck KA. Sleep problems in autism spectrum disorders: prevalence, nature, & possible biopsychosocial aetiologies. *Sleep Med Rev* 2009;13(6):403-11.

16. Leyfer OT, Folstein SE, Bacalman S, et al. Comorbid psychiatric disorders in children with autism: interview development and rates of disorders. *Journal of Autism and Developmental Disorders* 2006;36(7):849-61.

17. Goldman SE, McGrew S, Johnson KP, Richdale AL, Clemons T, Malow BA. Sleep is associated with problem behaviors in children and adolescents with autism spectrum disorders. *Research in Autism Spectrum Disorders* 2011;5(3):1223-29.

For peer review only

18. Fadini CC, Lamônica DA, Fett-Conte AC, et al. Influence of sleep disorders on the behavior of individuals with autism spectrum disorder. *Frontiers in Human Neuroscience* 2015;9:347.

19. Tudor ME, Hoffman CD, Sweeney DP. Children with autism: sleep problems and symptom severity. *Focus on Autism and Other Developmental Disabilities* 2012;27(4):254-62.

20. Malow BA, Marzec MS, McGrew SG, Wang L, Henderson LM, Stone WL. Characterizing sleep in children with autism spectrum disorders: a multidimensional approach. *SLEEP* 2006;29(12):1563-71.

21. Richdale AL, Baglin CL. Self-report and caregiver-report of sleep and psychopathology in children with high-functioning autism spectrum disorder: a pilot study. *Dev Neurorehabil* 2015;18(4):272-79.

22. Delahaye J, Kovacs E, Sikora D, et al. The relationship between health-related quality of life and sleep problems in children with autism spectrum disorders. *Research in Autism Spectrum Disorders* 2014;8(3):292-303.

23. Taylor MA, Schreck KA, Mulick JA. Sleep disruption as a correlate to cognitive and adaptive behavior problems in autism spectrum disorders. *Res Dev Disabil* 2012;33(5):1408-17.

24. Doo S, Wing YK. Sleep problems of children with pervasive developmental disorders: correlation with parental stress. *Developmental Medicine and Child Neurology* 2006;48(8):650-55.

25. Johnson CR, Smith T, DeMand A, et al. Exploring sleep quality of young children with autism spectrum disorder and disruptive behaviors. *Sleep Med* 2018;44:61-66.

For peer review only

26. Honomichl RD, Goodlin-Jones BL, Burnham M, Gaylor E, Anders TF. Sleep patterns of children with pervasive developmental disorders. *Journal of Autism and Developmental Disorders* 2002;32(6):553-61.

27. Hodge D, Hoffman CD, Sweeney DP, Riggs ML. Relationship between children's sleep and mental health in mothers of children with and without autism. *Journal of Autism and Developmental Disorders* 2013;43(4):956-63.

28. Hoffman CD, Sweeney DP, Lopez-Wagner MC, Hodge D, Nam CY, Botts BH. Children with autism: sleep problems and mothers' stress. *Focus on Autism and Other Developmental Disabilities* 2008;23(3):155-65.

29. Tilford JM, Payakachat N, Kuhlthau KA, et al. Treatment for sleep problems in children with autism and caregiver spillover effects. *Journal of Autism and Developmental Disorders* 2015;45(11):3613-23.

30. Gringras P, Gamble C, Jones AP, et al. Melatonin for sleep problems in children with neurodevelopmental disorders: randomised double masked placebo controlled trial. *BMJ* 2012;345:e6664.

31. Vriend JL, Corkum PV, Moon EC, Smith IM. Behavioral interventions for sleep problems in children with autism spectrum disorders: current findings and future directions. *J Pediatr Psychol* 2011;36(9):1017-29.

32. Rigney G, Ali NS, Corkum P, et al. A systematic review to explore the feasibility of a behavioural sleep intervention for insomnia in children with neurodevelopmental disorders: a transdiagnostic approach. *Sleep Med Rev* 2018;41:244-54.

33. Adkins KW, Molloy C, Weiss SK, et al. Effects of a standardized pamphlet on insomnia in children with autism spectrum disorders. *Pediatrics* 2012;130(Supplement 2):S139-S44.

For peer review only

34. Johnson CR, Turner KS, Foldes E, Brooks MM, Kronk R, Wiggs L. Behavioral parent training to address sleep disturbances in young children with autism spectrum disorder: a pilot trial. *Sleep Med* 2013;14(10):995-1004.

35. Wiggs L, Stores G. Behavioural treatment for sleep problems in children with severe learning disabilities and challenging daytime behaviour: effect on sleep patterns of mother and child. *J Sleep Res* 1998;7(2):119-26.

36. Simonds JF, Parraga H. Prevalence of sleep disorders and sleep behaviors in children and adolescents. *Journal of the American Academy of Child & Adolescent Psychiatry* 1982;21(4):383-88.

37. Malow BA, Adkins KW, Reynolds A, et al. Parent-based sleep education for children with autism spectrum disorders. *Journal of Autism and Developmental Disorders* 2014;44(1):216-28.

38. Hiscock H, Sciberras E, Mensah F, et al. Impact of a behavioral sleep intervention on ADHD symptoms, child sleep and parent health: a randomized controlled trial. *The BMJ* 2015;350:1-14.

39. Papadopoulos N, Sciberras E, Hiscock H, Mulraney M, McGillvray J, Rinehart N. The efficacy of a brief behavioural sleep intervention in school-aged children with ADHD and comorbid autism spectrum disorder. *J Atten Disord* 2015:1-10.

40. Owens JA, Spirito A, McGuinn M. The Children's Sleep Habits Questionnaire (CSHQ): psychometric properties of a survey instrument for school-aged children. *SLEEP* 2000;23(8):1043-51.

41. Chan A-W, Tetzlaff JM, Altman DG, et al. SPIRIT 2013 statement: defining standard protocol items for clinical trials. *Ann Intern Med* 2013;158(3):200-07.

For peer review only

42. American Academy of Sleep Medicine. International Classification of Sleep Disorders (ISCD-3). 3rd ed. Darian, IL: American Academy of Sleep Medicine 2014.

43. American Psychiatric Association. Diagnostic and Statistical Manual of Mental Disorders. 4th ed. Washington, DC: Author 2000.

44. Rutter M, Bailey A, Lord C. The Social Communication Questionnaire. Los Angeles: Western Psychological Services 2003.

45. Harris PA, Taylor R, Thielke R, Payne J, Gonzalez N, Conde JG. Research electronic data capture (REDCap): a metadata-driven methodology and workflow process for providing translational research informatics support. *J Biomed Inform* 2009;42(2):377-81.

46. Kim YS, Leventhal BL, Koh Y-J, et al. Prevalence of autism spectrum disorders in a total population sample. *Am J Psychiatry* 2011;168(9):904-12.

47. Sivaratnam CSea. Brief report: assessment of the social-emotional profile in children with autism spectrum disorders using a novel comic strip task. *Journal of Autism and Developmental Disorders* 2012;42(11):2505-12.

48. Souders MC, Mason TBA, Valladares O, et al. Sleep behaviors and sleep quality in children with autism spectrum disorders. *SLEEP* 2009;32(12):1566.

49. Faul F, Erdfelder E, Lang A-G, Buchner A. G*Power 3: A flexible statistical power analysis program for the social, behavioral, and biomedical sciences. *Behav Res Methods* 2007;39(2):175-91.

50. Muthén LK, Muthén BO. How to use a Monte Carlo study to decide on sample size and determine power. *Structural equation modeling* 2002;9(4):599-620.

51. Australian Bureau of Statistics. Census of population and housing: Socio-economic indexes for areas (SEIFA), Australia, 2016 (2033.0.55.001) Canberra: Author; 2018 [cited

For peer review only

2019 February]. Available from:
https://www.abs.gov.au/ausstats/abs@.nsf/mf/2033.0.55.001.

52. Sauzet O, Peacock JL. Binomial outcomes in dataset with some clusters of size two: can the dependence of twins be accounted for? A simulation study comparing the reliability of statistical methods based on a dataset of preterm infants. *BMC Med Res Methodol* 2017;17(1):110.

53. Stevens K. Valuation of the Child Health Utility 9D Index. *PharmacoEconomics* 2012;30(8):729-47.

54. Abel E, Kim SY, Kellerman AM, Brodhead MT. Recommendations for identifying sleep problems and treatment resources for children with autism spectrum disorder. *Behavioral Analysis Practice* 2017;10:261-69.

55. Malow BA, Byars K, Johnson K, et al. A practice pathway for the identification, evaluation, and management of insomnia in children and adolescents with autism spectrum disorders. *Pediatrics* 2012;130(Supplement 2):S106-S24.

56. National Institute for Health and Care Excellence. Autism: the management and support of children and young people on the autism spectrum (Clinical guideline 170) 2013 [cited 2018 August]. Available from: http://guidance.nice.org.uk/CG170.

57. Hiscock H, Canterford L, Ukoumunne OC, Wake M. Adverse associations of sleep problems in Australian preschoolers: national population study. *Pediatrics* 2007;119(1):86-93.

58. Corsello C, Hus V, Pickles A, et al. Between a ROC and a hard place: decision making and making decisions about using the SCQ. *J Child Psychol Psychiatry* 2007;48(9):932-40.

For peer review only

59. Owens JA, Spirito A, McGuinn M, Nobile C. Sleep habits and sleep disturbance in elementary school-aged children. *Journal of Developmental and Behavioral Pediatrics* 2000;21(1):27-36.

60. Einfield SL, Tonge BJ. Manual for the Developmental Behaviour Checklist: Primary Carer Version (DBC-P) and Teacher (DBC-T) Version. Melbourne, Australia: Monash University Centre for Developmental Psychiatry and Psychology 2002.

61. Goodman R. Psychometric properties of the strengths and difficulties questionnaire. *Journal of the American Academy of Child & Adolescent Psychiatry* 2001;40:1337-45.

62. Wilkinson GS, Robertson GJ. WRAT 4: Wide Range Achievement Test; Professional Manual: Psychological Assessment Resources, Incorporated 2006.

63. Gershon RC, Cella D, Fox NA, Havlik R, J., Hendrie HC, M.V. W. Assessment of neurological and behavioural function: the NIH Toolbox. *Lancet Neurol* 2010;9(2):138-39.

64. Kessler RC, Barker PR, Colpe LJ, Epstein JF, Gfroerer JC, Hiripi E. Screening for serious mental illness in the general population. *Archives of General Psychiatry* 2003;60(2):184-9.

65. Abidin RR. Parenting Stress Index. 4 ed. Lutx, FL: PAR 2012.

66. Richardson J, Hawthorne G. The Australian Quality of Life (AQoL) instrument: psychometric properties of the descriptive system and initial validation. *Australian Studies of Health Service Administration* 1998;85:315-42.

For peer review only

	STUDY PERIOD				
	**Enrolment**		**Post-randomization**		**Close out**
**TIMEPOINT**	***Baseline***	**Randomization**	***3m***	***6m***	
**ENROLMENT:**					
Eligibility screening	X				
Informed consent	X				
Baseline parent survey[a]	X				
Randomization		X			
**INTERVENTIONS:**					
Sleeping sound intervention		●——	——●		
Treatment as usual					
**ASSESSMENTS:**					
Parent survey[a]	X		X	X	
Teacher survey[a]	X		X	X	
Child assessment[a]				X	
Medicare/PBS linkage					X
NAPLAN linkage					X

Note. [a] Refer to Table 3 for the specific measures included.
Figure 1. SPIRIT timeline

For peer review only

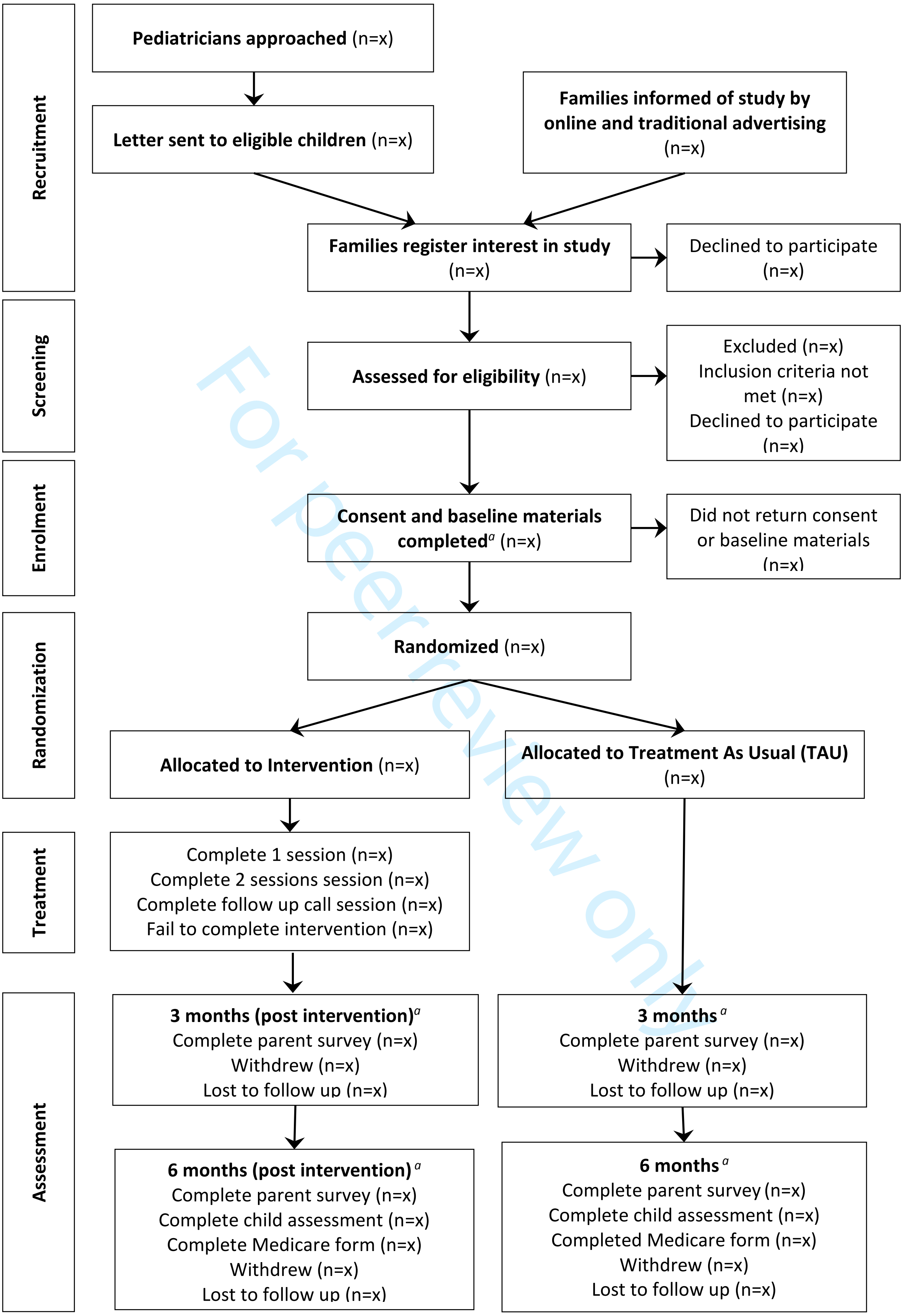

Note. [a] Complete teacher survey if applicable
Figure 2: Flow of participants

For peer review only

Appendix

Participant Information Sheet and Consent Form – Parent Version

Participant Information Sheet/Consent Form – Parent/Guardian

Interventional Study - *Parent/Guardian consenting on behalf of participant*

**HREC Project Number:**	HREC/16/RCHM/73		
**Research Project Title:**	**Sleeping Sound with Autism Spectrum Disorder (ASD):** Tailoring a behavioural sleep intervention for children with ASD: a randomized controlled trial		
**Principal Researcher:**	Professor Nicole Rinehart, School of Psychology, Deakin University		
**Version Number:**	7	**Version Date:**	17/9/2017

Thank you for taking the time to read this **Parent/Guardian Information Statement and Consent Form**. We would like to invite your child to participate in a research project that is explained below. This document is 5 pages long. Please make sure you have all the pages.

What is an Information Statement?
These pages tell you about the research project. It explains to you clearly and openly all the steps and procedures of the project. The information is to help you decide whether or not you would like your child to take part in the research. Please read this Information Statement carefully.

Before you decide if you want your child to take part or not, you can ask us any questions you have about the project. You may want to talk about the project with your family, friends or health care worker.

Important things you need to know
- It is your choice whether or not your child can take part in the research. You do not have to agree if you do not want to
- If you decide you do not want your child to take part, it will not affect the treatment and care your child gets at The Royal Children's Hospital or with your paediatrician.

If you would like your child to take part in the research project, please sign the consent form at the end of this information statement. By signing the consent form you are telling us that you:

- understand what you have read
- had a chance to ask questions and received satisfactory answers
- consent to your child taking part in the project

We will give you a copy of this information and consent form to keep.

---

1. What is the research project about?

This research project is about sleep in children with an Autism Spectrum Disorder (ASD). Up to 85% of children with ASD have sleep problems, such as difficulty falling asleep, resisting

For peer review only - http://bmjopen.bmj.com/site/about/guidelines.xhtml

going to bed and being tired in the morning. We know sleep problems in children with ASD can be related to poorer child and family functioning.

In 2013, we ran a small study that looked at whether a sleep program improved sleep, child and family wellbeing in children with ASD. We found that a 2-3 session sleep program improved these outcomes. This program has already been shown to be helpful for children with ADHD.

This year, we aim to offer this sleep program to a larger group of children with ASD who have sleep problems. To know whether this program helps, we need to compare children who receive the program with children who do not. We hope about 235 children with ASD and their families will take part in this project.

To take part in the study, children need to be aged between 5 and 12 years of age (or 13 years of age and attending primary school), have problems with sleeping and a diagnosis of ASD without an intellectual disability.

2. Who is funding this research project?

This research is fully funded by the National Health and Medical Research Council.

3. Why is my child being asked to take part?

We are asking you and your child to take part because your child has ASD and is aged between 5 and 12 years (or is 13 years and attending primary school).

4. What does my child need to do in this research project?

Start of study
We will telephone you to tell you more about the project and see if your child is eligible. This will involve asking you some questions about your child's sleep, autism symptoms, wellbeing and daily functioning. This call will be conducted in two phone calls, each of which will take between 20 minutes and 40 minutes. We will also ask to contact your child's paediatrician for confirmation of ASD diagnosis. If your child is not eligible for the study, the data you provide will only be reported in summary form (e.g. a total of 7 children were not eligible for the study as they had an intellectual disability).

If your child is eligible to take part, we will ask you to complete a survey about your child's autism and sleep, and about your child and family's wellbeing. This will take about 20 minutes to do and you can complete it online or send it back to us in the reply-paid envelope provided if you are completing it in hard copy. Once we have received your survey, we will randomly place you in one of two groups. This will be done by chance, similar to tossing a coin, so you will have an equal chance of being in either group. We will send you a letter to let you know which group you have been placed in. What you will do next will depend on which group you are allocated to.

- **Usual Care Group**

If you are in the Usual Care Group, we will contact you again in 3, 6- and 12-months' time and ask you to complete surveys about your child's autism and sleep, and child and family wellbeing. Each survey will take about 20 minutes to fill out.

For peer review only

- **Sleep Program Group**

If you are allocated to the Sleep Program Group, we will offer you two 50-minute visits and a follow up phone call with a clinician (psychologist or paediatrician) who has been trained to help you manage your child's sleep problems. At the sleep visits, the clinician will assess your child's sleep and provide you with some sleep strategies. These visits are free. These visits are done privately with the clinician, yourself and your child at Deakin University (Burwood or Geelong), the Royal Children's Hospital/MCRI (Parkville), or your paediatrician's office – there will be no other families at the visit. We will then send you surveys to complete in 3, 6- and 12-months' time. Each survey asks about your child's ASD and sleep, and child and family wellbeing and will take about 20 minutes to fill out.

6 months after start of study – participants in both groups

We will arrange a time to complete a face-to-face learning assessment with your child. It will take about 60 minutes to do. The assessment is non-invasive and most children enjoy doing it. We can visit you at your home to do this assessment or you can visit us at The Royal Children's Hospital in Melbourne, or Deakin University in Burwood or Geelong. We will provide you with a brief report from this assessment if requested.

5. Optional consent

We would like you to consider the following optional parts of this project. You may agree to none, some or all optional parts. However, it is your decision, and if you choose not to agree to any parts, it will not affect your participation in the rest of the study. Please complete the optional consent tick boxes on the consent form to record your decision for each option.

A. **Contacting your child's school teacher:** We would like to ask your child's teacher to fill out a short survey about your child's behaviour to find out if sleep affects your child at school. We will ask them to complete the survey on four occasions. We will contact the kindergarten director or school principal and gain their approval to ask your child's teacher to do this. We will not refer to ASD in the information we send to the teacher or the principal/director. Instead, we will say that this is a project about managing sleep problems in children. The information provided by the teacher in the survey will not be shared with you or your child.

B. **NAPLAN.** We would like your permission to get your child's NAPLAN results. Your child's NAPLAN results will be de-identified and stored in a password-secured database, only accessible by the research team. Please indicate on the consent form if you consent to us getting your child's NAPLAN results.

C. **Assessment records.** We would like to access the results of any assessments related to ASD which your child may have done in the past, including autism assessments, IQ, adaptive functioning and language assessments. We will use this information to help us understand whether the intervention is useful for children with a range of abilities. If you do not have copies of these reports yourself, with your permission we will request them from health professionals or hospitals your child has visited.

D. **Re-contact for future research:** We would like to offer you the chance to hear about new research projects that are related to this project. By ticking this consent you are only consenting to hear about new projects only. You can decide at the time whether or not you want your child to take part in any future project.

E. **Medicare and Pharmaceutical Benefits Scheme (PBS):** We would like to access your child's complete Medicare and PBS data. This will help us to see whether families who get the sleep program access fewer health services and medicines than those who

For peer review only

do not get the program. Medicare collects information on your child's doctors' visits and the associated costs, while the PBS collects information on the prescription medications you have filled at pharmacies for your child. We will ask you to fill out this additional consent form at the time of your child's 6 month follow up, which will allow us to access this data as outlined on the back of the additional consent form we will give you at that time. This consent form will then be sent securely to the Department of Human Services who holds this information confidentially.

6. Can my child withdraw from the project?

If you give your consent and change your mind, your child can withdraw from the project. If you decide to withdraw, please notify the research team. You do not need to tell us the reason why you or your child want to stop being in the project. If your child leaves the project we will use any information already collected unless you tell us not to do so.

7. What are the possible benefits for my child and other people in the future?

If you are in the **Sleep Program Group**, the clinician will provide strategies and information to help you with your child's sleep problem. If your child has other difficulties other than sleep problems (e.g. anxiety, ADHD symptoms), these will not be directly addressed by the Sleeping Sound intervention – the study clinician will encourage you to discuss these problems with your treating paediatrician. We hope that your child will have fewer sleep problems and this may help his/her behaviour as well as your child and family's wellbeing.

If you are in the **Usual Care Group**, you and your child will not receive any direct benefit.

We are doing this project to see if this sleep program works or not. This means that we can't guarantee that your child and family will benefit, even if you are in the **Sleep Program Group.**

Whether you receive the sleep program or not, your participation will help us learn whether the sleep program is helpful for families of children with ASD. If it is helpful, then the project will be the first to provide information which may help future children with sleep problems and ASD.

8. What are the possible risks, side-effects, discomforts and/or inconveniences?

We do not anticipate risks, side-effects or discomforts for you or your child. In our previous *Sleeping Sound with ADHD* project, parents did not report any side-effects or discomforts in their feedback surveys.

9. What will be done to make sure my child's information is confidential?

In this study we will collect and use personal and health information about your child for research purposes. Any information we collect that can identify your child will be treated as confidential. It will be used only in this project, unless otherwise specified. We can disclose the information only with your permission, except as required by law. All information will be stored securely at Deakin University. Information will be kept electronically on a secure Deakin University server, in a password protected database (REDCap) or password protected computer. All hard copy consent forms, surveys and assessments will be stored securely in a locked cabinet at Deakin University.

For peer review only

The following people may access information collected as part of this research project:

- The research team involved with this project
- The Royal Children's Hospital Human Research Ethics Committee

The information will be re-identifiable. This means that we will remove your child's name and give the information a special code number. Only the research team can match your child's name to their code number, if it is necessary to do so.

Information will be kept until the youngest participant turns 25 years old. The research information may be destroyed or kept indefinitely in secure storage after this time. However, Medicare/PBS data will be kept for 15 years only from the date of collection. After this time, the consent paper copies will be shredded in a manner that makes re-identification of the data impossible, and the electronic Medicare and PBS data will be deleted.

In accordance with relevant Australian and/or Victorian privacy and other relevant laws, you have the right to access and correct the information we collect and store about your child. Please contact us if you would like to access this information.

At the end of the study, results may be presented at conferences or published in medical journals. This will be done in such a way that your child cannot be identified.

10. Will we be informed of the results when the research project is finished?

Participants in the Sleep Program group and the Usual Care group will receive a summary of the results at the end of this project, which will say whether the intervention was successful. This summary will give overall results of this project – individual families will not be identified. Participants in the Usual Care group **will not** be offered the Sleeping Sound intervention after completion of the study.

11. Who should I contact for more information?

If you would like more information about the project or if you need to speak to a member of the research team in an emergency please contact:

**Name:**	Susannah Bellows
**Contact telephone:**	9246 8937
**Email:**	sleepingsound@deakin.edu.au

If you have any concerns and/or complaints about the project, the way it is being conducted or your child's rights as a research participant, and would like to speak to someone independent of the project, please contact: Director, Research Ethics & Governance, The Royal Children's Hospital Melbourne on telephone: (03) 9345 5044.

For peer review only

CONSENT FORM

**HREC Project Number:**	HREC/16/RCHM/73
**Research Project Title:**	Sleeping Sound with Autism Spectrum Disorder (ASD): Tailoring a behavioural sleep intervention for children with ASD: a randomized controlled trial

Version Number: 7 **Version Date:** 17/09/2017

- I have read, or someone has read to me in a language that I understand, the information statement version listed above and I understand its contents.
- I believe I understand the purpose, extent and possible risks of our involvement in this project.
- I voluntarily consent for me and my child to take part in this research project.
- I have had an opportunity to ask questions and I am satisfied with the answers I have received.
- I understand that this project has been approved by The Royal Children's Hospital Melbourne Human Research Ethics Committee and will be carried out in line with the National Statement on Ethical Conduct in Human Research (2007) – including all updates.
- I understand I will receive a copy of this Information Statement and Consent Form.

OPTIONAL CONSENT

☐ I do	☐ I do not	consent for the researchers to contact my child's school teacher to complete a survey
☐ I do	☐ I do not	consent for VCAA (Victorian Curriculum and Assessment Authority) to release my child's years 3, 5, and 7 NAPLAN results to the Sleeping Sound with ASD Project.
☐ I do	☐ I do not	consent for the researchers to access copies of my child's assessments and medical records related to autism
☐ I do	☐ I do not	consent to be contacted about future research projects that are related to this project.
☐ I do	☐ I do not	consent to the storage and use of my child's information in future ethically-approved research related to this research project.

Child's Name

Parent/Guardian Name | Parent/Guardian Signature | Date

Name of Witness to Parent/Guardian's Signature | Witness Signature | Date

For peer review only

Declaration by researcher: I have explained the project to the parent/guardian who has signed above, and believe that they understand the purpose, extent and possible risks of their and their child's involvement in this project.

Research Team Member Name	Research Team Member Signature	Date

Note: All parties signing the Consent Form must date their own signature.

For peer review only

SPIRIT
STANDARD PROTOCOL ITEMS: RECOMMENDATIONS FOR INTERVENTIONAL TRIALS

SPIRIT 2013 Checklist: Recommended items to address in a clinical trial protocol and related documents*

Section/item	Item No	Description	Addressed on page number
**Administrative information**			
Title	1	Descriptive title identifying the study design, population, interventions, and, if applicable, trial acronym	______1______
Trial registration	2a	Trial identifier and registry name. If not yet registered, name of intended registry	______5______
	2b	All items from the World Health Organization Trial Registration Data Set	_____N/A_____
Protocol version	3	Date and version identifier	______5______
Funding	4	Sources and types of financial, material, and other support	_____ 2______
Roles and responsibilities	5a	Names, affiliations, and roles of protocol contributors	_____1-2______
	5b	Name and contact information for the trial sponsor	_____N/A____
	5c	Role of study sponsor and funders, if any, in study design; collection, management, analysis, and interpretation of data; writing of the report; and the decision to submit the report for publication, including whether they will have ultimate authority over any of these activities	_____N/A______
	5d	Composition, roles, and responsibilities of the coordinating centre, steering committee, endpoint adjudication committee, data management team, and other individuals or groups overseeing the trial, if applicable (see Item 21a for data monitoring committee)	_____N/A______

For peer review only - http://bmjopen.bmj.com/site/about/guidelines.xhtml

**Introduction**			
Background and rationale	6a	Description of research question and justification for undertaking the trial, including summary of relevant studies (published and unpublished) examining benefits and harms for each intervention	____6-11______
	6b	Explanation for choice of comparators	____10________
Objectives	7	Specific objectives or hypotheses	____10________
Trial design	8	Description of trial design including type of trial (eg, parallel group, crossover, factorial, single group), allocation ratio, and framework (eg, superiority, equivalence, noninferiority, exploratory)	____11-12_____
**Methods: Participants, interventions, and outcomes**			
Study setting	9	Description of study settings (eg, community clinic, academic hospital) and list of countries where data will be collected. Reference to where list of study sites can be obtained	____11-14______
Eligibility criteria	10	Inclusion and exclusion criteria for participants. If applicable, eligibility criteria for study centres and individuals who will perform the interventions (eg, surgeons, psychotherapists)	__13-14, 24-25___
Interventions	11a	Interventions for each group with sufficient detail to allow replication, including how and when they will be administered	____15-17______
	11b	Criteria for discontinuing or modifying allocated interventions for a given trial participant (eg, drug dose change in response to harms, participant request, or improving/worsening disease)	____16, 17_____
	11c	Strategies to improve adherence to intervention protocols, and any procedures for monitoring adherence (eg, drug tablet return, laboratory tests)	_____16_______
	11d	Relevant concomitant care and interventions that are permitted or prohibited during the trial	_____13_______
Outcomes	12	Primary, secondary, and other outcomes, including the specific measurement variable (eg, systolic blood pressure), analysis metric (eg, change from baseline, final value, time to event), method of aggregation (eg, median, proportion), and time point for each outcome. Explanation of the clinical relevance of chosen efficacy and harm outcomes is strongly recommended	_____17, 26-27__
Participant timeline	13	Time schedule of enrolment, interventions (including any run-ins and washouts), assessments, and visits for participants. A schematic diagram is highly recommended (see Figure)	_____12, 14____

For peer review only - http://bmjopen.bmj.com/site/about/guidelines.xhtml

Sample size	14	Estimated number of participants needed to achieve study objectives and how it was determined, including clinical and statistical assumptions supporting any sample size calculations	_____18-19_____
Recruitment	15	Strategies for achieving adequate participant enrolment to reach target sample size	_____13-14_____
**Methods: Assignment of interventions (for controlled trials)**			
Allocation:			
Sequence generation	16a	Method of generating the allocation sequence (eg, computer-generated random numbers), and list of any factors for stratification. To reduce predictability of a random sequence, details of any planned restriction (eg, blocking) should be provided in a separate document that is unavailable to those who enrol participants or assign interventions	_____14______
Allocation concealment mechanism	16b	Mechanism of implementing the allocation sequence (eg, central telephone; sequentially numbered, opaque, sealed envelopes), describing any steps to conceal the sequence until interventions are assigned	_____14______
Implementation	16c	Who will generate the allocation sequence, who will enrol participants, and who will assign participants to interventions	_____13-14___
Blinding (masking)	17a	Who will be blinded after assignment to interventions (eg, trial participants, care providers, outcome assessors, data analysts), and how	_____18______
	17b	If blinded, circumstances under which unblinding is permissible, and procedure for revealing a participant's allocated intervention during the trial	_____18______
**Methods: Data collection, management, and analysis**			
Data collection methods	18a	Plans for assessment and collection of outcome, baseline, and other trial data, including any related processes to promote data quality (eg, duplicate measurements, training of assessors) and a description of study instruments (eg, questionnaires, laboratory tests) along with their reliability and validity, if known. Reference to where data collection forms can be found, if not in the protocol	______26-27___
	18b	Plans to promote participant retention and complete follow-up, including list of any outcome data to be collected for participants who discontinue or deviate from intervention protocols	______19_____

For peer review only - http://bmjopen.bmj.com/site/about/guidelines.xhtml

Data management	19	Plans for data entry, coding, security, and storage, including any related processes to promote data quality (eg, double data entry; range checks for data values). Reference to where details of data management procedures can be found, if not in the protocol	______23_______
Statistical methods	20a	Statistical methods for analysing primary and secondary outcomes. Reference to where other details of the statistical analysis plan can be found, if not in the protocol	______19-20____
	20b	Methods for any additional analyses (eg, subgroup and adjusted analyses)	______19-20____
	20c	Definition of analysis population relating to protocol non-adherence (eg, as randomised analysis), and any statistical methods to handle missing data (eg, multiple imputation)	______19-20____
**Methods: Monitoring**			
Data monitoring	21a	Composition of data monitoring committee (DMC); summary of its role and reporting structure; statement of whether it is independent from the sponsor and competing interests; and reference to where further details about its charter can be found, if not in the protocol. Alternatively, an explanation of why a DMC is not needed	_____N/A______
	21b	Description of any interim analyses and stopping guidelines, including who will have access to these interim results and make the final decision to terminate the trial	_____N/A______
Harms	22	Plans for collecting, assessing, reporting, and managing solicited and spontaneously reported adverse events and other unintended effects of trial interventions or trial conduct	______17______
Auditing	23	Frequency and procedures for auditing trial conduct, if any, and whether the process will be independent from investigators and the sponsor	_____N/A______
**Ethics and dissemination**			
Research ethics approval	24	Plans for seeking research ethics committee/institutional review board (REC/IRB) approval	_____22_______
Protocol amendments	25	Plans for communicating important protocol modifications (eg, changes to eligibility criteria, outcomes, analyses) to relevant parties (eg, investigators, REC/IRBs, trial participants, trial registries, journals, regulators)	____ 23________

For peer review only - http://bmjopen.bmj.com/site/about/guidelines.xhtml

Consent or assent	26a	Who will obtain informed consent or assent from potential trial participants or authorised surrogates, and how (see Item 32)	___13________
	26b	Additional consent provisions for collection and use of participant data and biological specimens in ancillary studies, if applicable	___41__________
Confidentiality	27	How personal information about potential and enrolled participants will be collected, shared, and maintained in order to protect confidentiality before, during, and after the trial	___23_________
Declaration of interests	28	Financial and other competing interests for principal investigators for the overall trial and each study site	___N/A________
Access to data	29	Statement of who will have access to the final trial dataset, and disclosure of contractual agreements that limit such access for investigators	___23_________
Ancillary and post-trial care	30	Provisions, if any, for ancillary and post-trial care, and for compensation to those who suffer harm from trial participation	___N/A________
Dissemination policy	31a	Plans for investigators and sponsor to communicate trial results to participants, healthcare professionals, the public, and other relevant groups (eg, via publication, reporting in results databases, or other data sharing arrangements), including any publication restrictions	___22________
	31b	Authorship eligibility guidelines and any intended use of professional writers	____2________
	31c	Plans, if any, for granting public access to the full protocol, participant-level dataset, and statistical code	___N/A_______
**Appendices**			
Informed consent materials	32	Model consent form and other related documentation given to participants and authorised surrogates	____36-42____
Biological specimens	33	Plans for collection, laboratory evaluation, and storage of biological specimens for genetic or molecular analysis in the current trial and for future use in ancillary studies, if applicable	____N/A_______

*It is strongly recommended that this checklist be read in conjunction with the SPIRIT 2013 Explanation & Elaboration for important clarification on the items. Amendments to the protocol should be tracked and dated. The SPIRIT checklist is copyrighted by the SPIRIT Group under the Creative Commons "Attribution-NonCommercial-NoDerivs 3.0 Unported" license.

For peer review only - http://bmjopen.bmj.com/site/about/guidelines.xhtml

BMJ Open

Sleeping Sound with Autism Spectrum Disorder (ASD): Study protocol for an efficacy randomised control trial of a tailored brief behavioural sleep intervention for autism spectrum disorder.

Journal:	*BMJ Open*
Manuscript ID	bmjopen-2019-029767.R2
Article Type:	Protocol
Date Submitted by the Author:	25-Jul-2019
Complete List of Authors:	Papadopoulos, Nicole; Deakin University<br>Sciberras, Emma; Deakin University; Murdoch Childrens Research Institute<br>Hiscock, Harriet; Murdoch Childrens Research Institute; The Royal Children's Hospital<br>Williams, Katrina; Murdoch Childrens Research Institute; University of Melbourne, Department of Paediatrics<br>McGillivray, Jane; Deakin University<br>Mihalopoulos, Cathrine; Deakin University<br>Engel, Lidia; Deakin University<br>Fuller-Tyszkiewicz, Matthew; Deakin University<br>Bellows, Susannah; Deakin University<br>Marks, Deborah; Deakin University; Royal Children's Hospital Melbourne<br>Howlin, Patricia; University of Sydney, Brain and Mind Centre; King`s College London, Institute of Psychiatry, Psychology and Neuroscience<br>Rinehart, Nicole; Deakin University
**Primary Subject Heading**:	Evidence based practice
Secondary Subject Heading:	Mental health
Keywords:	ASD, Autism Spectrum Disorder, Sleep, Behavioural sleep intervention, RCT

SCHOLARONE™
Manuscripts

Title: Sleeping Sound with Autism Spectrum Disorder (ASD): Study protocol for an efficacy randomised control trial of a tailored brief behavioural sleep intervention for autism spectrum disorder.

Nicole Papadopoulos[1], Emma Sciberras[1,2,3], Harriet Hiscock[2,4], Katrina Williams[2,3,4], Jane McGillivray[1], Cathrine Mihalopoulos[1], Lidia Engel[1], Matthew Fuller-Tyszkiewicz[1], Susannah T Bellows[1], Deborah Marks[1,4], Patricia Howlin[5,6] & Nicole Rinehart[1].

Affiliations:

1. Deakin University, Geelong, Victoria, Australia
2. Murdoch Children's Research Institute, Parkville, Victoria, Australia.
3. Department of Paediatrics, University of Melbourne, Parkville, Victoria, Australia.
4. Royal Children's Hospital, Parkville, Victoria, Australia.
5. Brain and Mind Centre, University of Sydney, Camperdown, New South Wales, Australia.
6. Institute of Psychiatry, Psychology and Neuroscience, King's College London, London, UK.

Corresponding author

Dr Nicole Papadopoulos

Address: Level 4, Building BC. 211 Burwood Highway, Burwood. 3125, VIC.

Telephone: (03) 9244 5295

Email: nicole.papadopoulos@deakin.edu.au

Word count: 5,308

Keywords: autism spectrum disorder; ASD; sleep; behavioural sleep intervention; RCT

For peer review only

ABSTRACT

Introduction: Sleep problems are a characteristic feature of children with autism spectrum disorder (ASD) with 40 to 80% of children experiencing sleep difficulties. Sleep problems have been found to have a pervasive impact on a child's socio-emotional functioning, as well as on parents' psychological functioning. The *Sleeping Sound ASD* project aims to evaluate the efficacy of a brief behavioural sleep intervention in reducing ASD children's sleep problems in a fully powered randomised controlled trial (RCT). Intervention impact on child and family functioning is also assessed.

Methods & analysis: The RCT aims to recruit 234 children with a diagnosis of ASD, aged 5-13 years, who experience moderate to severe sleep problems. Participants are recruited from paediatrician clinics in Victoria, Australia, and via social media. Families interested in the study are screened for eligibility via phone, and then asked to complete a baseline survey online, assessing child sleep problems, and child and family functioning. Participants are then randomised to the intervention group or treatment as usual comparator group. Families in the intervention group attend two face-to-face sessions and a follow-up phone call with a trained clinician, where families are provided with individually tailored behavioural sleep strategies to help manage the child's sleep problems. Teacher reports of sleep, behavioural, and social functioning are collected, and cognitive ability assessed to provide measures blind to treatment group. The primary outcome is children's sleep problems as measured by the Children's Sleep Habits Questionnaire (CSHQ) at 3-months post-randomisation. Secondary outcomes include parent and child quality of life, child social, emotional, behavioural, and cognitive functioning, and parenting stress and parent mental health. Cost-effectiveness of the intervention is also evaluated.

For peer review only

Ethics and dissemination: Findings from this study will be published in peer-reviewed journals and disseminated at national and international conferences, local networks and online.

Registration details: ISRCTN registry-ISRCTN14077107 (3/03/2017)

STRENGTHS AND LIMITATIONS

- This sleep intervention consists of a brief behavioural intervention thus increasing the chances of the intervention being translated into clinical practice.
- This study is the largest randomised control trial to date on the efficacy of the Sleeping Sound program for children with ASD.
- Limitations of the study include: lack of an objective sleep measure, inability to control families' use of additional sleep treatments post-trial enrolment, and lack of gold standard ASD diagnostic tools (e.g. Autism Diagnostic Observation Schedule-Second Edition (ADOS-2), Autism Diagnostic Interview-Revised (ADI-R)) to confirm ASD diagnosis.

For peer review only

Autism spectrum disorder

Autism spectrum disorder (ASD) is a neurodevelopmental disorder characterised by severe deficits in social, communication skills, and restricted and repetitive patterns of behaviour.[1] In 2015, it was estimated that ASD affected approximately 0.7% of the Australian population, which equates to approximately 164,000 Australians.[2] A diagnosis of ASD is associated with major child, familial, and societal costs, with the economic burden in Australia estimated to be between $8.1 and $11.2 billion annually.[3] Psychiatric comorbidity is common in individuals with ASD, with approximately 70% meeting the criteria for another mental disorder,[1] including anxiety (84%),[4, 5] and attention-deficit/hyperactivity disorder (ADHD; 28 to 70%).[6, 7] Sleep problems are also common in children with ASD and cause an additional burden for children and their families. However, large rigorous randomised controlled trials (RCTs) that report on the efficacy of treating sleep problems in this population are lacking.[8]

Sleep problems in children with ASD - prevalence, aetiology and burden

It is estimated 40-80% of children with ASD experience sleep problems,[9-13] with sleep onset delay, shorter sleep duration, and frequent prolonged night waking being particularly common.[9, 13-18] Other common sleep problems include bedtime resistance, sleep anxiety, daytime sleepiness, early waking, co-sleeping, low sleep efficiency, and parasomnias.[9, 13, 16-20] Children with ASD will also often experience more than one sleep problem concurrently.[16] Furthermore, sleep problems in children with ASD have been shown to be more persistent over time compared to sleep problems in typically developing children.[10-12, 21, 22]

The aetiology of sleep problems in children with ASD is unclear and likely multifactorial,[12, 20] although various biopsychosocial factors have been proposed.[13, 23, 24] Biological factors that may impact sleep in ASD include abnormalities in melatonin

For peer review only

secretion, brain wave organizational and maturational differences, circadian-relevant genes, and arousal and sensory dysregulation.[13, 20, 25] Social-emotional and behavioural characteristics connected with core ASD features may also contribute to sleep difficulties.[13, 25]. Children with ASD often have difficulty recognizing contextual and environmental cues that indicate that bedtime is approaching, and may have difficulty settling if bedtime routines are not able to be fulfilled.[13, 25] Co-occurring psychological factors such as anxiety, depression, and ADHD, as well as family factors including parental stress, parental mental health, and home environment may also influence sleep problems.[11, 13, 20, 26]

Several studies have linked sleep problems with behavioural difficulties and psychopathology in ASD. In a large study conducted by Goldman et al.[27] (*n* = 1,784), children with ASD who were poor sleepers were found to have more behavioural problems, including problems with attention and social interactions compared to good sleepers (77.7% versus 56.1%, and 75.4% versus 53.9% respectively). Sleep problems in children with ASD have also been associated with thinking difficulties;[28] increased communication and social deficits and higher rates of stereotypic behaviours;[29, 30] and with anxiety and mood disturbances.[31] In addition, overall severity of sleep problems has been found to be associated with poorer child physical and psychosocial health related quality of life,[32] and cognitive and motor functioning.[33]

Sleep problems have also been identified as having a pervasive impact on the family, including an elevated risk for parenting stress. For example, several studies have identified moderate to small associations between parenting stress and sleep problems in children with ASD.[23, 34-37] Two studies have linked sleep problems in children with ASD with poor parent mental health.[36, 38] One study found a moderate association between child sleep

For peer review only

problems and maternal mental health;[36] a second reported a small-to-medium association between child sleep problems and parent depression.[38]

Treating sleep problems in children with ASD

Both behavioural and pharmacological interventions are commonly prescribed to treat sleep problems in ASD; however, we have much to learn about the efficacy of these treatment approaches.[8] For some sleep disorders, behavioural sleep interventions are recommended as the first line of treatment. Gingras et al.[39] conducted one of the largest melatonin sleep trials involving 146 children with neurodevelopmental disabilities (including ASD). Prior to randomisation for melatonin treatment, Gingras et al.[39] provided families with a booklet outlining good sleep habits and behavioural sleep strategies. Following administration of sleep strategies for a one-month period, 35% of the sample no longer met inclusion criteria for the trial. Whilst this study did not solely focus on children with ASD, the results indicate the potential of a brief behavioural sleep intervention for children with ASD.

Despite the promising evidence to support the use of behavioural sleep interventions for children with ASD, few studies have examined their efficacy.[8, 40] A recent systematic review of behavioural sleep interventions to treat insomnia in children with neurodevelopmental disorders identified 19 ASD studies; only three studies employed an RCT design.[41] Findings of the three studies are mixed. Adkins et al.[42] (n = 36) found no effect of a standardised information pamphlet on insomnia compared to a control (no intervention) group. The findings suggest that psychoeducation alone is insufficient to produce behavioural change, and that specific, tailored examples may be more useful for parents when addressing child sleep problems.

Johnson et al.[43] undertook a pilot RCT (n = 40) comparing a five-session individualised parent behavioural sleep training program with a control group (parent

For peer review only

psychoeducation autism program without a behavioural sleep focus). The behavioural sleep training program involved parents receiving information about how to treat behavioural sleep problems often observed in children with ASD, as well as ways of addressing future sleep issues that are typical in ASD. Families allocated to the behavioural sleep training program reported significant improvements in sleep problems (effects small to medium) as measured by the composite sleep index of the modified version of the Simonds and Parraga sleep questionnaire.[44, 45] However, no benefits were identified using more objective measures (e.g. actigraphy), and the authors did not report any improvement in child and parent outcomes despite parent satisfaction of the manualised program being high.

Malow et al.[46] assessed the efficacy of a parent sleep education program on sleep onset delay in children with ASD (n = 80), in individual versus group (2-4 people) settings. Both delivery methods were effective in reducing sleep onset delay, with large effect. Improvement was also seen in: bedtime resistance and sleep duration, with medium-to-large effect; and, sleep anxiety and night waking, with small-to-medium effect. Improvements in child and family functioning were also reported, including increased child quality of life and parental sense of competence, each with small-to-medium effect. However, findings are limited by the lack of a treatment as usual (TAU) control group.

More recently, Hiscock et al.[47] showed that a brief two-session parent/child intervention, *Sleeping Sound,* was highly efficacious in reducing behavioural sleep problems in children with ADHD when compared to a TAU control group. The *Sleeping Sound* intervention was tailored to the family, and included behavioural strategies targeted at the child's specific sleep problems. Based on this, we examined the preliminary efficacy of the *Sleeping Sound* program in 61 children with comorbid ASD-ADHD.[48] In comparison to children with ASD-ADHD receiving TAU (n = 33), children with ASD-ADHD (n = 28) who received the *Sleeping Sound* intervention showed large improvements in child sleep

For peer review only

problems, as measured by parent report of reduced total scores on the CSHQ[49] at 3-months post-randomisation (medium-to-large effect), and 6-months post-randomisation (medium effect). The intervention group also showed greater improvements in psychosocial functioning at 3- and 6-month follow-up, with small-to-medium, and medium effect respectively. Children in the intervention group were further described as showing improved emotional functioning at 3-months by parent-report, with medium-to-large effect, and teacher-report, with medium effect.

It appears that a tailored behavioural sleep intervention may improve sleep in children with ASD; however, few rigorous RCTs have been conducted.[8, 40] Further, it is unclear whether behavioural sleep interventions for children with ASD lead to improvements in other domains of functioning, such as behavioural and emotional problems, social communication, parenting stress, and parent mental health. Given that our pilot findings demonstrated that a tailored brief behavioural sleep intervention for children with ASD-ADHD can improve not only sleep, but also emotional, behavioural and social functioning, we have selected this intervention to test in a fully powered RCT.

Aims and hypotheses

This study aims to examine the efficacy of a brief behavioural sleep intervention *(Sleeping Sound*) in treating sleep problems in primary school children aged 5-13 years with ASD compared to a TAU group. We have chosen a TAU control group for this trial to allow us to investigate the efficacy of the Sleeping Sound intervention for children with autism who may already be receiving community or clinical interventions.

We aim to assess whether the brief sleep intervention delivered by study clinicians is related to the following outcomes:

For peer review only

Primary Outcome

1. Reductions in overall child sleep problem severity at 3-months post-randomisation, including reductions in: total sleep disturbances, problems initiating sleep, and problems maintaining sleep.

Secondary Outcomes

2. Improvements in child and primary parent functioning at 3- and 6-months post-randomisation.

We also aim to assess whether the brief sleep intervention is cost-effective when compared with TAU.

We hypothesize that compared with a TAU comparison group, children with ASD who receive the *Sleeping Sound* intervention will show greater improvements at 3-months post-randomisation (post-intervention; primary outcome time point) and 6-months post-randomisation in the following areas:

1. Reduction in overall child sleep problems
2. a. Decreased daytime sleepiness by teacher report, and improvements in sleep hygiene and sleep disturbances by parent report;

   b. Decreased emotional and behavioural problems reported by parents and teachers;

   c. Decreased social-communicative symptoms reported by parents;

   d. Increased cognitive performance, academic achievement and school attendance;

   e. Increased child quality of life reported by parents;

   f. Decreased parent stress and mental health symptoms; and,

   g. Increased parent quality of life and work attendance.

We also hypothesize that the behavioural sleep intervention will be a cost-effective treatment when compared with TAU from a societal and healthcare perspective. Thus, we will compare the costs and benefits (expressed in quality-adjusted life years (QALYs))

For peer review only

between the two arms of the trial over the period of the study (further details are provided below).

METHODS AND ANALYSIS

Overall study design

The *Sleeping Sound with ASD* study is an RCT where participants are allocated either to the sleep intervention group or a TAU group. The project will run from July 2016 to the end of 2019 when all stages of the project, from recruitment to follow-up data collection at 6-months post-randomisation, will be executed. Participant recruitment concluded in March 2019; the collection of 3- and 6-month data is ongoing. This study protocol conforms with the SPIRIT reporting requirements,[50] and a SPIRIT timeline has been provided (Figure 1).

(Insert Figure 1)

Participants

Participants include Victorian families of children with ASD, aged 5 to 13 years, and attending primary school at the time of recruitment. All children have a parent-reported moderate to severe sleep problem of chronic insomnia and/or delayed sleep-wake phase as defined by the International Classification for Sleep Disorders – Third Edition.[51] The intervention is designed to be feasibly delivered within the context of the Australian healthcare system; therefore, eligibility for an ASD diagnosis is based on the nationally agreed DSM-IV (Autistic Disorder, Asperger’s Disorder),[52] or DSM-5 (ASD) assessment criteria.[1] All children have a diagnosis of ASD which has been confirmed by consensus of diagnosis by a multidisciplinary team specialised in ASD assessment (psychologist, paediatrician/psychiatrist, speech pathology and/or occupational therapist). Written evidence of a conclusive multidisciplinary diagnosis of ASD is sighted, or confirmed by the treating paediatrician for all children enrolled in the study. Children also met clinical ASD

For peer review only

symptomology based on a clinical cut off score of ≥ 11 on the Social Communication Questionnaire Lifetime form.[53]

Patient involvement

The intervention used in this study is based on the ADHD *Sleeping Sound* trial, during which participant feedback on the acceptability and feasibility of the study was collected.[47] There were no reported concerns from participants.[47] In this trial we will be collecting feedback data about the program from parents.

Recruitment: stage 1

Recruitment through Victorian paediatric clinics. Victorian paediatricians identified potential participants meeting inclusion criteria, whom they have seen in the last 12 months, and sent a letter inviting them to participate in the study. Study advertisements were also displayed in the waiting areas of clinical services, and disseminated widely through research, clinical, and community-based networks available to investigators.

Recruitment: stage 2

The research team telephoned all primary parents who registered interest in the study to assess inclusion/exclusion criteria (approximately 25-minute phone call). Eligible families were provided with an information sheet, consent form (see Appendix), and baseline survey by email or post. The child's primary parent was asked to complete the survey and consent form. Online surveys were completed via a secure research database REDCap.[54] Once parents provided written consent, they were asked to provide diagnostic and cognitive assessment reports where available. If optional consent was provided to contact the child's school teacher, a link to an online survey was sent to the child's nominated teacher.

Families are sent a separate hardcopy consent form for researchers to access the child's Medicare Benefits Scheme (MBS) and Pharmaceutical Benefits Scheme (PBS) data for cost-effectiveness analysis. Medicare collects information on the child's doctors' and allied health

For peer review only

professionals' visits and the associated costs (including out-of-pocket costs). PBS collects information on prescription medications which have been recorded at pharmacies.

Participants are required to meet all inclusion criteria (see Table 1). Children currently prescribed melatonin as well as any other medications (e.g. SSRIs, etc.) are included in the trial if they continue to meet eligibility criteria. Information regarding medication is collected as part of the parent survey at time of enrolment and at 3- and 6-months follow-up. We have included a table documenting important changes to our inclusion/exclusion criteria after trial commencement. (See Table 2).

(Insert Table 1 and Table 2 here)

Randomisation

The flow of participants from recruitment to follow-up assessments is displayed in Figure 2. Participants are randomised to either the intervention or TAU groups by an independent research assistant once consent forms and baseline surveys have been completed. Treatment allocation is determined sequentially, according to a computer-generated block randomisation sequence with 1:1 ratio between groups, with blocks of randomly varying size (4, 6 and 8) developed by an independent statistician who is not involved in the project. The independent statistician will then upload this randomisation list into REDCap,[54] a secure research database, to ensure allocation concealment. Given the relatively fewer diagnoses of ASD among girls,[55] randomisation is stratified by gender to ensure equal representation of girls, boys and other genders (i.e. transgender, intersex) in both study arms. Siblings are eligible to participate in the study provided they each meet all inclusion criteria. Families with siblings enrolled in the study are randomised in sequence based on the return of the primary parent baseline surveys, with all subsequent siblings allocated to the same treatment group. All families are sent a letter informing them of their

For peer review only

group allocation and are not able to enrol further siblings once notified of group allocation. A study clinician then telephones intervention families to book the intervention sessions.

The TAU group will receive standard services and support currently available in the community and complete the same study assessment follow-up. If the parent consented to teacher participation, researchers approach schools for consent to contact the child's teacher and then mail the teacher a baseline survey to complete.

(Insert Figure 2 here)

Intervention group

Delivery of the sleep intervention. The behavioural sleep intervention is delivered by study-employed clinicians (paediatrician or psychologist experienced in working with children with ASD). Clinicians were trained over two x 3-hour interactive sessions (by HH and ES), have a detailed program manual, and meet fortnightly to ensure fidelity to the program. The intervention consists of two 50-minute face-to-face consultations and one follow-up phone call at two-week intervals. The content of the original *Sleeping Sound* ADHD intervention,[47] has been adapted to children with ASD to include specific visual aids and social stories to assist in the delivery of the sleep intervention for this population.[56] Intervention sessions are held in rooms at one of our study sites (Deakin University or The Royal Children's Hospital), or the treating paediatrician's consulting rooms, based on family preference.

The behavioural sleep interventions are individually tailored for the child and the family. The strategies that suit the family situation and learning style of the child with ASD are given emphasis. Delayed sleep phase, early morning waking and prolonged night waking (e.g. waking overnight where the child is unable to fall back to sleep for an hour or more) are treated with bedtime fading (i.e. the child's bedtime is temporarily set later and gradually brought forward), as well as early morning light exposure. Need for parental presence at

For peer review only

sleep time is managed for sleep onset association using a ‘camping out’ method. Camping out involves parents gradually withdrawing themselves from the child’s bedroom over a period of time. Parent management strategies are used for bedtime resistance and can include use of a ‘bedtime pass’, whereby the child can only leave the bedroom once before sleep, and may be coupled with a reward system. Primary insomnia (insomnia with no other established cause) is managed using relaxation training and restricting time in bed (i.e. bedtime fading or getting out of bed and doing a relaxing activity if the child cannot fall asleep). In addition to relaxation training, examples of some strategies used to manage anxiety-related insomnia include discussing fears during the day rather than just before bedtime, encouraging the child to stay in bed, and rewarding brave behaviour. Visual strategies, such as social scripts and visual timetables are used to reinforce learning.

Session One focuses on an assessment of the child’s sleep problems, psychoeducation about normal sleep and sleep cycles, good sleep habits, and a tailored sleep management plan specific to the sleep diagnosis of the child. As the Sleeping Sound intervention is individually tailored to children and families, participants are encouraged to provide input in relation to goal setting and the interventions they would like to try to assist in managing the sleep problems. Families receive written handouts, summarising session content and complete a written management plan with the clinician. A standardised consultation record is kept for all children including the presenting sleep problem(s), possible contributors to the problem (e.g. TV in bedroom), medication use, comorbidities and bedtime routines. Clinicians record the duration of each consultation, sleep problem diagnoses, family sleep management goals, handouts given to parents, and management options selected. Sleep problem diagnoses made may include one or more difficulties getting to sleep (sleep anxiety, bedtime resistance, sleep association onset disorder, delayed sleep phase, or insomnia), and/or night waking problems (prolonged night waking or early morning

For peer review only

waking). Parasomnias (nightmares, night terrors, sleep walking) are recorded, and psycho-education and general management advice about parasomnias is provided, although these are not a focus of behavioural intervention.

Session Two is held two weeks later to review the sleep management plan (which includes a sleep diary), reinforce strategies, trouble-shoot, and monitor progress.

A *follow-up phone call* is conducted two weeks after the second visit to answer any further parent questions, reinforce strategies, trouble-shoot, and monitor progress. Participants are able to withdraw from the intervention at any time. Participants are asked for feedback of the intervention at all intervention sessions, including follow-up, and this is documented by clinicians in order to manage any unintended adverse effects of the trial.

Measures

Study measures are summarised in Table 3 below. Additional information collected at baseline includes family and child demographics such as socio-economic status (SES) and medical data (i.e. diagnosed comorbidities etc.). Medication information is captured at baseline and 3- and 6-months follow-up. Based on prior work with children with ADHD and ASD we do not anticipate any risks, side-effects or discomfort to participants in completing our measures. In the event that a parent becomes distressed when completing questionnaires related to stress or mental health we have included information to link participants to a psychologist on our research team as well as directing parents to contact their General Practitioner.

We have chosen the CSHQ as our primary outcome measure as this is a widely used and validated measure of behavioural sleep problems in children,[49] and was used in our pilot *Sleeping Sound* ADHD-ASD study.[48] It has been used to examine sleep behaviour in children with ASD and correlates highly with the objective measure of actigraphy.[57] It contains 33 items and includes eight subscales: Bedtime Resistance (e.g. *Child falls asleep in*

For peer review only

own bed), Sleep Onset Delay (e.g. *Child falls asleep in 20 minutes after going to bed*), Sleep Duration (e.g. *Child sleeps the right amount*), Sleep Anxiety (e.g. *Child needs parent in the room to fall asleep*), Night Waking (e.g. *Child moves to someone else's bed during the night*), Parasomnias (e.g. *Child talks during sleep*), Sleep Disordered Breathing (e.g. *Child snores loudly*), and Daytime Sleepiness (e.g. *Child has difficulty getting out of bed in the morning*). A 3-point rating scale from 1 = *rarely* to *3* = *usually* is applied to each item, indicating the sleep problem frequency during the past week. Some items are reverse coded and higher scores are indicative of more sleep problems. The measure has good internal consistency (α = .78) and each subscale also has adequate internal consistency (α = .93 - .56).[49]

Knowledge of participant group status will be limited to the project coordinator and study clinicians. If a family spontaneously reveals their intervention status to another team member this is noted on the participant file. Given that it is impossible to maintain participant blinding in a trial of this nature, we have carefully chosen some outcome measures that can be assessed in a blinded fashion. For example, we will include a blinded teacher-reported measure of sleep, behavioural, social and motor functioning. We will not inform teachers of the child's group status and will ask parents not to inform teachers. We will also include blinded objective child measures of executive and school-based functioning.

(Insert Table 3 here)

Sample size

In our pilot study,[48] we found a moderate-to-large effect of the intervention on sleep for total CSHQ score (standardised mean difference = 0.7). However, since effect sizes from small studies may not be replicated in larger studies, we conservatively estimate a moderate standardised mean difference of 0.5 at 3- and 6-months follow-up. Required sample size for this standardised mean difference was calculated using the equation $n = 2\ (Z\alpha + Z\ [1-\beta])^2 \times SD^2 / d^2$, where Z_α was set at $p < .05$ (two-tailed), $Z_{1-\beta}$ (power) was set at .80, d was set

at .5 as per above, and the standard deviation for this standardised mean difference was set at 1. The resulting sample size estimate was then adjusted for the impacts of attrition (allowing for 20% loss to follow up) and clustering (by individual and paediatrician). Clustering was handled with adjustments for design effect, assuming intra-class correlation (ICC) = .08 for the within child's paediatrician clustering (average cluster size = 4, SD = 3), and ICC = .20 for the within person clustering to control for repeated measurement due to control of baseline scores (estimated based on unpublished pilot data).[58] Allowing for 20% attrition over time and these forms of clustering, we will randomise a minimum of 234 children (117 in each group) to ensure sufficient power post-intervention with our primary outcome timepoint at 3-months post-randomisation. Full calculations are available in the Supplementary File.

Strategies have been put in place to encourage participant retention and the completion of the follow-ups. These include a 4-step reminder process for completing follow-up assessments and a study newsletter to promote family engagement.

Data analysis

Primary analysis will be intention-to-treat (within participant data included as per initial treatment group allocation, regardless of actual intervention received) and will compare outcomes of children in the TAU and intervention groups at the 3- and 6-month post-randomisation time points, though our primary endpoint is 3-months post-randomisation. Analysis of the primary outcome (i.e. child sleep problems) and all secondary outcomes (child – teacher reported daytime sleepiness, sleep hygiene, emotional and behavioural problems, social-communicative symptoms, cognitive performance, academic achievement, school attendance and quality of life; parent – parenting stress, mental health, quality of life and work attendance) will be carried out using linear mixed effects regression with results presented as mean differences (and 95% CIs). For each outcome, a single mixed effects

For peer review only

model will be fitted incorporating baseline, 3-month, and 6-month data using random effects to allow for the repeated measures within an individual, and clustering of individuals within the child's paediatrician. Separate parameters will be estimated for group differences at 3- and 6-months post-intervention; scores at baseline are added as a covariate. Differences in the outcomes of intervention and TAU groups will be assessed with linear mixed models, with individuals clustered within the child's paediatrician and assessment times clustered within individuals. We will run an unadjusted model and compare this to an adjusted model, with the unadjusted model as our primary analysis, and adjusted results to evaluate robustness of these findings. The adjusted model will include the following as covariates: child gender, age, ASD symptom severity (measured using the Social Communications Questionnaire – current form,[53] medication use (obtained from the Resource Used Questionnaire) and socioeconomic status (measured according to the families residential postal code and using the index of relative socioeconomic disadvantage.[47, 59] The child's paediatrician and individual participant will be modelled as random effects and the remaining variables will be handled as fixed effects. To account for missing data, the mixed models will be fitted using conditional maximum likelihood. As this approach makes an untestable assumption that data are missing at random, we will perform sensitivity analyses in the form of pattern mixture models to explore the effect of departure from this assumption. A per-protocol analysis will also be undertaken as a supplementary analysis. Significance will be tested at $p < .05$ for the primary analysis, but adjustments for Type I error inflation will be made for secondary outcomes by using the False Discovery Rate method.[60]

The number of siblings in the trial is expected to be low based on our prior work. Even if the proposed mixed effects model were able to run with family as a clustering factor despite low number of sibling pairs, there is still some concern about obtained parameter estimates.[61] As such, alternative modelling approaches which can handle sparse data (such

For peer review only

as generalised estimating equations) will be used as a sensitivity analysis to evaluate impact of clustering due to presence of siblings on results obtained from our model without this correction.[61]

Economic evaluation

An important secondary aim of the project will be to determine the cost-effectiveness of this intervention compared to TAU. The inclusion of the CHU9D at baseline, 3-month, and 6-month follow-up will allow calculation of quality-adjusted life years (QALYs) as part of a cost-utility analysis, applying the area-under-the-curve approach.[62] A supplementary cost-effectiveness analysis will also be undertaken using the total CSHQ score as an outcome measure. The economic analysis will be conducted from a societal perspective, since interventions targeting children with ASD are likely to have benefits and costs beyond the narrow health sector (e.g. ASD intervention services and productivity impacts for parents). A secondary analysis from a healthcare perspective will also be undertaken. The evaluation will first measure and value the use of healthcare resources over the period of the study between the two arms of the trial and then compare any additional costs to the additional outcomes achieved. Costs of the intervention will be measured using the financial records of the study team. Other costs incurred by children and their families will be measured using a resource use questionnaire. Components of the resource use questionnaire comprise the child's use of healthcare services, including: (i) inpatient services and emergency services, (ii) health professional visits, (iii) medication, and (iv) other resources used. The questionnaire also collects data on out-of-pocket and travel costs, as well as a child's school attendance and parent's productivity. Participants will also be asked for their permission to access their MBS and PBS data for a time period of 3 months prior to baseline up until the last follow-up assessment. These data are not subject to recall bias and provide complete resource use costs (and out of pocket costs) for all Medicare and PBS reimbursed healthcare

For peer review only

services. Non-Medicare and non-PBS funded services (e.g. state-funded ASD services) will be captured in the resource use questionnaire. Generalised linear models will be used to assess mean difference in costs and outcomes between the two arms, adjusted for any differences between groups at baseline. An incremental cost-effectiveness ratio (ICER) and an incremental cost-utility ratio (ICUR) will be calculated as the difference in average cost between the groups, divided by the difference in average CSHQ and QALYs respectively. Non-parametric bootstrapping will be undertaken from the observed cost/QALY pairs (1,000 simulated replications) to determine CIs and presented in a cost-effectiveness plane along with a cost-effectiveness acceptability curve. Sensitivity analyses will be carried out to explore the robustness of the results. While MBS and PBS data will be used for the base case analysis, a sensitivity analysis will be performed using self-report data derived from the resource use questionnaire for those health services captured by MBS and PBS.

ETHICS AND DISSEMINATION

Behavioural sleep problems are a common burden associated with ASD. The proposed RCT of the *Sleeping Sound* intervention in ASD children with sleep problems will result in a significant advance in research on the treatment of sleep problems. The study is well-justified with reference to the demonstrated success of this tailored sleep intervention in ADHD,[47] our strong pilot data,[48] and the potential advantages of the intervention in alleviating social, emotional and academic problems connected to the sleep disorder in the children themselves and their parents. If the intervention is found to be effective in this sample of children who are of borderline/average intellectual ability we will subsequently be able to explore whether the intervention is successful for children with ASD who also have an intellectual disability. Treatment recommendations for treating sleep problems in children with ASD have been released.[63] The Sleep Committee of the Autism Treatment Network recommend the use of behavioural intervention as a first line therapy.[64] The Autism UK NICE clinical guidelines

For peer review only

(#170) for sleep management recommend development of individualised behavioural intervention which includes specialist monitoring, and use of a sleep plan as a central concept of sleep management.[65] To date, the treatment of sleep disturbance in children with ASD is based on expert opinion and consensus practise, informed by limited evidence. Results from this study will inform evidence based clinical practice and guide appropriate selection and tailoring of behavioural strategies to address specific sleep problems. We aim to publish our findings in peer-reviewed journals and present our research at international conferences and community organisations. The study has been granted ethical approval by the Royal Children's Hospital (#36154), Deakin University (#2017-130), the Victorian Department of Education and Early Childhood Development (#2016_003134) and the Catholic Education Office (Melbourne #0501) Human Research Ethics Committees. Important protocol changes are reported to each ethics committee via ethics amendments.

DATA MANAGEMENT

All participant data will be confidential and hardcopy data will be securely stored in locked offices for which only research team members have access to the keys. All online participant data are collected and stored in REDCap,[54] which is a secure database stored on the Deakin University network, accessible only to the research team via individual usernames and passwords. All chief investigators will have access to the final dataset. Information will be kept for seven years after the youngest participant's 18th birthday or 15 years after the completion of the study, whichever date is later. After this time the data will be destroyed.

Data from all hardcopy surveys will be entered into REDCap by a member of the research team.[54] To ensure quality data a process of data checking and cleaning will be undertaken by the research team. We will keep a register of data issues to ensure problems with data are addressed in a timely manner and conduct regular audits of data management processes.

For peer review only - http://bmjopen.bmj.com/site/about/guidelines.xhtml

Table 1
Study Inclusion and Exclusion Criteria

Inclusion criteria	
	Clinically confirmed diagnosis of DSM-IV Autistic Disorder,[52] Asperger's Disorder or DSM-5 ASD.[1] Diagnosis has been confirmed by consensus of a multidisciplinary team, as indicated by paediatrician or a confirmed multidisciplinary diagnosis as cited in a clinical report.
	Aged 5-12 years, or 13 years and attending primary school at the time of recruitment.
	Child meets clinical ASD symptomology based on a clinical cut off score of ≥ 11 on the Social Communication Questionnaire Lifetime form (40-item questionnaire measuring ASD symptoms).[53]
	Child's sleep problem(s) are moderate or severe by primary parent report and have been a problem for them over the past 4 weeks.[66]
	Meet diagnostic criteria for at least one of the following sleep problems as defined by the International Classification of Sleep disorders diagnostic criteria:[51] chronic insomnia (including problems initiating and maintaining sleep, early morning waking, sleep-onset association, bed-time resistance, and anxiety-related insomnia), or delayed sleep-wake phase.
Exclusion criteria	
	Diagnosed with an intellectual disability by parent report.
	Have any comorbid medical (e.g. nocturnal seizures, blindness) conditions that disturb regular sleep patterns, or genetic (e.g. Fragile X disorder) conditions associated with intellectual impairment based on parent report.
	Have suspected obstructive sleep apnoea (OSA). OSA is assessed using 3 OSA items from the CSHQ.[49] This scale can help identify children who may suffer from OSA. If a parent endorses items indicative of OSA, the child is contacted by the study paediatrician for further assessment. If OSA is suspected, the child is excluded from the research study and referred to appropriate clinical services. After approximately six months the family is followed up to see whether they are eligible to participate in the study (i.e. if OSA symptoms have resolved and they have ongoing behaviour sleep problems).
	Parents with insufficient English language proficiency to provide informed consent, complete study materials and/or participate in the intervention treatment program.

For peer review only

Table 2
Amendments to study protocol

'Opt out' study invitation amended to 'opt in' invitation	An 'opt out' approach was initially used to recruit through paediatricians. Parents were sent a letter from the paediatrician advising them that the research team would call them to explain the study, confirm eligibility, and invite them into the study. Parents were asked to contact the study team or their paediatrician if they *did not* wish to learn more about the study or be contacted by the research team. The paediatrician then provided the research team with the contact details of families if they did not opt out within the two-week period specified in the letter. This recruitment method was amended to an 'opt in' study invitation letter from paediatricians.
Age range amended	Age range extended to include 13-year olds who attend primary school at the time of recruitment
Social Communication Questionnaire Lifetime form cut off adjusted	Clinical cut off score lowered from ≥15 to ≥11. This measure is used to assess ASD symptomology. There is evidence in the literature to support a lower cut off score in order to capture children with higher-functioning ASD and Asperger's Disorder who may be missed by the use of a more stringent cut off score.[67]
Vineland inclusion criteria removed	The Vineland Adaptive Behaviour Scales 3rd Edition (Domain Interview Survey) was originally conducted with families over the phone as a screening tool for intellectual impairment. This was proving to be too cumbersome for families and a barrier to enrolment. It was therefore removed as a requirement for eligibility.
Comorbid medical conditions exclusion criteria amended	Exclusion criteria for comorbid conditions refined to medical conditions affecting sleep or associated with intellectual disability. Original criteria comorbid medical (e.g. epilepsy), neuropsychiatric (e.g. Tourette's) or syndromic genetic (e.g. Fragile X) conditions

For peer review only

Table 3
Summary of measures included in the study

Outcomes	Measure	Source	B	3	6
**Child outcomes**					
**Sleep problems**	**Children's Sleep Habits Questionnaire (CSHQ).** 33-item validated measure of sleep that can distinguish clinical from community samples.[49] Provides a measure of total sleep problems and eight subscale scores reflecting major behavioural sleep disorders (bedtime resistance, sleep onset delay, sleep duration, sleep anxiety, night waking, parasomnias, sleep disordered breathing, daytime sleepiness).	Parent	●	●	●
**Sleep hygiene**	**Sleep Hygiene Scale.** 7-item study developed measure adapted from the Bedroom Routines Scale.	Parent	●	●	●
**Daytime Sleepiness**	**Teacher Daytime Sleepiness Questionnaire.** 10-item validated scale of daytime sleepiness at school.[68]	Teacher	●	●	●
**Social-Communication functioning**	**Social Communication Questionnaire (SCQ) Current** form. 40-item measure of ASD symptoms in past 3 months to measure change in ASD social-communication symptoms over time.[53]	Parent	●	●	●
**Emotional and behavioural disturbance**	**Developmental Behavioural Checklist (DBC).** 96-item measure of emotional and behavioural disturbance in children.[69] Provides a rating of overall behavioural disturbance and five subscales: disruptive/antisocial behaviour, self-absorbed, communication disturbance, anxiety and ASD social relating.	Parent	●		●
**Behavioural and social functioning**	**Strengths and Difficulties Questionnaire (SDQ).** 25 items assessing the following subscales: hyperactivity/inattention, conduct problems, emotional symptoms, peer relationship problems, and prosocial behaviour.[70]	Parent & Teacher	●	●	●
**Quality of life**	**Child Health Utility 9D (CHU9D).** 9-item measure of child quality of life.[62]	Parent	●	●	●
**School attendance**	**School attendance** over the preceding 3 months.	Parent	●	●	●
**Academic functioning**	**Wide Range Achievement Test 4 (WRAT).** Spelling and math computation subtests to assess academic functioning.[71]	Child			●
**Academic functioning**	Permission to access **NAPLAN** results (Years 3, 5, 7). NAPLAN is a national assessment of literacy and numeracy Australian children complete in Grades 3, 5, 7 and 9.	Linkage			
**Cognitive functioning**	**NIH Toolbox** – Cognitive Domain Tasks to assess cognitive functioning.[72] iPad administered assessment of executive function, attention, episodic/working memory, processing speed, language abilities, new learning and reading.	Child			●
**Parent outcomes**					
**Mental health**	**Kessler 10 (K10)**. A 10-item validated measure of adult psychological distress.[73]	Parent	●	●	●
**Parenting stress**	**Parenting Stress Index 4SF (PSI-4SF).** 36-item measure of parenting stress.[74] Provides a measure of total parenting stress and three subscales reflecting the major sources of parenting stress (parental distress, difficult child, parent-child dysfunctional interaction).	Parent	●	●	●
**Quality of life**	**Assessment of Quality of Life (AQoL4D)**. 12-item measure of parent quality of life.[75]	Parent	●	●	●

**Economic outcomes**					
**Resource use**	Family reported **service use** over preceding 3 months	Parent	●	●	●
**Health service utilisation**	Permission to access both **Medicare and Pharmaceutical Benefits Scheme (PBS)** data for 3 months prior to baseline up until the last follow-up assessment.	Linkage	●	●	●

● measure utilised at indicated timepoint.
B, baseline; 3, 3 months; 6, 6 months

For peer review only

Author contributions

Nicole Rinehart, Nicole Papadopoulos, Emma Sciberras, Harriet Hiscock, Katrina Williams, Jane McGillivray, Pat Howlin and Cathrine Mihalopoulos conceived the study. Susannah Bellows, Deborah Marks and Lidia Engel contributed to the writing of the methods and intervention for the study. Nicole Papadopoulos and Susannah Bellows drafted the manuscript. Matthew Fuller-Tyszkiewicz contributed to the design and analytic components of the study. All authors contributed, read and approved the final manuscript.

Funding

This work was supported by the Australian National Health and Medical Research Council (NHMRC; project grant number APP1101989). Associate Professor Sciberras is funded by an NHMRC Career Development Fellowship 1110688 (2016-21) and a veski Inspiring Women's Fellowship. Harriet Hiscock is supported by an NHMRC Practitioner fellowship 1136222 (2018-2022). The MCRI is supported by the Victorian Government's Operational Infrastructure Support Program.

Competing interests

Professor Rinehart and Professor McGillivray receive funding from the Moose Foundation and the Australian Football League to conduct research in the field of neurodevelopmental disorders and inclusion. Professor Rinehart, Professor McGillivray and Dr Papadopoulos also received funding from the Ferrero Group Australia as part of its Kinder + Sport pillar of Corporate Social Responsibility initiatives to promote active lifestyles among young people. Professor Rinehart has previously received speaker honorarium from Novartis (2002), Pfzier (2006) and Nutricia (2007) and is a Director of the Amaze Board (Autism Victoria). None of the companies or organisational bodies listed above had a role in this research including the

For peer review only

collection, analysis, and interpretation of data; in writing of the manuscript; and /or in the decision to submit the article for publication.

Exclusive license

I, the Submitting Author have the right to grant and does grant on behalf of all authors of the Work (as defined in the below author license), an exclusive license and/or a non-exclusive license for contributions from authors who are: i) UK Crown employees; ii) where BMJ has agreed a CC-BY license shall apply, and/or iii) in accordance with the terms applicable for US Federal Government officers or employees acting as part of their official duties; on a worldwide, perpetual, irrevocable, royalty-free basis to BMJ Publishing Group Ltd ("BMJ") its licensees and where the relevant Journal is co-owned by BMJ to the co-owners of the Journal, to publish the Work in BMJ Open and any other BMJ products and to exploit all rights, as set out in our license.

The Submitting Author accepts and understands that any supply made under these terms is made by BMJ to the Submitting Author unless you are acting as an employee on behalf of your employer or a postgraduate student of an affiliated institution which is paying any applicable article publishing charge ("APC") for Open Access articles. Where the Submitting Author wishes to make the Work available on an Open Access basis (and intends to pay the relevant APC), the terms of reuse of such Open Access shall be governed by a Creative Commons license – details of these licenses and which Creative Commons license will apply to this Work are set out in our license referred to above.

For peer review only

Figure legend

Figure 1. Study timeline, figure format obtained from the SPIRIT 2013 statement.

Figure 2. Flow of participants, figure format obtained from the CONSORT 2010 statement.

References

1. American Psychiatric Association. Diagnostic and Statistical Manual of Mental Disorders. 5th ed. Arlington, VA: APA 2013.
2. Australian Institute of Health and Welfare. Autism in Australia [Internet]. Canberra: AIHW; 2017. https://www.aihw.gov.au/reports/disability/autism-in-australia/contents/autism (accessed August 2018).
3. Synergies Economic Consulting. Economic costs of autism spectrum disorder in Australia [Internet]. Report prepared for Autism Aspergers Advocacy Australia; 2011. http://a4.org.au/node/1064 (accessed August 2018).
4. Muris P, Steerneman P, Merckelbach H, et al. Comorbid anxiety symptoms in children with pervasive developmental disorders. *J Anxiety Disord* 1998;12:387-93.doi:10.1016/S0887-6185(98)00022-X
5. White SW, Oswald D, Ollendick T, et al. Anxiety in children and adolescents with autism spectrum disorders. *Clin Psychol Rev* 2009;29:216-29.doi:10.1016/j.cpr.2009.01.003 [published Online First: 25 January 2009].
6. Gadow KD, Devincent CJ, Pomeroy J, et al. Comparison of DSM-IV symptoms in elementary school-age children with PDD versus clinic and community samples. *Autism* 2005;9:392-415.doi:10.1177/1362361305056079
7. Simonoff E, Pickles A, Charman T, et al. Psychiatric disorders in children with autism spectrum disorders: prevalence, comorbidity, and associated factors in a population-derived sample. *J Am Acad Child Adolesc Psychiatry* 2008;47:921-9.doi:10.1097/CHI.0b013e318179964f
8. Beresford B, McDaid C, Parker A, et al. Pharmacological and non-pharmacological interventions for non-respiratory sleep disturbance in children with neurodisabilities: a systematic review. *Health Technol Assess* 2018;22:1-296.doi:10.3310/hta22600

For peer review only

9. Cohen S, Conduit R, Lockley SW, et al. The relationship between sleep and behavior in autism spectrum disorder (ASD): a review. *J Neurodev Disord* 2014;6:1-10.doi:10.1186/1866-1955-6-44 [published Online First: 11 December 2014].
10. Fletcher FE, Foster-Owens MD, Conduit R, et al. The developmental trajectory of parent-report and objective sleep profiles in autism spectrum disorder: Associations with anxiety and bedtime routines. *Autism* 2017;21:493-503.doi:10.1177/1362361316653365 [published Online First: 30 June 2016].
11. Hodge D, Carollo TM, Lewin M, et al. Sleep patterns in children with and without autism spectrum disorders: developmental comparisons. *Res Dev Disabil* 2014;35:1631-8.doi:10.1016/j.ridd.2014.03.037 [published Online First: 26 April 2014].
12. Mazurek MO, Dovgan K, Neumeyer AM, et al. Course and predictors of sleep and co-occurring problems in children with autism spectrum disorder. *J Autism Dev Disord* 2019;49:2101-15.doi:10.1007/s10803-019-03894-5 [published Online First: 25 January 2019].
13. Richdale AL, Schreck KA. Sleep problems in autism spectrum disorders: prevalence, nature, & possible biopsychosocial aetiologies. *Sleep Med Rev* 2009;13:403-11.doi:10.1016/j.smrv.2009.02.003 [published Online First: 24 April 2009].
14. Richdale AL, Prior MR. The sleep-wake rhythm in children with autism. *Eur Child Adolesc Psychiatry* 1995;4:175-86.doi:10.1007/BF01980456
15. Krakowiak P, Goodlin-Jones B, Hertz-Picciotto I, et al. Sleep problems in children with autism spectrum disorders, developmental delays, and typical development: a population-based study. *J Sleep Res* 2008;17:197-206.doi:10.1111/j.1365-2869.2008.00650.x
16. Liu X, Hubbard JA, Fabes RA, et al. Sleep disturbances and correlates of children with autism spectrum disorders. *Child Psychiatry Hum Dev* 2006;37:179-91.doi:10.1007/s10578-006-0028-3

For peer review only

17. Diaz-Roman A, Zhang J, Delorme R, et al. Sleep in youth with autism spectrum disorders: systematic review and meta-analysis of subjective and objective studies. *Evid Based Ment Health* 2018;21:146-54.doi:10.1136/ebmental-2018-300037 [published Online First: 27 October 2018].

18. Elrod MG, Hood BS. Sleep differences among children with autism spectrum disorders and typically developing peers: a meta-analysis. *J Dev Behav Pediatr* 2015;36:166-77.doi:10.1097/dbp.0000000000000140 [published Online First: 6 March 2015].

19. Sanberg SA, Kuhn BR, Kennedy AE. Outcomes of a behavioral intervention for sleep disturbances in children with autism spectrum disorder. *J Autism Dev Disord* 2018;48:4250-77.doi:10.1007/s10803-018-3644-4 [published Online First: 21 June 2018].

20. Souders MC, Zavodny S, Eriksen W, et al. Sleep in children with autism spectrum disorder. *Curr Psychiatry Rep* 2017;19:34.doi:10.1007/s11920-017-0782-x [published Online First: 13 May 2017].

21. Sivertsen B, Posserud M-B, Gillberg C, et al. Sleep problems in children with autism spectrum problems: a longitudinal population-based study. *Autism* 2012;16:139-50.doi:10.1177/1362361311404255 [published Online First: 8 April 2011].

22. Humphreys JS, Gringras P, Blair PS, et al. Sleep patterns in children with autistic spectrum disorders: a prospective cohort study. *Arch Dis Child* 2014;99:114-8.doi:10.1136/archdischild-2013-304083 [published Online First: 23 September 2013].

23. Johnson CR, Smith T, DeMand A, et al. Exploring sleep quality of young children with autism spectrum disorder and disruptive behaviors. *Sleep Med* 2018;44:61-6.doi:10.1016/j.sleep.2018.01.008

24. Levin A, Scher A. Sleep problems in young children with autism spectrum disorders: a study of parenting stress, mothers' sleep-related cognitions, and bedtime behaviors. *CNS*

For peer review only

Neurosci Ther 2016;22:921-7.doi:10.1111/cns.12651 [published Online First: 17 October 2016].

25. Richdale AL. Sleep disorders in autism and Asperger's syndrome. In: Stores G, Wiggs L, eds. Sleep Disturbances in Disorders of Development: Its Significance and Management. London: MacKeith Press 2001:181–91.
26. Leyfer OT, Folstein SE, Bacalman S, et al. Comorbid psychiatric disorders in children with autism: interview development and rates of disorders. *J Autism Dev Disord* 2006;36:849-61.doi:10.1007/s10803-006-0123-0
27. Goldman SE, McGrew S, Johnson KP, et al. Sleep is associated with problem behaviors in children and adolescents with autism spectrum disorders. *Res Autism Spectr Disord* 2011;5:1223-9.doi:10.1016/j.rasd.2011.01.010
28. Fadini CC, Lamônica DA, Fett-Conte AC, et al. Influence of sleep disorders on the behavior of individuals with autism spectrum disorder. *Front Hum Neurosci* 2015;9:347.doi:10.3389/fnhum.2015.00347 [published Online First: 18 June 2015].
29. Tudor ME, Hoffman CD, Sweeney DP. Children with autism: sleep problems and symptom severity. *Focus Autism Other Dev Disabl* 2012;27:254-62.doi:10.1177/1088357612457989
30. Malow BA, Marzec MS, McGrew SG, et al. Characterizing sleep in children with autism spectrum disorders: a multidimensional approach. *Sleep* 2006;29:1563-71.doi:10.1093/sleep/29.12.1563
31. Richdale AL, Baglin CL. Self-report and caregiver-report of sleep and psychopathology in children with high-functioning autism spectrum disorder: a pilot study. *Dev Neurorehabil* 2015;18:272-9.doi:10.3109/17518423.2013.829534 [published Online First: 2 October 2013].

For peer review only

32. Delahaye J, Kovacs E, Sikora D, et al. The relationship between health-related quality of life and sleep problems in children with autism spectrum disorders. *Res Autism Spectr Disord* 2014;8:292-303.doi:10.1016/j.rasd.2013.12.015

33. Taylor MA, Schreck KA, Mulick JA. Sleep disruption as a correlate to cognitive and adaptive behavior problems in autism spectrum disorders. *Res Dev Disabil* 2012;33:1408-17.doi:10.1016/j.ridd.2012.03.013 [published Online First: 21 April 2012].

34. Doo S, Wing YK. Sleep problems of children with pervasive developmental disorders: correlation with parental stress. *Dev Med Child Neurol* 2006;48:650-5.doi:10.1017/S001216220600137X

35. Honomichl RD, Goodlin-Jones BL, Burnham M, et al. Sleep patterns of children with pervasive developmental disorders. *J Autism Dev Disord* 2002;32:553-61.doi:10.1023/A:1021254914276

36. Hodge D, Hoffman CD, Sweeney DP, et al. Relationship between children's sleep and mental health in mothers of children with and without autism. *J Autism Dev Disord* 2013;43:956-63.doi:10.1007/s10803-012-1639-0 [published Online First: 30 August 2012].

37. Hoffman CD, Sweeney DP, Lopez-Wagner MC, et al. Children with autism: sleep problems and mothers' stress. *Focus Autism Other Dev Disabl* 2008;23:155-65.doi:10.1177/1088357608316271

38. Tilford JM, Payakachat N, Kuhlthau KA, et al. Treatment for sleep problems in children with autism and caregiver spillover effects. *J Autism Dev Disord* 2015;45:3613-23.doi:10.1007/s10803-015-2507-5 [published Online First: 1 July 2015].

39. Gringras P, Gamble C, Jones AP, et al. Melatonin for sleep problems in children with neurodevelopmental disorders: randomised double masked placebo controlled trial. *BMJ* 2012;345:e6664.doi:10.1136/bmj.e6664 [published Online First: 5 November 2012].

For peer review only

40. Vriend JL, Corkum PV, Moon EC, et al. Behavioral interventions for sleep problems in children with autism spectrum disorders: current findings and future directions. *J Pediatr Psychol* 2011;36:1017-29.doi:10.1093/jpepsy/jsr044 [published Online First: 10 July 2011].

41. Rigney G, Ali NS, Corkum P, et al. A systematic review to explore the feasibility of a behavioural sleep intervention for insomnia in children with neurodevelopmental disorders: a transdiagnostic approach. *Sleep Med Rev* 2018;41:244-54.doi:10.1016/j.smrv.2018.03.008 [published Online First: 31 March 2018].

42. Adkins KW, Molloy C, Weiss SK, et al. Effects of a standardized pamphlet on insomnia in children with autism spectrum disorders. *Pediatrics* 2012;130(Suppl 2):S139-S44.doi:10.1542/peds.2012-0900K

43. Johnson CR, Turner KS, Foldes E, et al. Behavioral parent training to address sleep disturbances in young children with autism spectrum disorder: a pilot trial. *Sleep Med* 2013;14:995-1004.doi:10.1016/j.sleep.2013.05.013 [published Online First: 27 August 2013].

44. Wiggs L, Stores G. Behavioural treatment for sleep problems in children with severe learning disabilities and challenging daytime behaviour: effect on sleep patterns of mother and child. *J Sleep Res* 1998;7:119-26.doi:10.1046/j.1365-2869.1998.00107.x

45. Simonds JF, Parraga H. Prevalence of sleep disorders and sleep behaviors in children and adolescents. *Journal of the American Academy of Child & Adolescent Psychiatry* 1982;21:383-8.doi:10.1016/S0002-7138(09)60942-0

46. Malow BA, Adkins KW, Reynolds A, et al. Parent-based sleep education for children with autism spectrum disorders. *J Autism Dev Disord* 2014;44:216-28.doi:10.1007/s10803-013-1866-z [published Online First: 11 June 2013].

For peer review only

47. Hiscock H, Sciberras E, Mensah F, et al. Impact of a behavioral sleep intervention on ADHD symptoms, child sleep and parent health: a randomized controlled trial. *BMJ* 2015;350:h68.doi:10.1136/bmj.h68 [published Online First: 20 January 2015].

48. Papadopoulos N, Sciberras E, Hiscock H, et al. The Efficacy of a Brief Behavioral Sleep Intervention in School-Aged Children With ADHD and Comorbid Autism Spectrum Disorder. *J Atten Disord* 2019;23:341-50.doi:10.1177/1087054714568565 [published Online First: 2015/02/04].

49. Owens JA, Spirito A, McGuinn M. The Children's Sleep Habits Questionnaire (CSHQ): psychometric properties of a survey instrument for school-aged children. *Sleep* 2000;23:1043-51.doi:10.1093/sleep/23.8.1d

50. Chan A-W, Tetzlaff JM, Altman DG, et al. SPIRIT 2013 statement: defining standard protocol items for clinical trials. *Ann Intern Med* 2013;158:200-7.doi:10.7326/0003-4819-158-3-201302050-00583

51. American Academy of Sleep Medicine. International Classification of Sleep Disorders (ISCD-3). 3rd ed. Darian, IL: American Academy of Sleep Medicine 2014.

52. American Psychiatric Association. Diagnostic and Statistical Manual of Mental Disorders. 4th ed. Washington, DC: APA 2000.

53. Rutter M, Bailey A, Lord C. The Social Communication Questionnaire. Los Angeles: Western Psychological Services 2003.

54. Harris PA, Taylor R, Thielke R, et al. Research electronic data capture (REDCap): a metadata-driven methodology and workflow process for providing translational research informatics support. *J Biomed Inform* 2009;42:377-81.doi:10.1016/j.jbi.2008.08.010 [published Online First: 30 September 2008].

For peer review only

55. Kim YS, Leventhal BL, Koh Y-J, et al. Prevalence of autism spectrum disorders in a total population sample. *Am J Psychiatry* 2011;168:904-12.doi:10.1176/appi.ajp.2011.10101532 [published Online First: 9 May 2011].

56. Sivaratnam CSea. Brief report: assessment of the social-emotional profile in children with autism spectrum disorders using a novel comic strip task. *J Autism Dev Disord* 2012;42:2505-12.doi:10.1007/s10803-012-1498-8 [published Online First: 15 March 2012].

57. Souders MC, Mason TBA, Valladares O, et al. Sleep behaviors and sleep quality in children with autism spectrum disorders. *Sleep* 2009;32:1566-78.doi:10.1093/sleep/32.12.1566

58. Rinehart N, Sciberras E, Hiscock H, et al. Tailoring a brief sleep intervention for autism: a randomised controlled trial. [Unpublished peer reviewed grant proposal]. National Health and Medical Research Council 2015.

59. Australian Bureau of Statistics. Census of population and housing: Socio-economic indexes for areas (SEIFA), Australia, 2016 (2033.0.55.001) [Internet]. Canberra: ABS; 2018. https://www.abs.gov.au/ausstats/abs@.nsf/mf/2033.0.55.001 (accessed February 2019).

60. Benjamini Y, Hochberg Y. Controlling The False Discovery Rate - A Practical And Powerful Approach To Multiple Testing1995.

61. Sauzet O, Peacock JL. Binomial outcomes in dataset with some clusters of size two: can the dependence of twins be accounted for? A simulation study comparing the reliability of statistical methods based on a dataset of preterm infants. *BMC Med Res Methodol* 2017;17:110.doi:10.1186/s12874-017-0369-6 [published Online First: 20 July 2017].

62. Stevens K. Valuation of the Child Health Utility 9D Index. *PharmacoEconomics* 2012;30:729-47.doi:10.2165/11599120-000000000-00000

For peer review only

63. Abel E, Kim SY, Kellerman AM, et al. Recommendations for identifying sleep problems and treatment resources for children with autism spectrum disorder. *Behav Anal Pract* 2017;10:261-9.doi:10.1007/s40617-016-0158-4 [published Online First: 21 November 2016].

64. Malow BA, Byars K, Johnson K, et al. A practice pathway for the identification, evaluation, and management of insomnia in children and adolescents with autism spectrum disorders. *Pediatrics* 2012;130 (Suppl 2):S106-S24.doi:10.1542/peds.2012-0900I

65. National Institute for Health and Care Excellence. Autism spectrum disorder in under 19s: support and management (NICE Clinical guideline 170) [Internet]. London: NICE; 2013. http://guidance.nice.org.uk/CG170 (accessed August 2018).

66. Hiscock H, Canterford L, Ukoumunne OC, et al. Adverse associations of sleep problems in Australian preschoolers: national population study. *Pediatrics* 2007;119:86-93.doi:10.1542/peds.2006-1757

67. Corsello C, Hus V, Pickles A, et al. Between a ROC and a hard place: decision making and making decisions about using the SCQ. *J Child Psychol Psychiatry* 2007;48:932-40.doi:10.1111/j.1469-7610.2007.01762.x

68. Owens JA, Spirito A, McGuinn M, et al. Sleep habits and sleep disturbance in elementary school-aged children. *J Dev Behav Pediatr* 2000;21:27-36.doi:10.1097/00004703-200002000-00005

69. Einfield SL, Tonge BJ. Manual for the Developmental Behaviour Checklist: Primary Carer Version (DBC-P) and Teacher (DBC-T) Version. Melbourne, AU: Monash University Centre for Developmental Psychiatry and Psychology 2002.

For peer review only

70. Goodman R. Psychometric properties of the strengths and difficulties questionnaire. *J Am Acad Child Adolesc Psychiatry* 2001;40:1337-45.doi:10.1097/00004583-200111000-00015

71. Wilkinson GS, Robertson GJ. WRAT 4: Wide Range Achievement Test; Professional Manual. 4 ed. Lutz, FL: Psychological Assessment Resources, Inc 2006.

72. Gershon RC, Cella D, Fox NA, et al. Assessment of neurological and behavioural function: the NIH Toolbox. *Lancet Neurol* 2010;9:138-9.doi:10.1016/S1474-4422(09)70335-7

73. Kessler RC, Barker PR, Colpe LJ, et al. Screening for serious mental illness in the general population. *Arch Gen Psychiatry* 2003;60:184-9.doi:10.1001/archpsyc.60.2.184

74. Abidin RR. Parenting Stress Index. 4 ed. Lutx, FL: PAR 2012.

75. Richardson J, Hawthorne G. The Australian Quality of Life (AQoL) instrument: psychometric properties of the descriptive system and initial validation. *Australian Studies of Health Service Administration* 1998;85:315-42.

For peer review only

	STUDY PERIOD				
	Enrolment		Post-randomization		Close out
TIMEPOINT	*Baseline*	Randomization	*3m*	*6m*	
**ENROLMENT:**					
Eligibility screening	X				
Informed consent	X				
Baseline parent survey[a]	X				
Randomization		X			
**INTERVENTIONS:**					
Sleeping sound intervention					
Treatment as usual					
**ASSESSMENTS:**					
Parent survey[a]	X		X	X	
Teacher survey[a]	X		X	X	
Child assessment[a]				X	
Medicare/PBS linkage					X
NAPLAN linkage					X

Note. [a] Refer to Table 3 for the specific measures included.
Figure 1. SPIRIT timeline

For peer review only

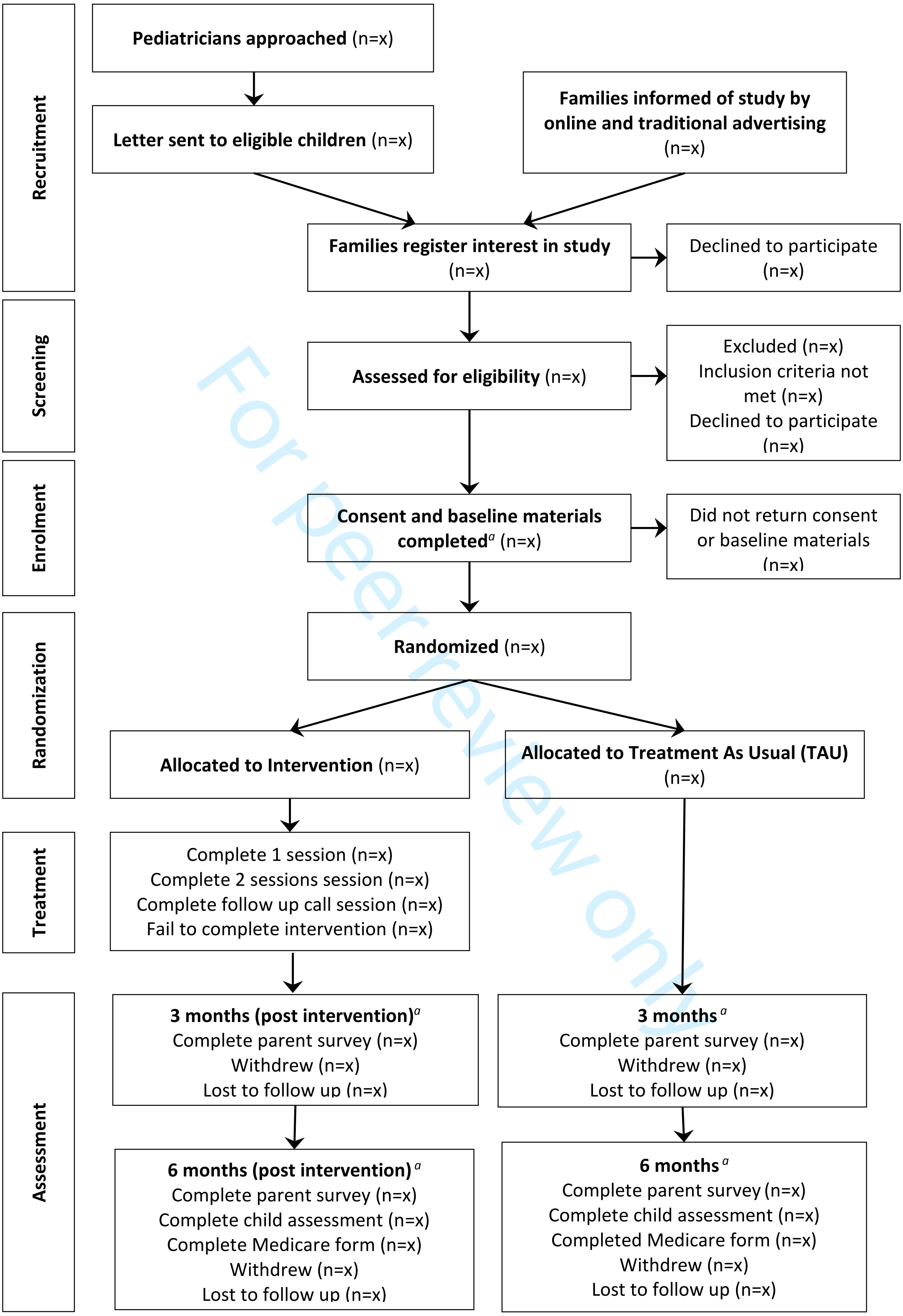

Note. [a] Complete teacher survey if applicable

Figure 2: Flow of participants

For peer review only

Cluster effect 1 - paediatrician					Cluster effect 2 - individual						
ICC	Mean	SD	CV	DE1 = 1+(n-1)ρ	ICC	Mean	SD	CV	DE2 = 1+(n-1)ρ	z_beta (80%)	z_alpha (.05, two tailed)
0.08	4	3	0.75	1.24	0.2	2	1	0.50	1.20	0.84	1.96

For peer review only

Attrition	d	var_d	n_total=2*((2*sigma^2 *(z_beta+z_alpha)^2)/(d^2))	Sample size (total)	DE1 adjusted N	DE2 adjusted N	Attrition adjusted	Per group
0.20	0.50	1		125	156	187	233	117

For peer review only

Appendix

Participant Information Sheet and Consent Form – Parent Version

Participant Information Sheet/Consent Form – Parent/Guardian

Interventional Study - *Parent/Guardian consenting on behalf of participant*

**HREC Project Number:**	HREC/16/RCHM/73		
**Research Project Title:**	**Sleeping Sound with Autism Spectrum Disorder (ASD):** Tailoring a behavioural sleep intervention for children with ASD: a randomized controlled trial		
**Principal Researcher:**	Professor Nicole Rinehart, School of Psychology, Deakin University		
**Version Number:**	7	**Version Date:**	17/9/2017

Thank you for taking the time to read this **Parent/Guardian Information Statement and Consent Form**. We would like to invite your child to participate in a research project that is explained below. This document is 5 pages long. Please make sure you have all the pages.

What is an Information Statement?
These pages tell you about the research project. It explains to you clearly and openly all the steps and procedures of the project. The information is to help you decide whether or not you would like your child to take part in the research. Please read this Information Statement carefully.

Before you decide if you want your child to take part or not, you can ask us any questions you have about the project. You may want to talk about the project with your family, friends or health care worker.

Important things you need to know
- It is your choice whether or not your child can take part in the research. You do not have to agree if you do not want to
- If you decide you do not want your child to take part, it will not affect the treatment and care your child gets at The Royal Children's Hospital or with your paediatrician.

If you would like your child to take part in the research project, please sign the consent form at the end of this information statement. By signing the consent form you are telling us that you:

- understand what you have read
- had a chance to ask questions and received satisfactory answers
- consent to your child taking part in the project

We will give you a copy of this information and consent form to keep.

---

1. What is the research project about?

This research project is about sleep in children with an Autism Spectrum Disorder (ASD). Up to 85% of children with ASD have sleep problems, such as difficulty falling asleep, resisting

For peer review only - http://bmjopen.bmj.com/site/about/guidelines.xhtml

going to bed and being tired in the morning. We know sleep problems in children with ASD can be related to poorer child and family functioning.

In 2013, we ran a small study that looked at whether a sleep program improved sleep, child and family wellbeing in children with ASD. We found that a 2-3 session sleep program improved these outcomes. This program has already been shown to be helpful for children with ADHD.

This year, we aim to offer this sleep program to a larger group of children with ASD who have sleep problems. To know whether this program helps, we need to compare children who receive the program with children who do not. We hope about 235 children with ASD and their families will take part in this project.

To take part in the study, children need to be aged between 5 and 12 years of age (or 13 years of age and attending primary school), have problems with sleeping and a diagnosis of ASD without an intellectual disability.

2. Who is funding this research project?

This research is fully funded by the National Health and Medical Research Council.

3. Why is my child being asked to take part?

We are asking you and your child to take part because your child has ASD and is aged between 5 and 12 years (or is 13 years and attending primary school).

4. What does my child need to do in this research project?

Start of study
We will telephone you to tell you more about the project and see if your child is eligible. This will involve asking you some questions about your child's sleep, autism symptoms, wellbeing and daily functioning. This call will be conducted in two phone calls, each of which will take between 20 minutes and 40 minutes. We will also ask to contact your child's paediatrician for confirmation of ASD diagnosis. If your child is not eligible for the study, the data you provide will only be reported in summary form (e.g. a total of 7 children were not eligible for the study as they had an intellectual disability).

If your child is eligible to take part, we will ask you to complete a survey about your child's autism and sleep, and about your child and family's wellbeing. This will take about 20 minutes to do and you can complete it online or send it back to us in the reply-paid envelope provided if you are completing it in hard copy. Once we have received your survey, we will randomly place you in one of two groups. This will be done by chance, similar to tossing a coin, so you will have an equal chance of being in either group. We will send you a letter to let you know which group you have been placed in. What you will do next will depend on which group you are allocated to.

- **Usual Care Group**

If you are in the Usual Care Group, we will contact you again in 3, 6- and 12-months' time and ask you to complete surveys about your child's autism and sleep, and child and family wellbeing. Each survey will take about 20 minutes to fill out.

For peer review only

- **Sleep Program Group**

If you are allocated to the Sleep Program Group, we will offer you two 50-minute visits and a follow up phone call with a clinician (psychologist or paediatrician) who has been trained to help you manage your child's sleep problems. At the sleep visits, the clinician will assess your child's sleep and provide you with some sleep strategies. These visits are free. These visits are done privately with the clinician, yourself and your child at Deakin University (Burwood or Geelong), the Royal Children's Hospital/MCRI (Parkville), or your paediatrician's office – there will be no other families at the visit. We will then send you surveys to complete in 3, 6- and 12-months' time. Each survey asks about your child's ASD and sleep, and child and family wellbeing and will take about 20 minutes to fill out.

6 months after start of study – participants in both groups

We will arrange a time to complete a face-to-face learning assessment with your child. It will take about 60 minutes to do. The assessment is non-invasive and most children enjoy doing it. We can visit you at your home to do this assessment or you can visit us at The Royal Children's Hospital in Melbourne, or Deakin University in Burwood or Geelong. We will provide you with a brief report from this assessment if requested.

5. Optional consent

We would like you to consider the following optional parts of this project. You may agree to none, some or all optional parts. However, it is your decision, and if you choose not to agree to any parts, it will not affect your participation in the rest of the study. Please complete the optional consent tick boxes on the consent form to record your decision for each option.

A. **Contacting your child's school teacher:** We would like to ask your child's teacher to fill out a short survey about your child's behaviour to find out if sleep affects your child at school. We will ask them to complete the survey on four occasions. We will contact the kindergarten director or school principal and gain their approval to ask your child's teacher to do this. We will not refer to ASD in the information we send to the teacher or the principal/director. Instead, we will say that this is a project about managing sleep problems in children. The information provided by the teacher in the survey will not be shared with you or your child.

B. **NAPLAN.** We would like your permission to get your child's NAPLAN results. Your child's NAPLAN results will be de-identified and stored in a password-secured database, only accessible by the research team. Please indicate on the consent form if you consent to us getting your child's NAPLAN results.

C. **Assessment records.** We would like to access the results of any assessments related to ASD which your child may have done in the past, including autism assessments, IQ, adaptive functioning and language assessments. We will use this information to help us understand whether the intervention is useful for children with a range of abilities. If you do not have copies of these reports yourself, with your permission we will request them from health professionals or hospitals your child has visited.

D. **Re-contact for future research:** We would like to offer you the chance to hear about new research projects that are related to this project. By ticking this consent you are only consenting to hear about new projects only. You can decide at the time whether or not you want your child to take part in any future project.

E. **Medicare and Pharmaceutical Benefits Scheme (PBS):** We would like to access your child's complete Medicare and PBS data. This will help us to see whether families who get the sleep program access fewer health services and medicines than those who

For peer review only

do not get the program. Medicare collects information on your child's doctors' visits and the associated costs, while the PBS collects information on the prescription medications you have filled at pharmacies for your child. We will ask you to fill out this additional consent form at the time of your child's 6 month follow up, which will allow us to access this data as outlined on the back of the additional consent form we will give you at that time. This consent form will then be sent securely to the Department of Human Services who holds this information confidentially.

6. Can my child withdraw from the project?

If you give your consent and change your mind, your child can withdraw from the project. If you decide to withdraw, please notify the research team. You do not need to tell us the reason why you or your child want to stop being in the project. If your child leaves the project we will use any information already collected unless you tell us not to do so.

7. What are the possible benefits for my child and other people in the future?

If you are in the **Sleep Program Group**, the clinician will provide strategies and information to help you with your child's sleep problem. If your child has other difficulties other than sleep problems (e.g. anxiety, ADHD symptoms), these will not be directly addressed by the Sleeping Sound intervention – the study clinician will encourage you to discuss these problems with your treating paediatrician. We hope that your child will have fewer sleep problems and this may help his/her behaviour as well as your child and family's wellbeing.

If you are in the **Usual Care Group**, you and your child will not receive any direct benefit.

We are doing this project to see if this sleep program works or not. This means that we can't guarantee that your child and family will benefit, even if you are in the **Sleep Program Group.**

Whether you receive the sleep program or not, your participation will help us learn whether the sleep program is helpful for families of children with ASD. If it is helpful, then the project will be the first to provide information which may help future children with sleep problems and ASD.

8. What are the possible risks, side-effects, discomforts and/or inconveniences?

We do not anticipate risks, side-effects or discomforts for you or your child. In our previous *Sleeping Sound with ADHD* project, parents did not report any side-effects or discomforts in their feedback surveys.

9. What will be done to make sure my child's information is confidential?

In this study we will collect and use personal and health information about your child for research purposes. Any information we collect that can identify your child will be treated as confidential. It will be used only in this project, unless otherwise specified. We can disclose the information only with your permission, except as required by law. All information will be stored securely at Deakin University. Information will be kept electronically on a secure Deakin University server, in a password protected database (REDCap) or password protected computer. All hard copy consent forms, surveys and assessments will be stored securely in a locked cabinet at Deakin University.

For peer review only

The following people may access information collected as part of this research project:

- The research team involved with this project
- The Royal Children's Hospital Human Research Ethics Committee

The information will be re-identifiable. This means that we will remove your child's name and give the information a special code number. Only the research team can match your child's name to their code number, if it is necessary to do so.

Information will be kept until the youngest participant turns 25 years old. The research information may be destroyed or kept indefinitely in secure storage after this time. However, Medicare/PBS data will be kept for 15 years only from the date of collection. After this time, the consent paper copies will be shredded in a manner that makes re-identification of the data impossible, and the electronic Medicare and PBS data will be deleted.

In accordance with relevant Australian and/or Victorian privacy and other relevant laws, you have the right to access and correct the information we collect and store about your child. Please contact us if you would like to access this information.

At the end of the study, results may be presented at conferences or published in medical journals. This will be done in such a way that your child cannot be identified.

10. Will we be informed of the results when the research project is finished?

Participants in the Sleep Program group and the Usual Care group will receive a summary of the results at the end of this project, which will say whether the intervention was successful. This summary will give overall results of this project – individual families will not be identified. Participants in the Usual Care group **will not** be offered the Sleeping Sound intervention after completion of the study.

11. Who should I contact for more information?

If you would like more information about the project or if you need to speak to a member of the research team in an emergency please contact:

**Name:**	Susannah Bellows
**Contact telephone:**	9246 8937
**Email:**	sleepingsound@deakin.edu.au

If you have any concerns and/or complaints about the project, the way it is being conducted or your child's rights as a research participant, and would like to speak to someone independent of the project, please contact: Director, Research Ethics & Governance, The Royal Children's Hospital Melbourne on telephone: (03) 9345 5044.

For peer review only

CONSENT FORM

**HREC Project Number:**	HREC/16/RCHM/73
**Research Project Title:**	Sleeping Sound with Autism Spectrum Disorder (ASD):<br>Tailoring a behavioural sleep intervention for children with ASD: a randomized controlled trial
**Version Number:** 7	**Version Date:** 17/09/2017

- I have read, or someone has read to me in a language that I understand, the information statement version listed above and I understand its contents.
- I believe I understand the purpose, extent and possible risks of our involvement in this project.
- I voluntarily consent for me and my child to take part in this research project.
- I have had an opportunity to ask questions and I am satisfied with the answers I have received.
- I understand that this project has been approved by The Royal Children's Hospital Melbourne Human Research Ethics Committee and will be carried out in line with the National Statement on Ethical Conduct in Human Research (2007) – including all updates.
- I understand I will receive a copy of this Information Statement and Consent Form.

OPTIONAL CONSENT

☐ I do	☐ I do not	consent for the researchers to contact my child's school teacher to complete a survey
☐ I do	☐ I do not	consent for VCAA (Victorian Curriculum and Assessment Authority) to release my child's years 3, 5, and 7 NAPLAN results to the Sleeping Sound with ASD Project.
☐ I do	☐ I do not	consent for the researchers to access copies of my child's assessments and medical records related to autism
☐ I do	☐ I do not	consent to be contacted about future research projects that are related to this project.
☐ I do	☐ I do not	consent to the storage and use of my child's information in future ethically-approved research related to this research project.

____
Child's Name

Parent/Guardian Name	Parent/Guardian Signature	Date
Name of Witness to Parent/Guardian's Signature	Witness Signature	Date

For peer review only

Declaration by researcher: I have explained the project to the parent/guardian who has signed above, and believe that they understand the purpose, extent and possible risks of their and their child's involvement in this project.

Research Team Member Name	Research Team Member Signature	Date

Note: All parties signing the Consent Form must date their own signature.

For peer review only

SPIRIT

STANDARD PROTOCOL ITEMS: RECOMMENDATIONS FOR INTERVENTIONAL TRIALS

SPIRIT 2013 Checklist: Recommended items to address in a clinical trial protocol and related documents*

Section/item	Item No	Description	Addressed on page number
**Administrative information**			
Title	1	Descriptive title identifying the study design, population, interventions, and, if applicable, trial acronym	______1______
Trial registration	2a	Trial identifier and registry name. If not yet registered, name of intended registry	______3______
	2b	All items from the World Health Organization Trial Registration Data Set	_____N/A_____
Protocol version	3	Date and version identifier	______N/A____
Funding	4	Sources and types of financial, material, and other support	_____ 26______
Roles and responsibilities	5a	Names, affiliations, and roles of protocol contributors	_____1,26______
	5b	Name and contact information for the trial sponsor	_____N/A____
	5c	Role of study sponsor and funders, if any, in study design; collection, management, analysis, and interpretation of data; writing of the report; and the decision to submit the report for publication, including whether they will have ultimate authority over any of these activities	_____N/A______
	5d	Composition, roles, and responsibilities of the coordinating centre, steering committee, endpoint adjudication committee, data management team, and other individuals or groups overseeing the trial, if applicable (see Item 21a for data monitoring committee)	_____N/A______

For peer review only

For peer review only - http://bmjopen.bmj.com/site/about/guidelines.xhtml

Introduction

Background and rationale	6a	Description of research question and justification for undertaking the trial, including summary of relevant studies (published and unpublished) examining benefits and harms for each intervention	____4-10______
	6b	Explanation for choice of comparators	____8_________
Objectives	7	Specific objectives or hypotheses	____8-9________
Trial design	8	Description of trial design including type of trial (eg, parallel group, crossover, factorial, single group), allocation ratio, and framework (eg, superiority, equivalence, noninferiority, exploratory)	____8_____

Methods: Participants, interventions, and outcomes

Study setting	9	Description of study settings (eg, community clinic, academic hospital) and list of countries where data will be collected. Reference to where list of study sites can be obtained	____10, 13______
Eligibility criteria	10	Inclusion and exclusion criteria for participants. If applicable, eligibility criteria for study centres and individuals who will perform the interventions (eg, surgeons, psychotherapists)	__10-12, 22___
Interventions	11a	Interventions for each group with sufficient detail to allow replication, including how and when they will be administered	____13-15______
	11b	Criteria for discontinuing or modifying allocated interventions for a given trial participant (eg, drug dose change in response to harms, participant request, or improving/worsening disease)	____N/A_____
	11c	Strategies to improve adherence to intervention protocols, and any procedures for monitoring adherence (eg, drug tablet return, laboratory tests)	_____17_______
	11d	Relevant concomitant care and interventions that are permitted or prohibited during the trial	_____12_______
Outcomes	12	Primary, secondary, and other outcomes, including the specific measurement variable (eg, systolic blood pressure), analysis metric (eg, change from baseline, final value, time to event), method of aggregation (eg, median, proportion), and time point for each outcome. Explanation of the clinical relevance of chosen efficacy and harm outcomes is strongly recommended	_____9, 15-16 __
Participant timeline	13	Time schedule of enrolment, interventions (including any run-ins and washouts), assessments, and visits for participants. A schematic diagram is highly recommended (see Figure)	_____11-15, Fig1+2

For peer review only - http://bmjopen.bmj.com/site/about/guidelines.xhtml

Sample size	14	Estimated number of participants needed to achieve study objectives and how it was determined, including clinical and statistical assumptions supporting any sample size calculations	_____16-17_____
Recruitment	15	Strategies for achieving adequate participant enrolment to reach target sample size	_____11-12_____

Methods: Assignment of interventions (for controlled trials)

Allocation:			
Sequence generation	16a	Method of generating the allocation sequence (eg, computer-generated random numbers), and list of any factors for stratification. To reduce predictability of a random sequence, details of any planned restriction (eg, blocking) should be provided in a separate document that is unavailable to those who enrol participants or assign interventions	_____12______
Allocation concealment mechanism	16b	Mechanism of implementing the allocation sequence (eg, central telephone; sequentially numbered, opaque, sealed envelopes), describing any steps to conceal the sequence until interventions are assigned	_____12______
Implementation	16c	Who will generate the allocation sequence, who will enrol participants, and who will assign participants to interventions	_____12-13___
Blinding (masking)	17a	Who will be blinded after assignment to interventions (eg, trial participants, care providers, outcome assessors, data analysts), and how	_____16______
	17b	If blinded, circumstances under which unblinding is permissible, and procedure for revealing a participant's allocated intervention during the trial	_____16______

Methods: Data collection, management, and analysis

Data collection methods	18a	Plans for assessment and collection of outcome, baseline, and other trial data, including any related processes to promote data quality (eg, duplicate measurements, training of assessors) and a description of study instruments (eg, questionnaires, laboratory tests) along with their reliability and validity, if known. Reference to where data collection forms can be found, if not in the protocol	11-12, 15-17, 24-25
	18b	Plans to promote participant retention and complete follow-up, including list of any outcome data to be collected for participants who discontinue or deviate from intervention protocols	______17,19_____

Data management	19	Plans for data entry, coding, security, and storage, including any related processes to promote data quality (eg, double data entry; range checks for data values). Reference to where details of data management procedures can be found, if not in the protocol	______21_______
Statistical methods	20a	Statistical methods for analysing primary and secondary outcomes. Reference to where other details of the statistical analysis plan can be found, if not in the protocol	______17-20____
	20b	Methods for any additional analyses (eg, subgroup and adjusted analyses)	______18-20____
	20c	Definition of analysis population relating to protocol non-adherence (eg, as randomised analysis), and any statistical methods to handle missing data (eg, multiple imputation)	______17-18____
**Methods: Monitoring**			
Data monitoring	21a	Composition of data monitoring committee (DMC); summary of its role and reporting structure; statement of whether it is independent from the sponsor and competing interests; and reference to where further details about its charter can be found, if not in the protocol. Alternatively, an explanation of why a DMC is not needed	_____N/A______
	21b	Description of any interim analyses and stopping guidelines, including who will have access to these interim results and make the final decision to terminate the trial	_____N/A______
Harms	22	Plans for collecting, assessing, reporting, and managing solicited and spontaneously reported adverse events and other unintended effects of trial interventions or trial conduct	______15______
Auditing	23	Frequency and procedures for auditing trial conduct, if any, and whether the process will be independent from investigators and the sponsor	_____N/A______
**Ethics and dissemination**			
Research ethics approval	24	Plans for seeking research ethics committee/institutional review board (REC/IRB) approval	_____21_______
Protocol amendments	25	Plans for communicating important protocol modifications (eg, changes to eligibility criteria, outcomes, analyses) to relevant parties (eg, investigators, REC/IRBs, trial participants, trial registries, journals, regulators)	____ 21________

For peer review only - http://bmjopen.bmj.com/site/about/guidelines.xhtml

Consent or assent	26a	Who will obtain informed consent or assent from potential trial participants or authorised surrogates, and how (see Item 32)	___11________
	26b	Additional consent provisions for collection and use of participant data and biological specimens in ancillary studies, if applicable	___12-13__________
Confidentiality	27	How personal information about potential and enrolled participants will be collected, shared, and maintained in order to protect confidentiality before, during, and after the trial	___21_________
Declaration of interests	28	Financial and other competing interests for principal investigators for the overall trial and each study site	___26-27________
Access to data	29	Statement of who will have access to the final trial dataset, and disclosure of contractual agreements that limit such access for investigators	___21_________
Ancillary and post-trial care	30	Provisions, if any, for ancillary and post-trial care, and for compensation to those who suffer harm from trial participation	___N/A________
Dissemination policy	31a	Plans for investigators and sponsor to communicate trial results to participants, healthcare professionals, the public, and other relevant groups (eg, via publication, reporting in results databases, or other data sharing arrangements), including any publication restrictions	___22________
	31b	Authorship eligibility guidelines and any intended use of professional writers	____2________
	31c	Plans, if any, for granting public access to the full protocol, participant-level dataset, and statistical code	___N/A_______
**Appendices**			
Informed consent materials	32	Model consent form and other related documentation given to participants and authorised surrogates	____Appendix____
Biological specimens	33	Plans for collection, laboratory evaluation, and storage of biological specimens for genetic or molecular analysis in the current trial and for future use in ancillary studies, if applicable	____N/A_______

*It is strongly recommended that this checklist be read in conjunction with the SPIRIT 2013 Explanation & Elaboration for important clarification on the items. Amendments to the protocol should be tracked and dated. The SPIRIT checklist is copyrighted by the SPIRIT Group under the Creative Commons "Attribution-NonCommercial-NoDerivs 3.0 Unported" license.
